# Supplementary figures and images for: An arms race between 5’ppp-RNA virus and its alternative recognition receptor MDA5 in RIG-I-lost teleost fish (part 1 of 2)
Source: eLife. 2024 Sep 30;13:RP94898. doi: 10.7554/eLife.94898 (PMC11441976; doi:10.7554/eLife.94898)

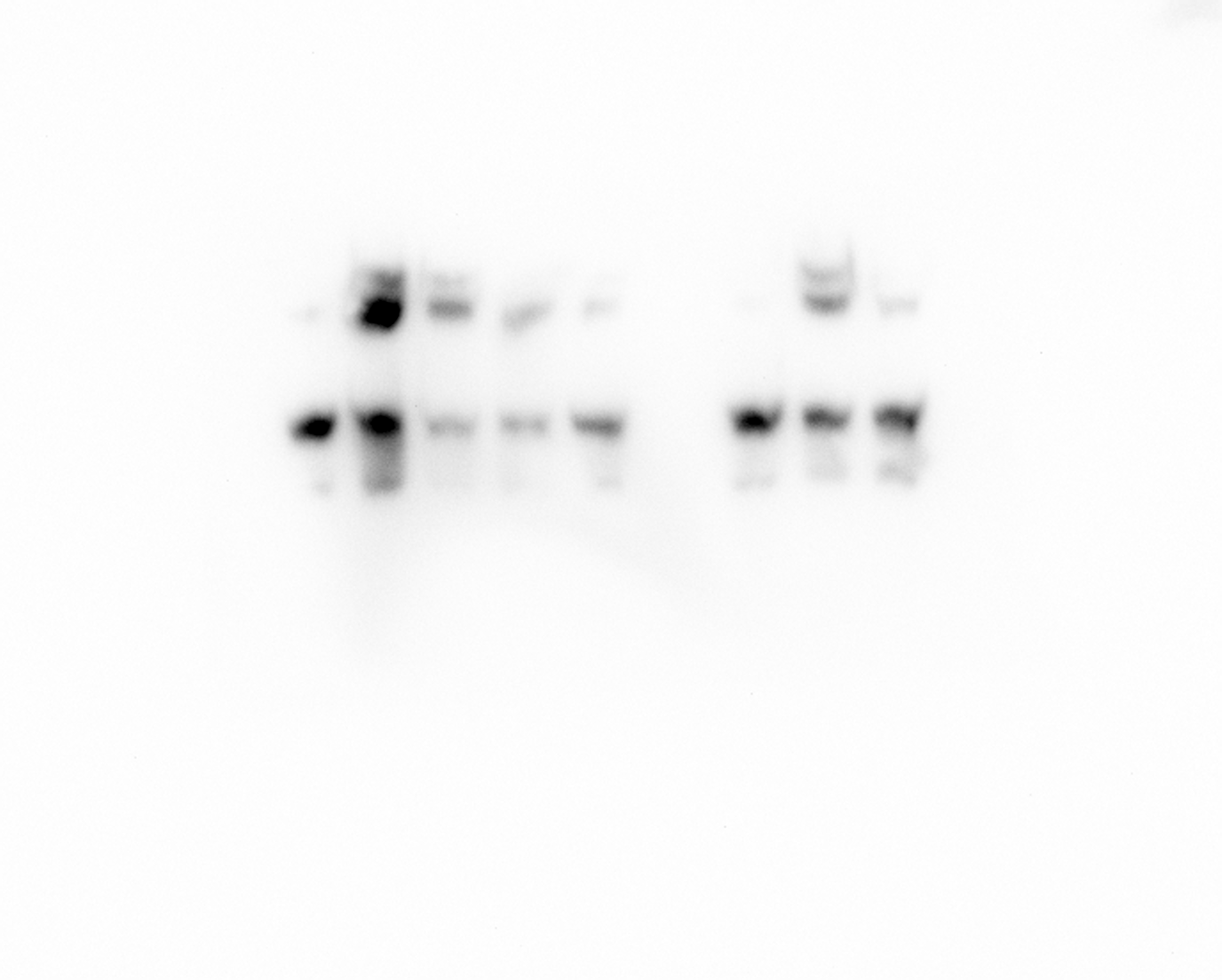

Supplement: Figure 1—source data 2. [file elife-94898-fig1-data2.zip › Fig1C-HA.Tif]

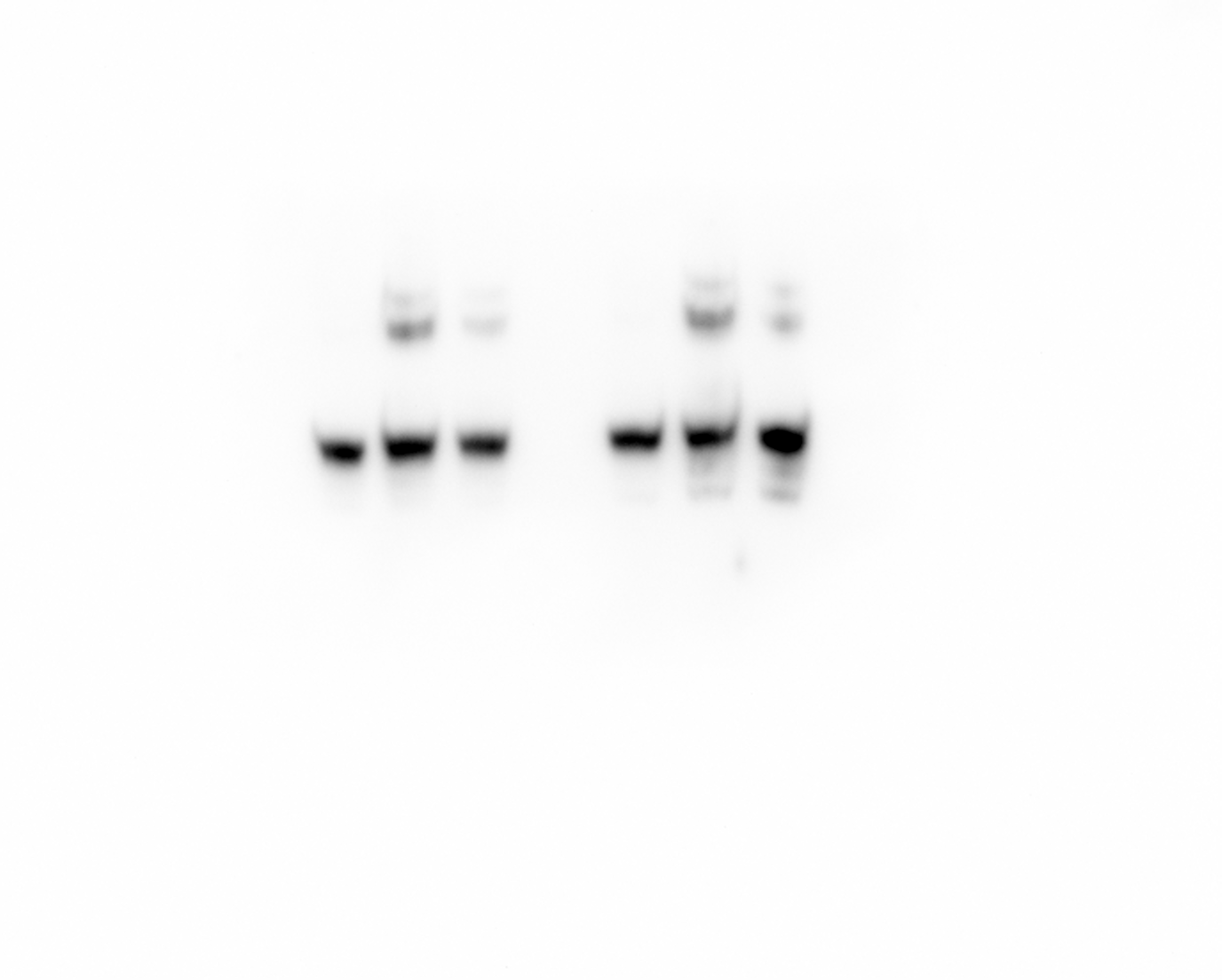

Supplement: Figure 1—source data 2. [file elife-94898-fig1-data2.zip › Fig1D-IRF3.Tif]

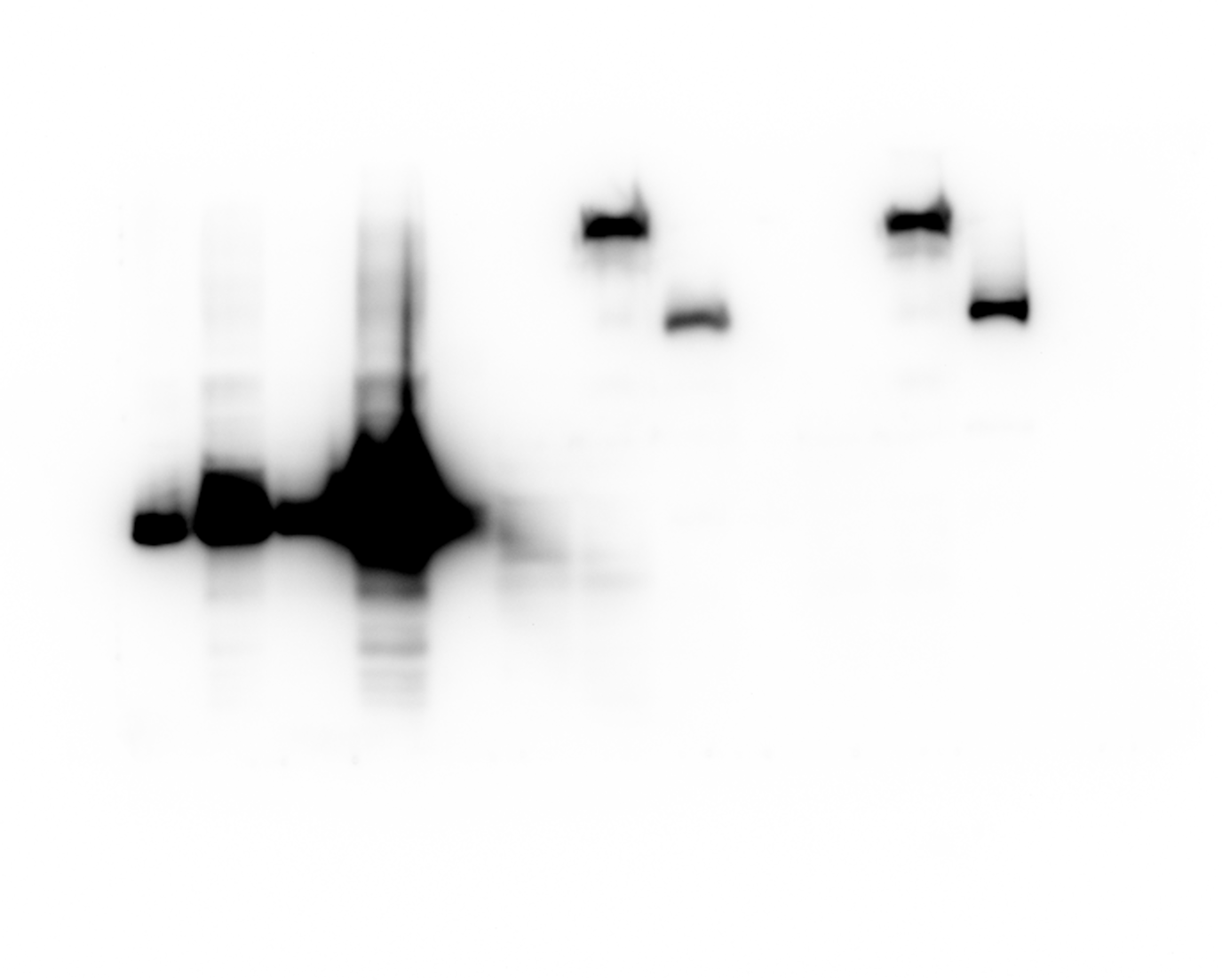

Supplement: Figure 1—source data 2. [file elife-94898-fig1-data2.zip › Fig1G-Flag.Tif]

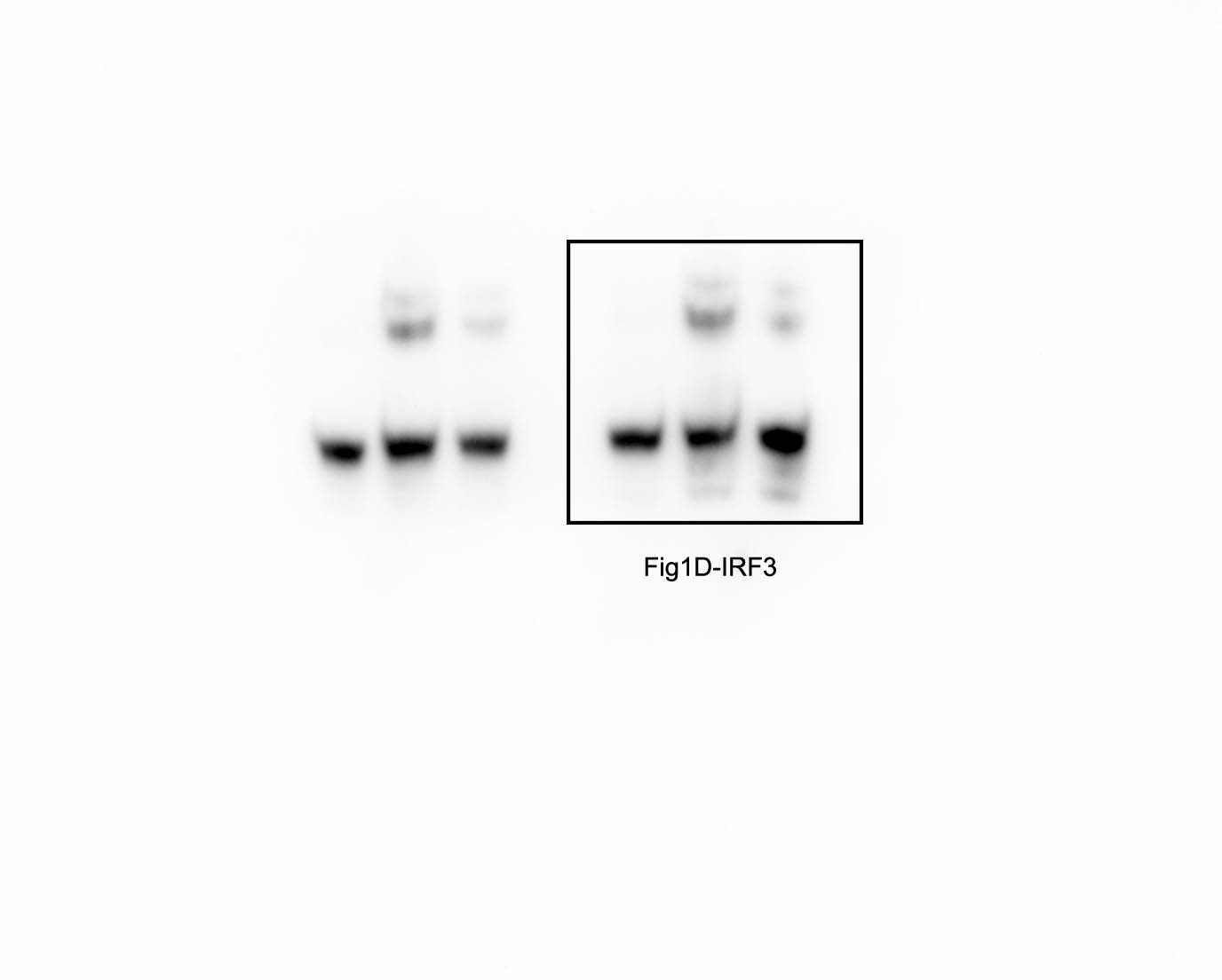

Supplement: Figure 1—source data 3. [file elife-94898-fig1-data3.zip › Fig1D-IRF3.jpg]

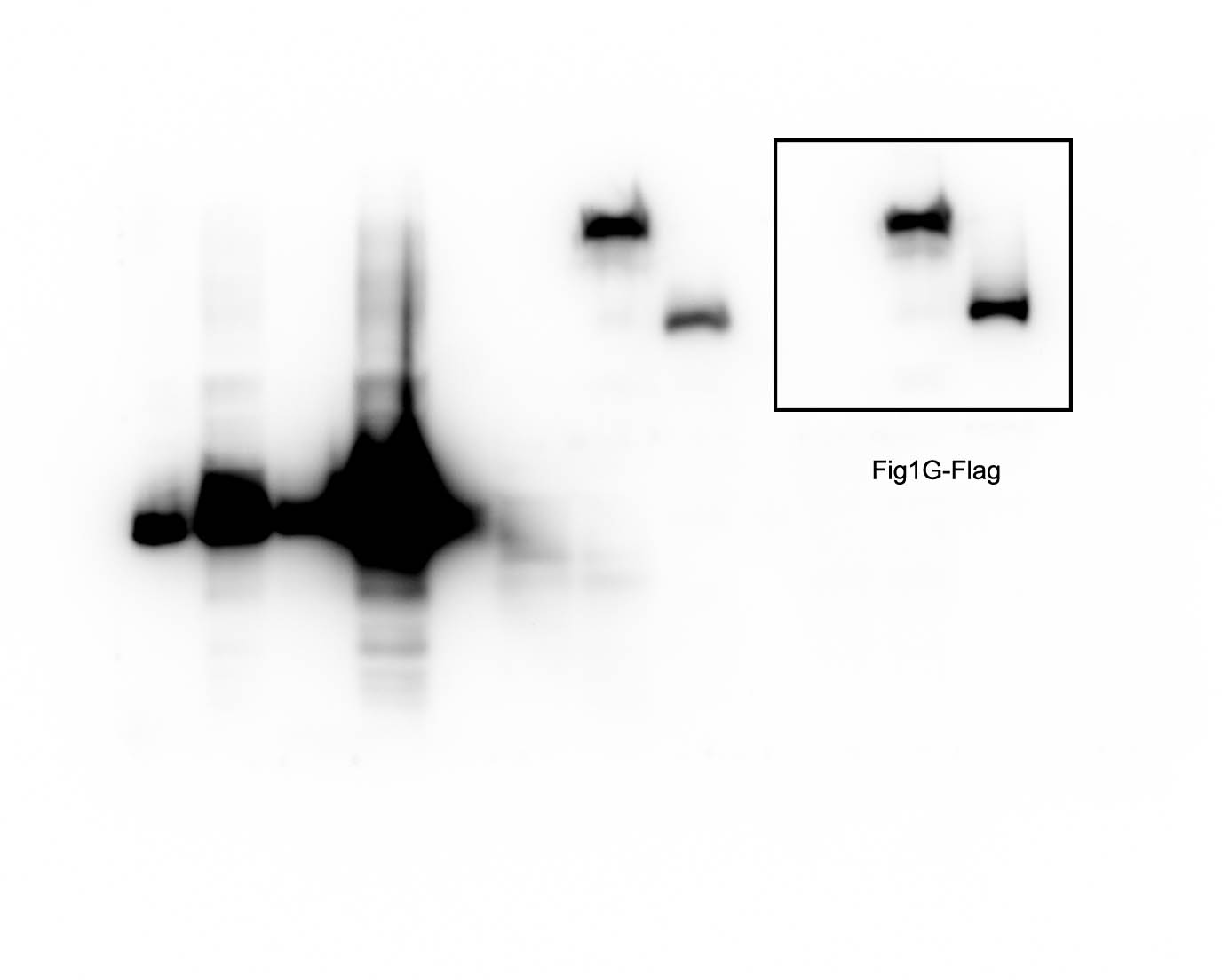

Supplement: Figure 1—source data 3. [file elife-94898-fig1-data3.zip › Fig1G-Flag.jpg]

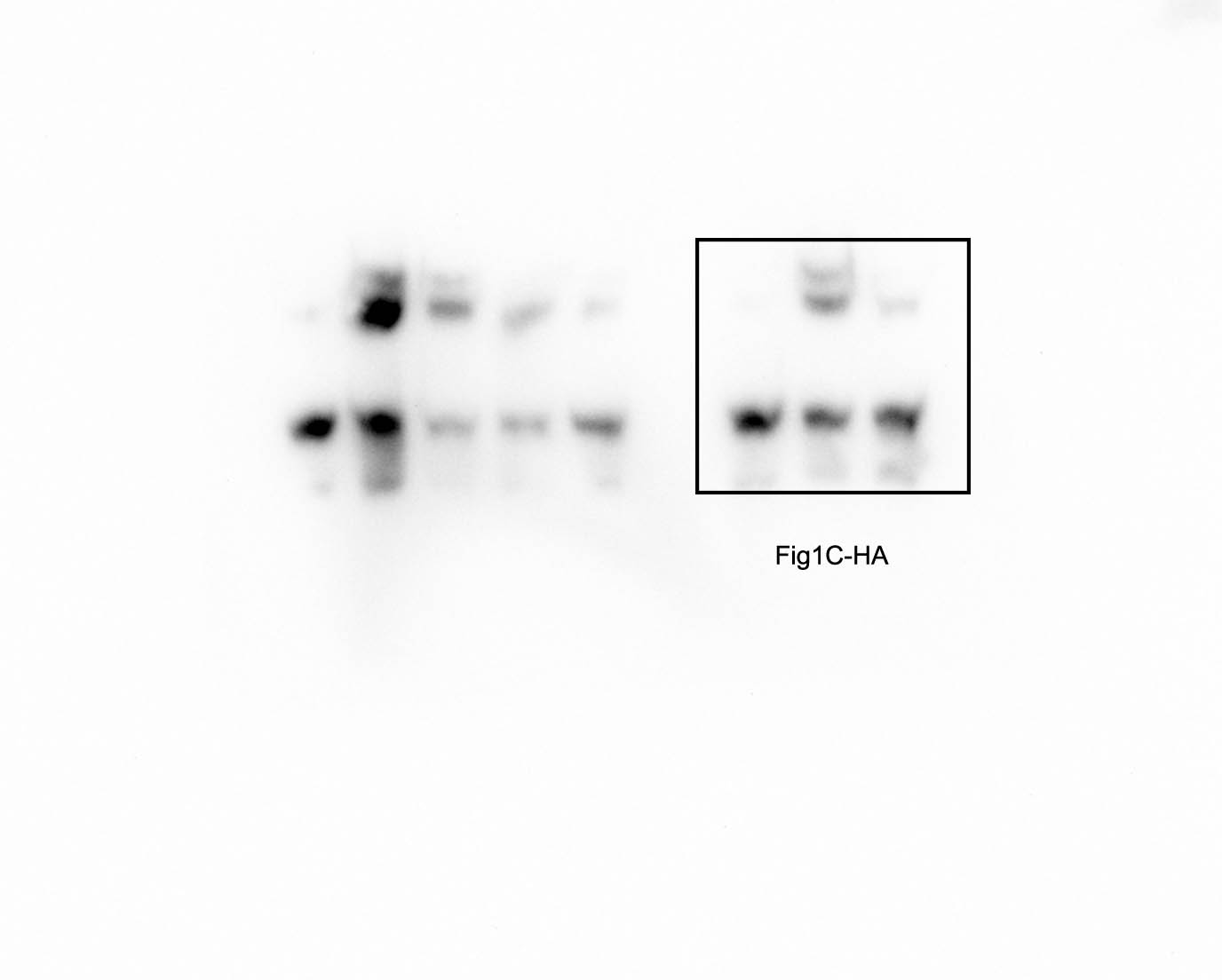

Supplement: Figure 1—source data 3. [file elife-94898-fig1-data3.zip › Fig1C-HA.jpg]

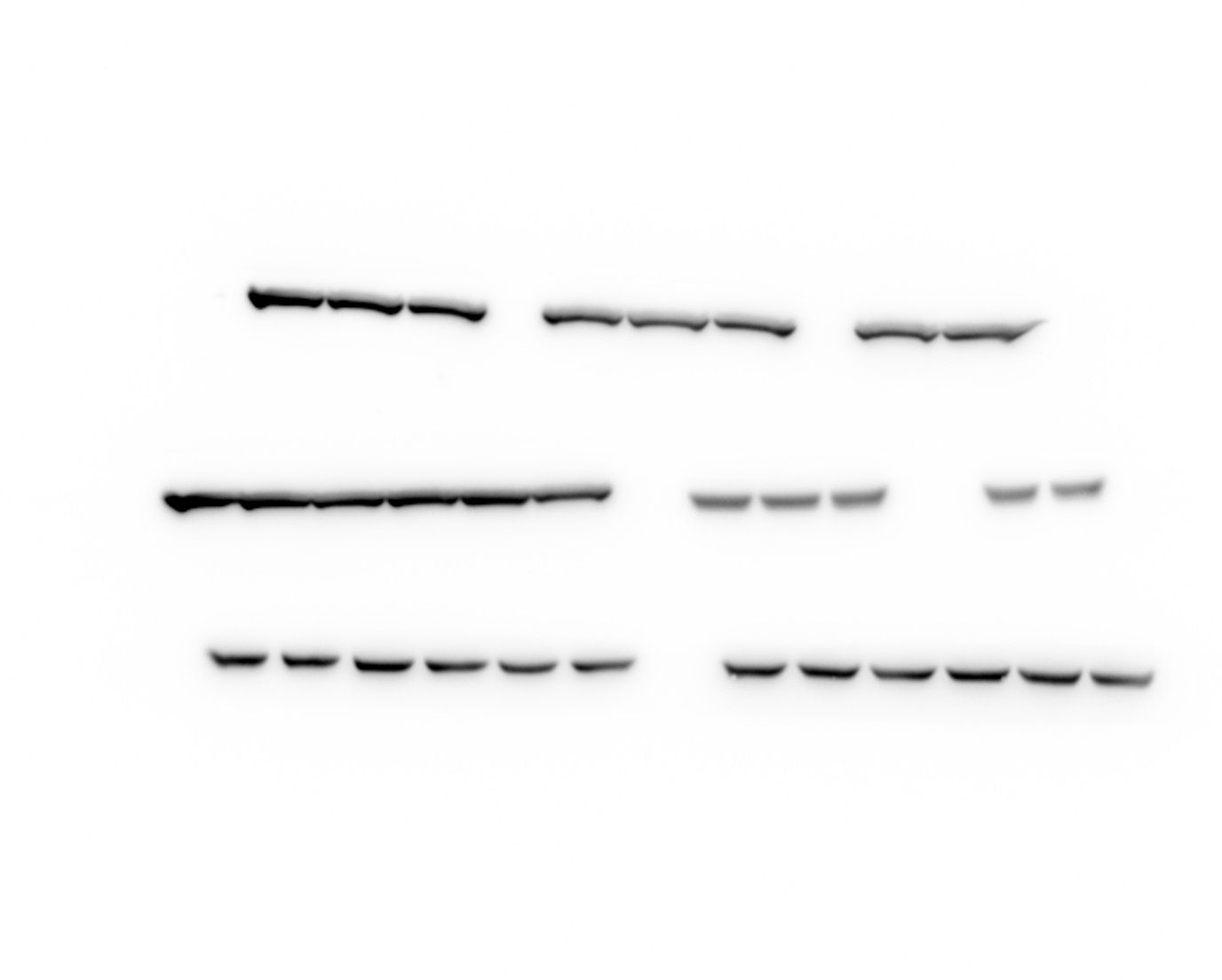

Supplement: Figure 2—source data 2. [file elife-94898-fig2-data2.zip › Fig2A-Tubulin.Tif]

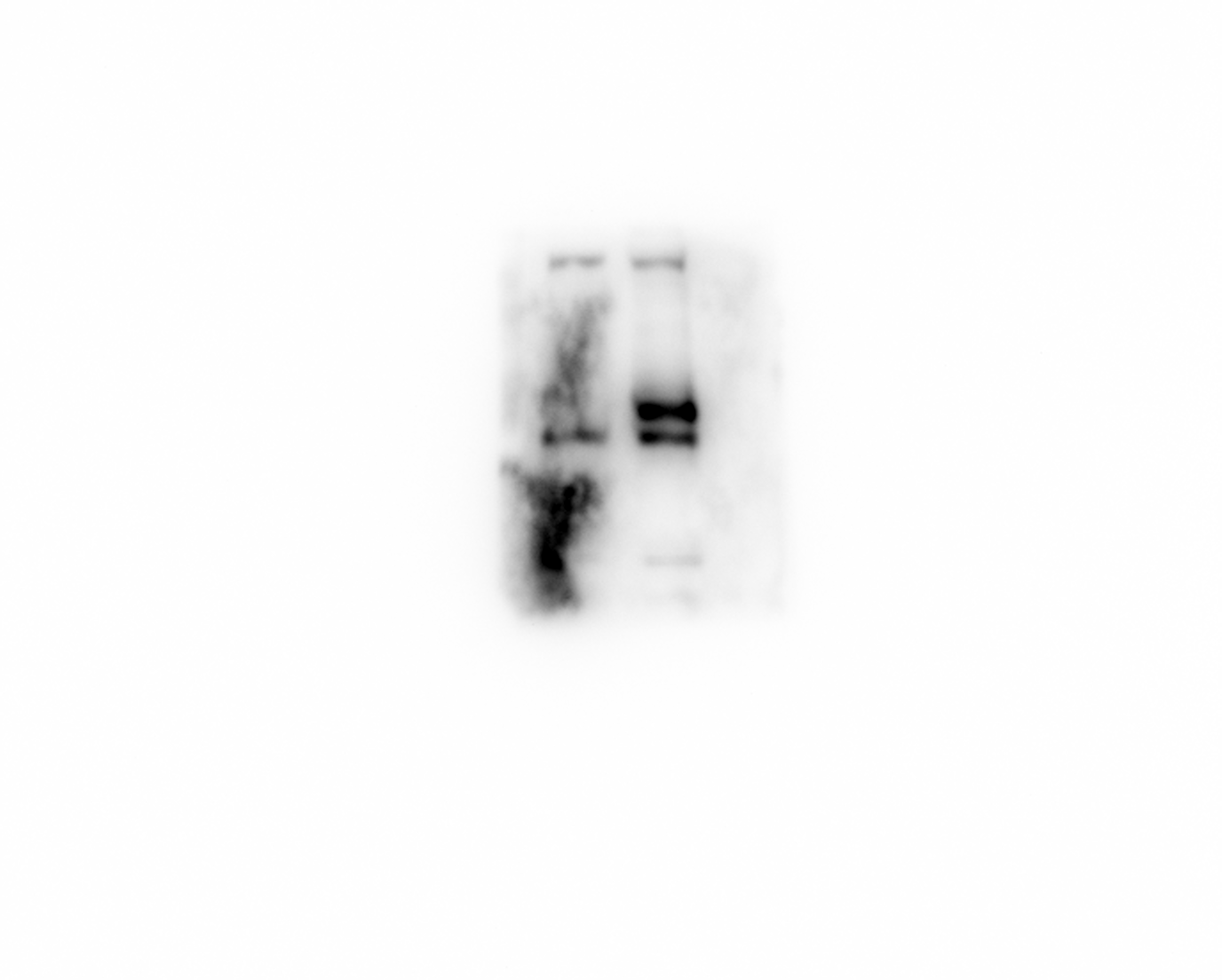

Supplement: Figure 2—source data 2. [file elife-94898-fig2-data2.zip › Fig2B-MDA5.Tif]

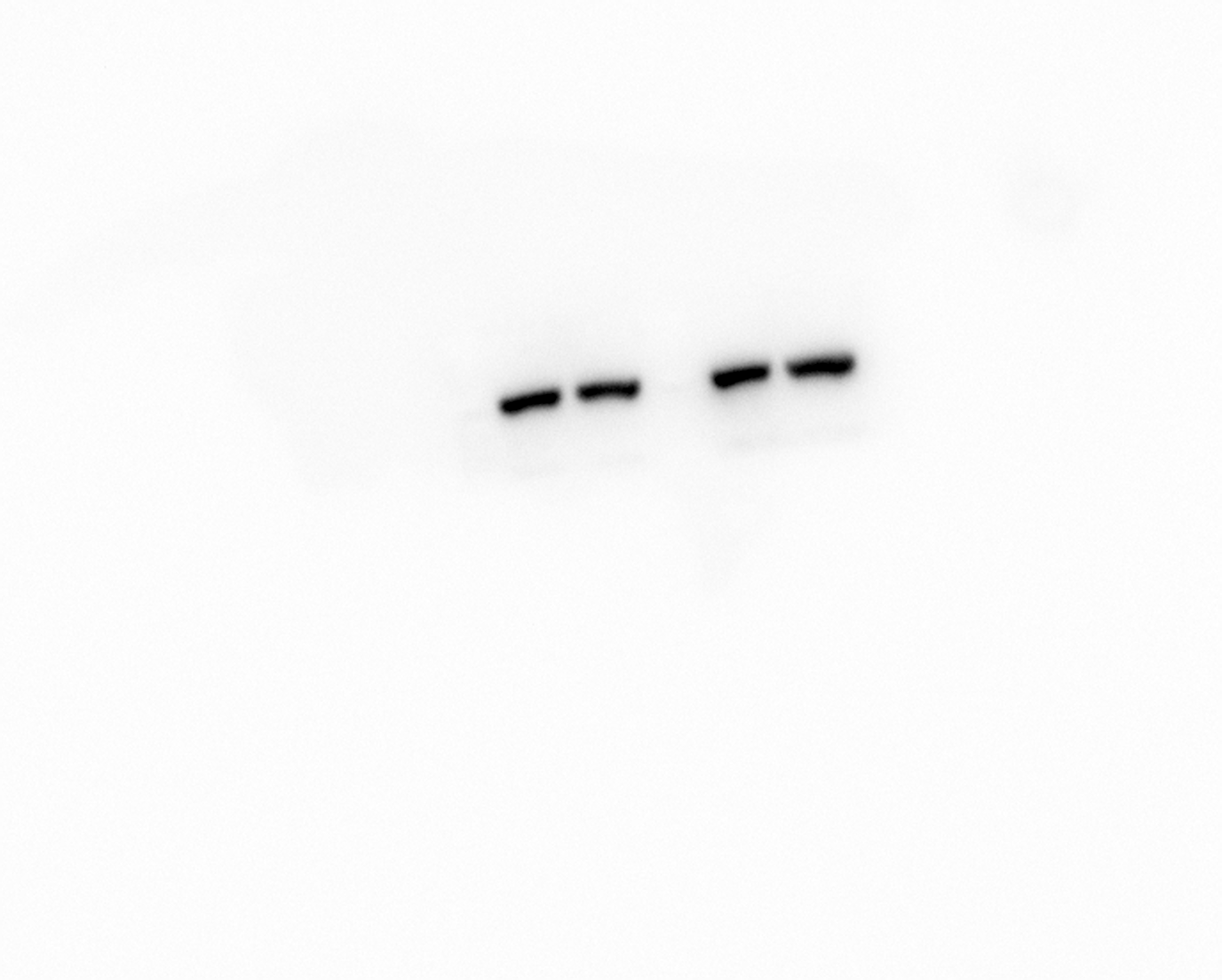

Supplement: Figure 2—source data 2. [file elife-94898-fig2-data2.zip › Fig2B-Tubulin.Tif]

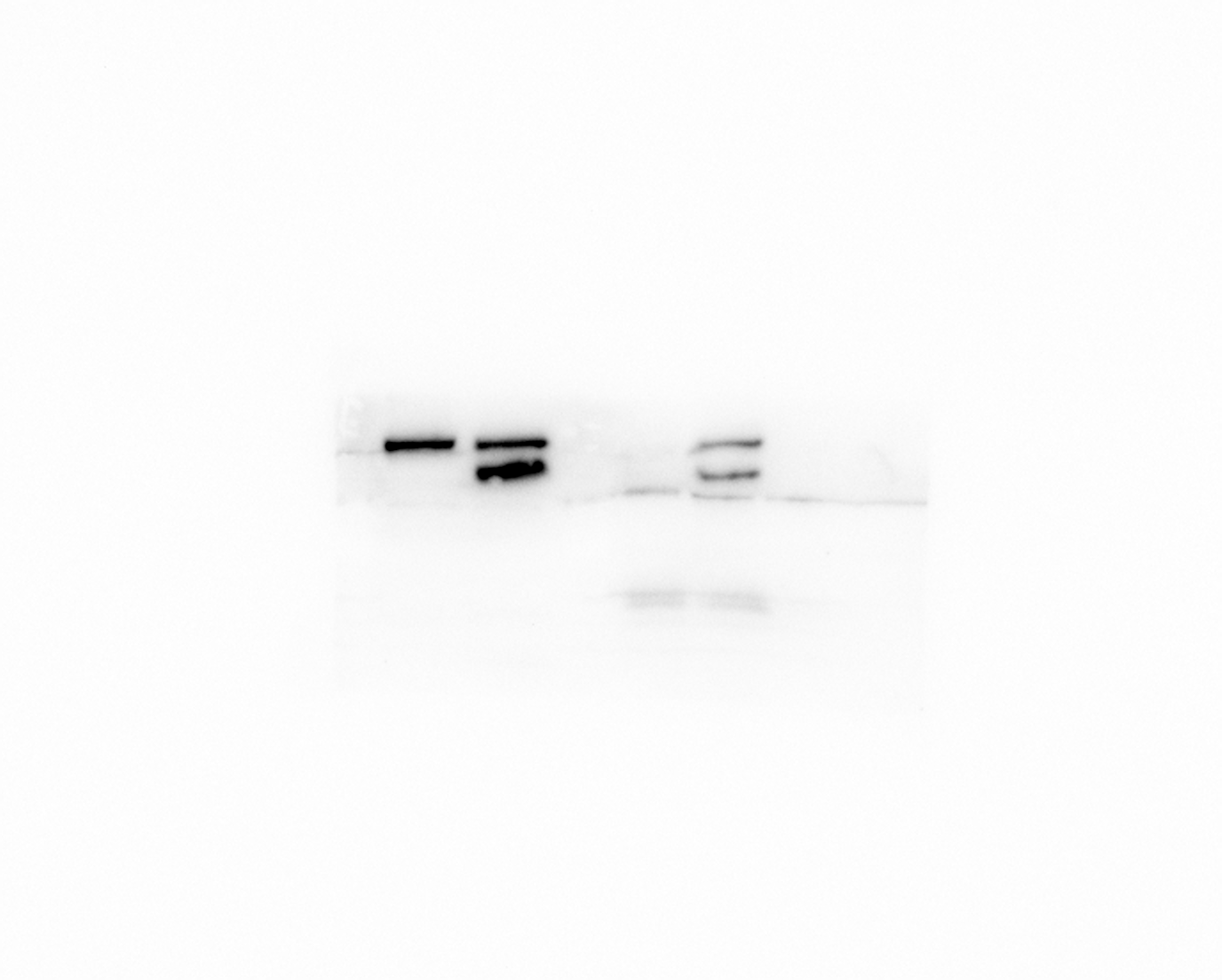

Supplement: Figure 2—source data 2. [file elife-94898-fig2-data2.zip › Fig2L.Tif]

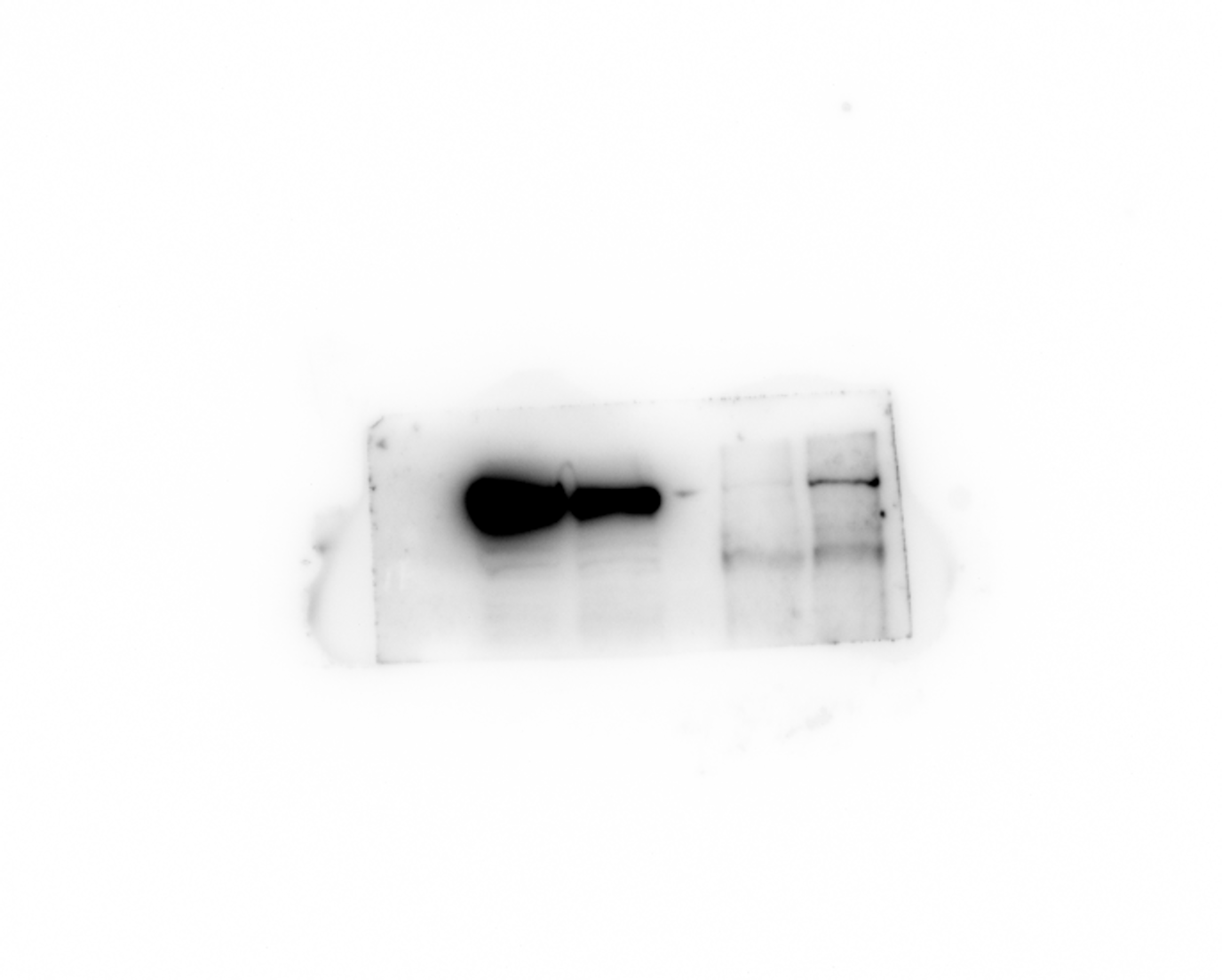

Supplement: Figure 2—source data 2. [file elife-94898-fig2-data2.zip › Fig2M-MDA5-Flag.Tif]

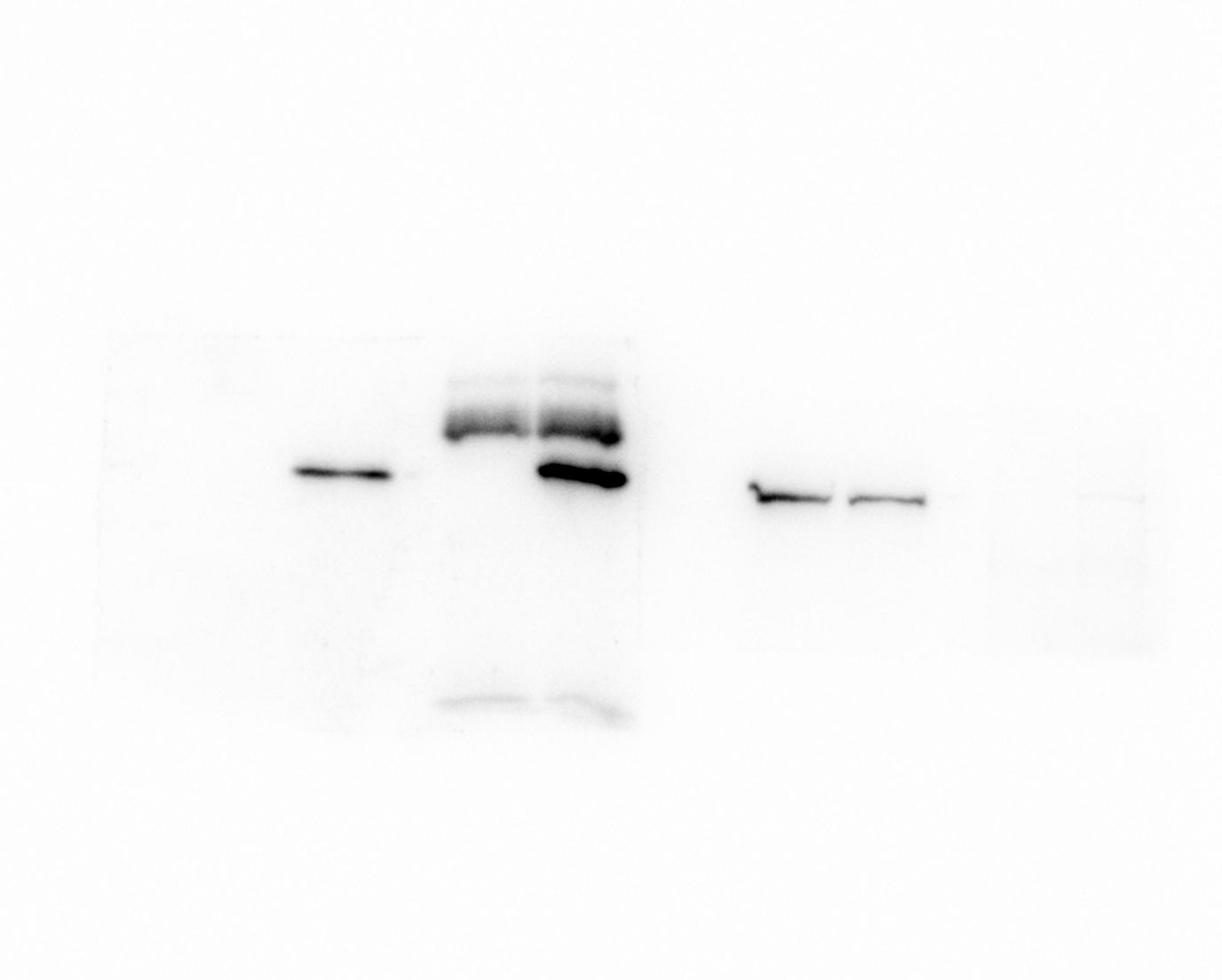

Supplement: Figure 2—source data 2. [file elife-94898-fig2-data2.zip › Fig2M-STING-Myc.Tif]

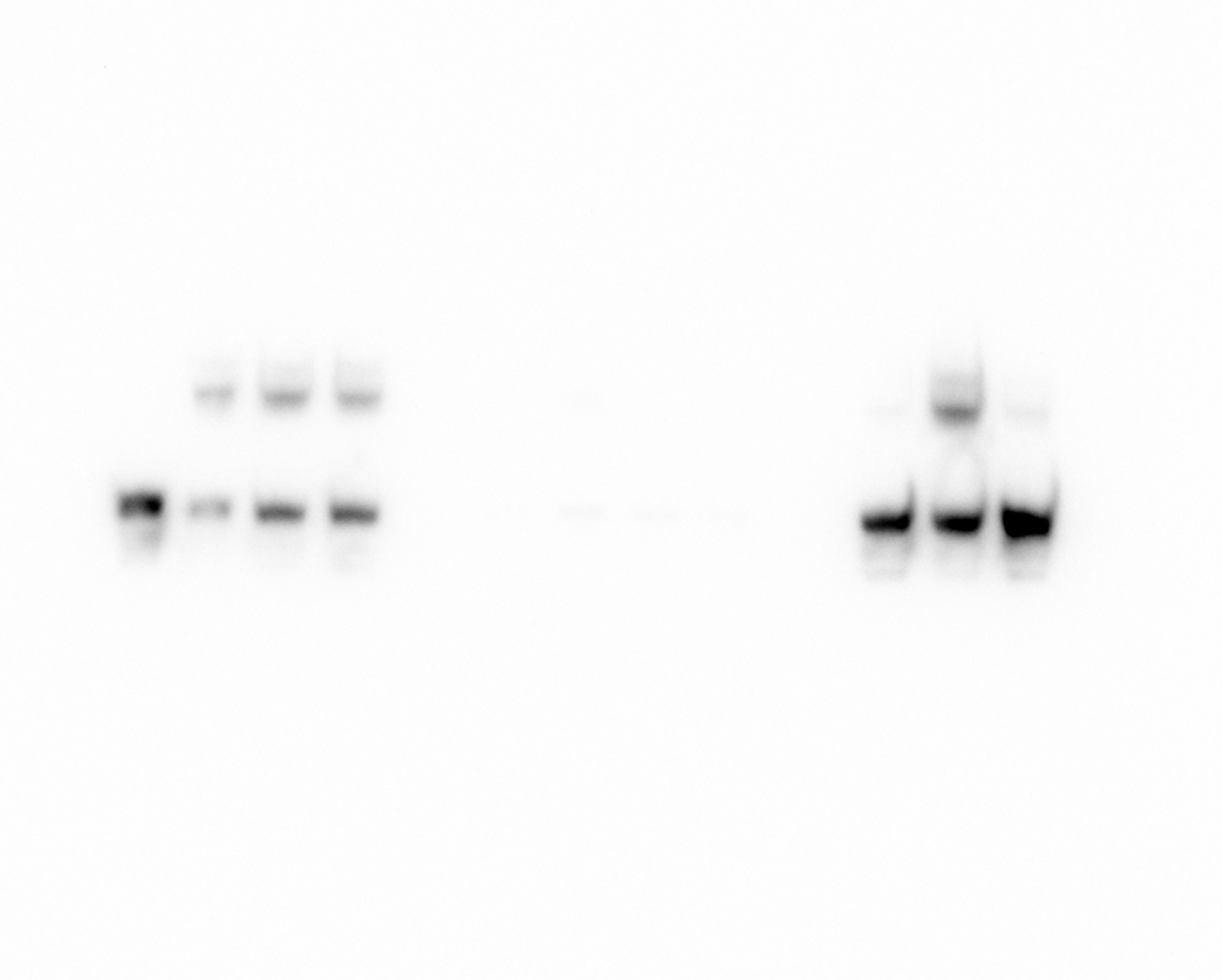

Supplement: Figure 2—source data 2. [file elife-94898-fig2-data2.zip › Fig2P- HA.Tif]

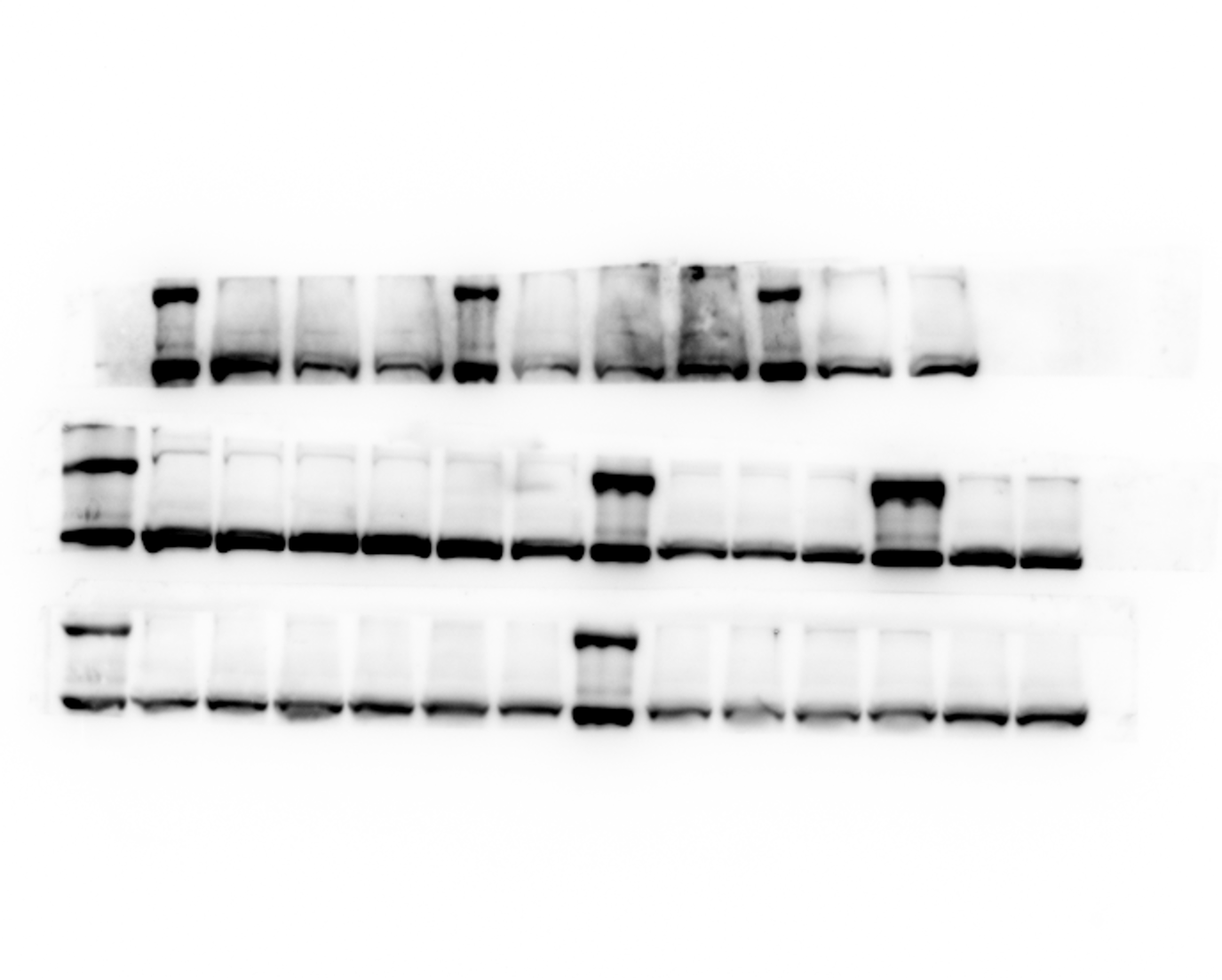

Supplement: Figure 2—source data 2. [file elife-94898-fig2-data2.zip › Fig2A-MDA5.Tif]

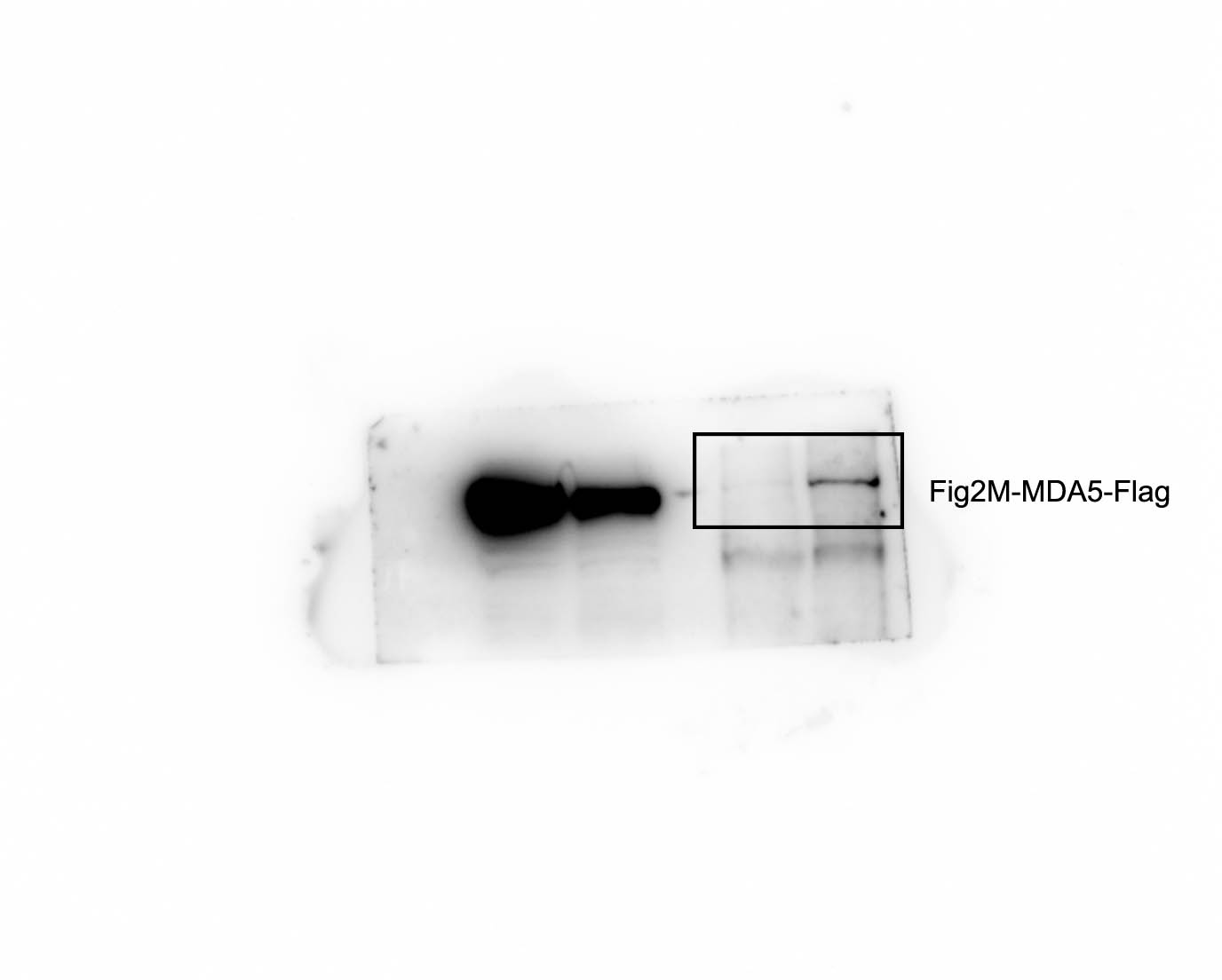

Supplement: Figure 2—source data 3. [file elife-94898-fig2-data3.zip › Fig2M-MDA5-Flag.jpg]

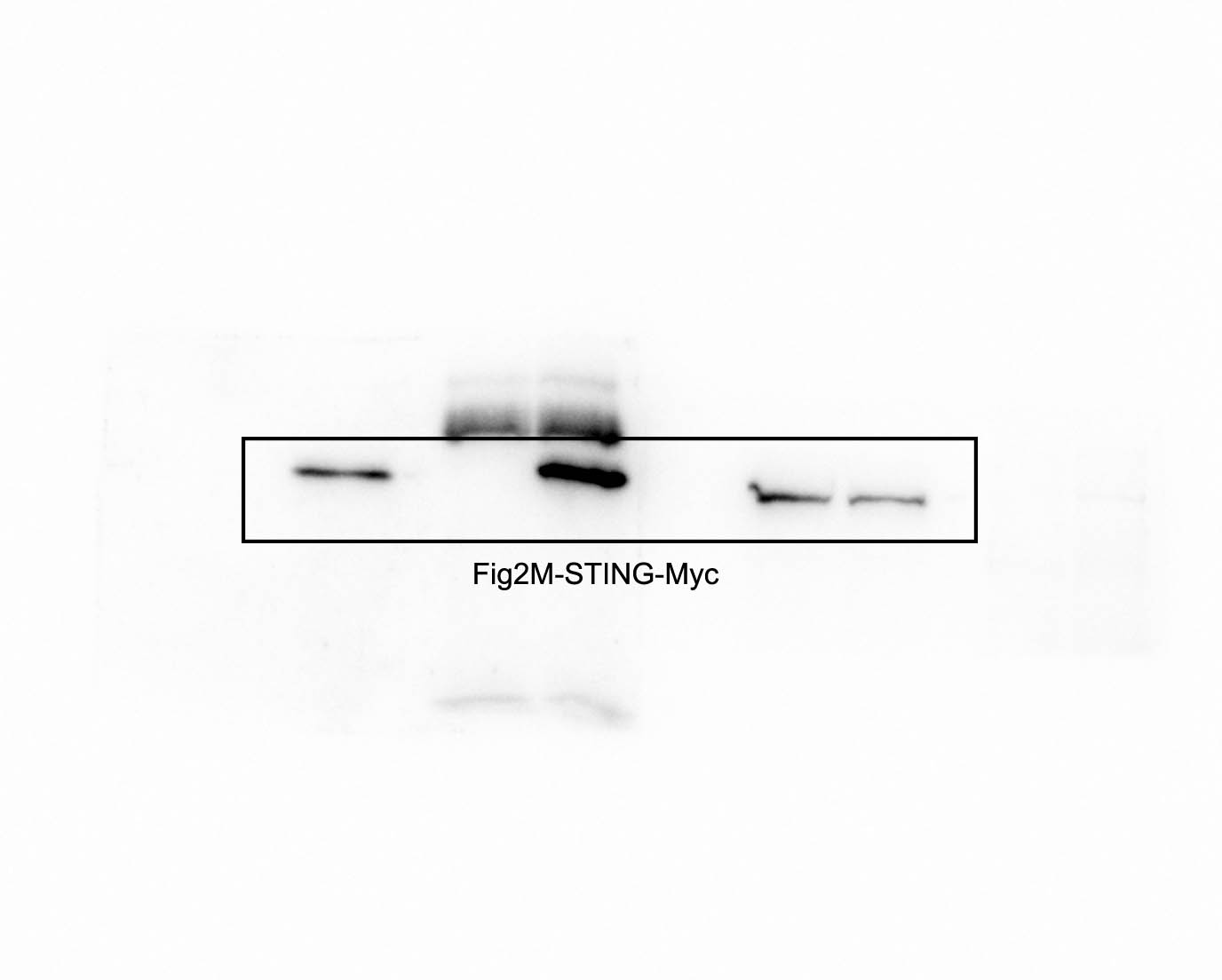

Supplement: Figure 2—source data 3. [file elife-94898-fig2-data3.zip › Fig2M-STING-Myc.jpg]

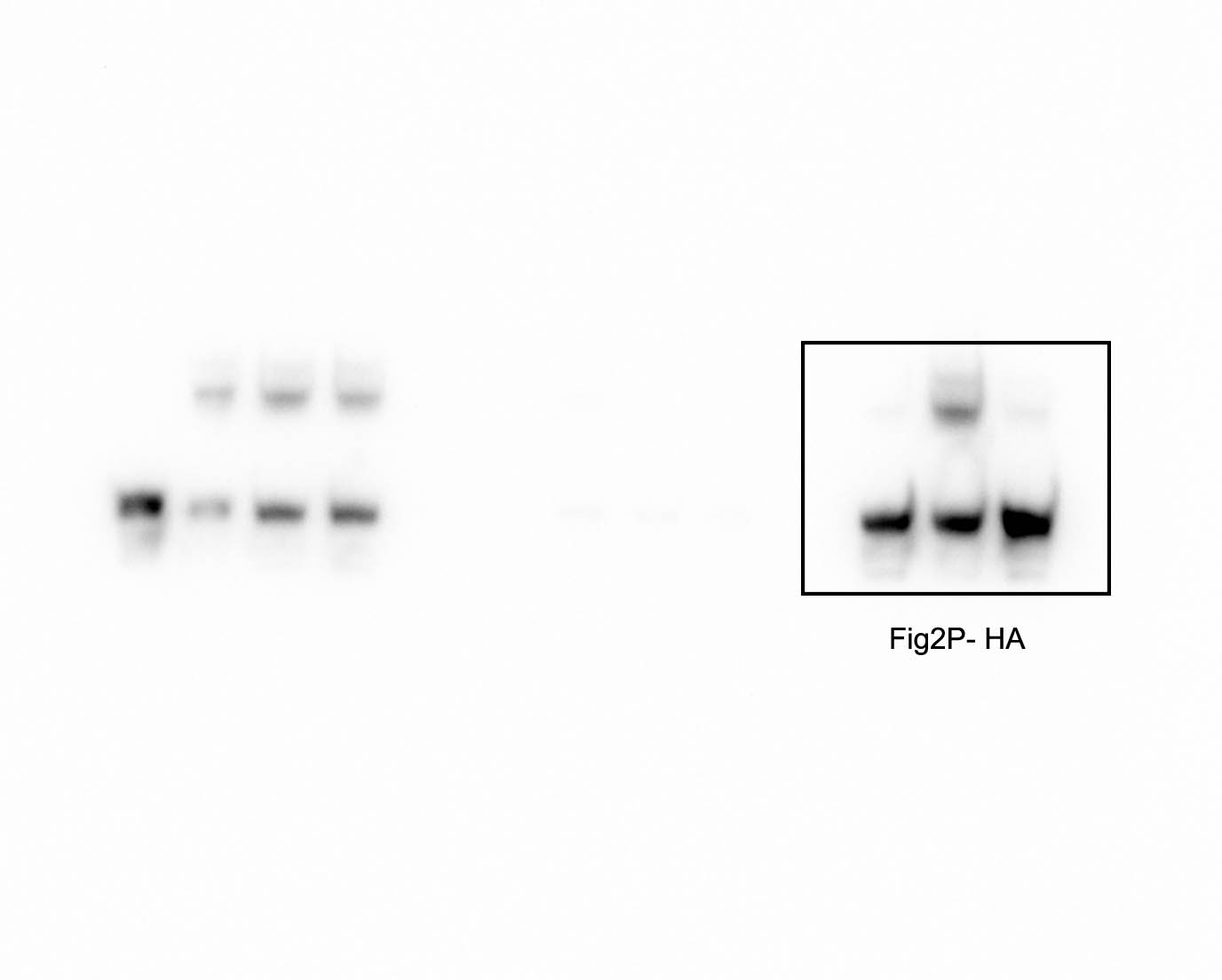

Supplement: Figure 2—source data 3. [file elife-94898-fig2-data3.zip › Fig2P-HA.jpg]

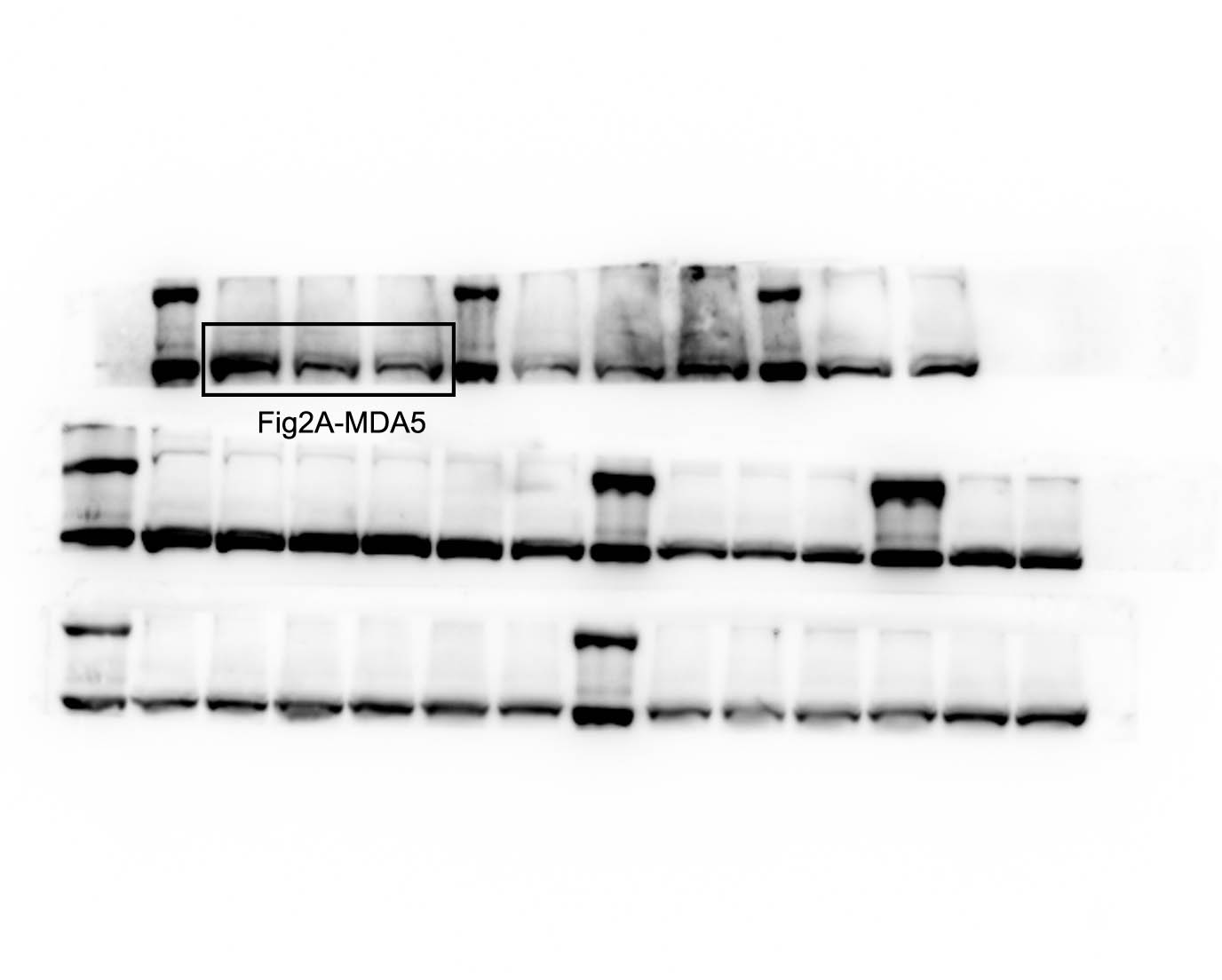

Supplement: Figure 2—source data 3. [file elife-94898-fig2-data3.zip › Fig2A-MDA5.jpg]

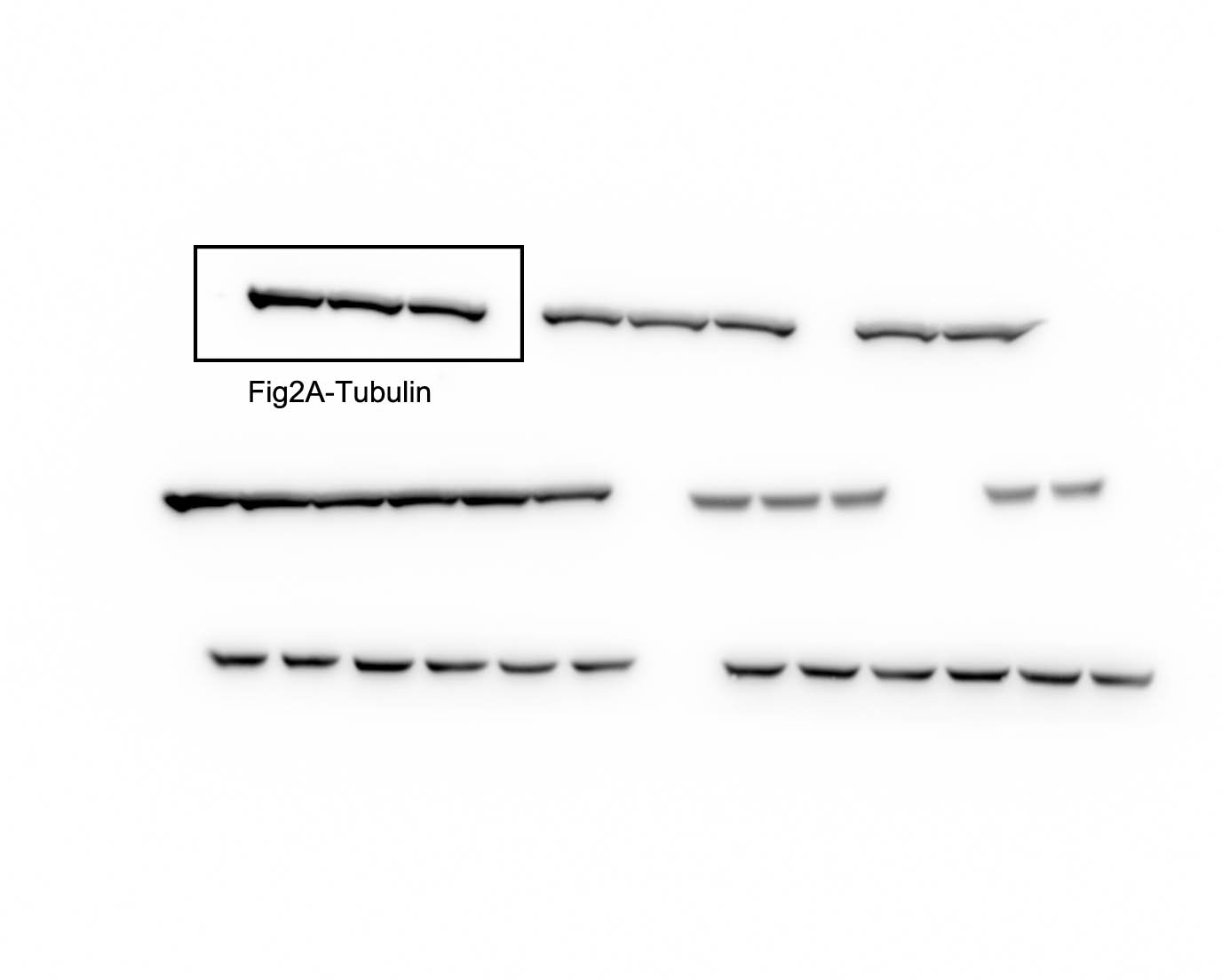

Supplement: Figure 2—source data 3. [file elife-94898-fig2-data3.zip › Fig2A-Tubulin.jpg]

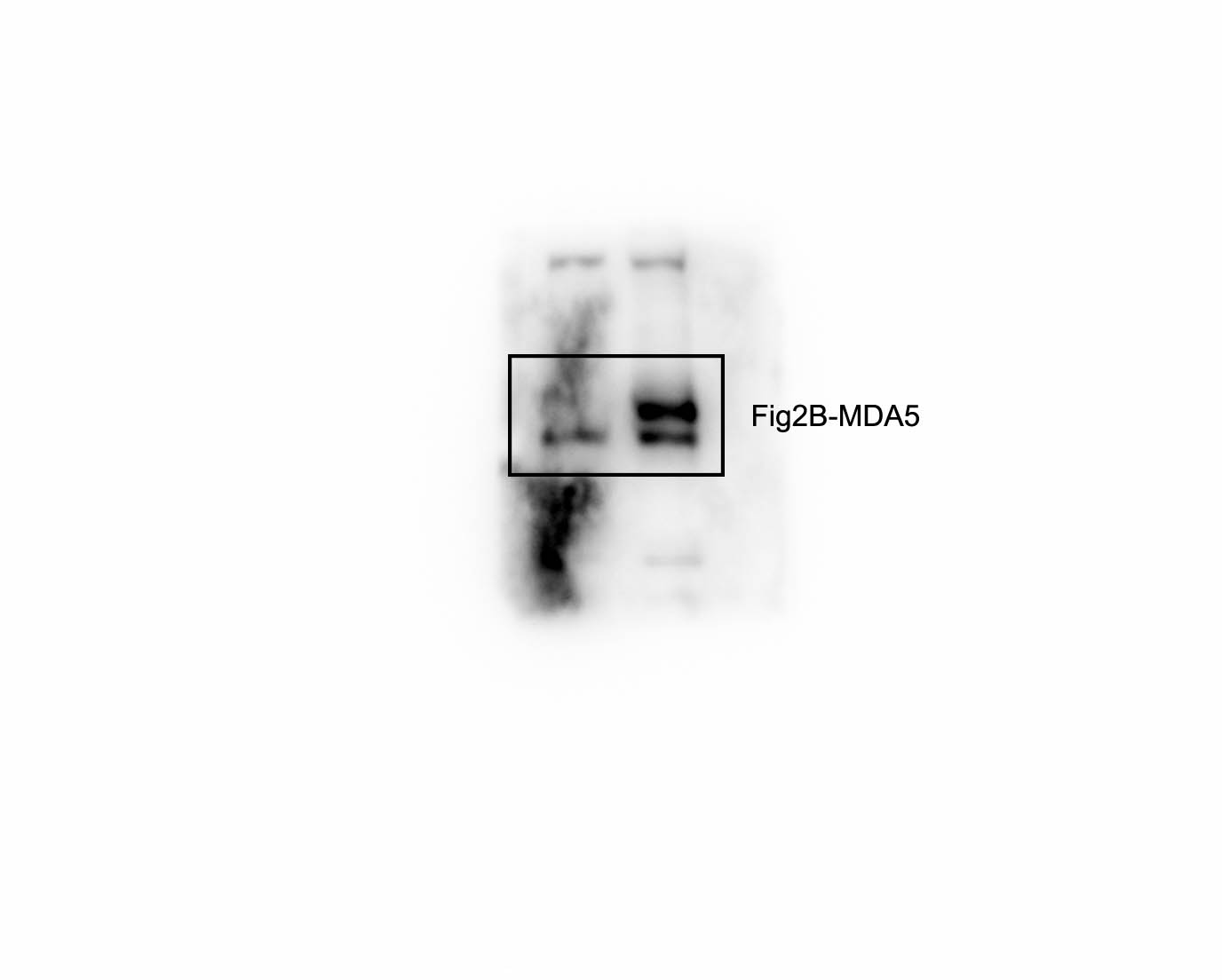

Supplement: Figure 2—source data 3. [file elife-94898-fig2-data3.zip › Fig2B-MDA5.jpg]

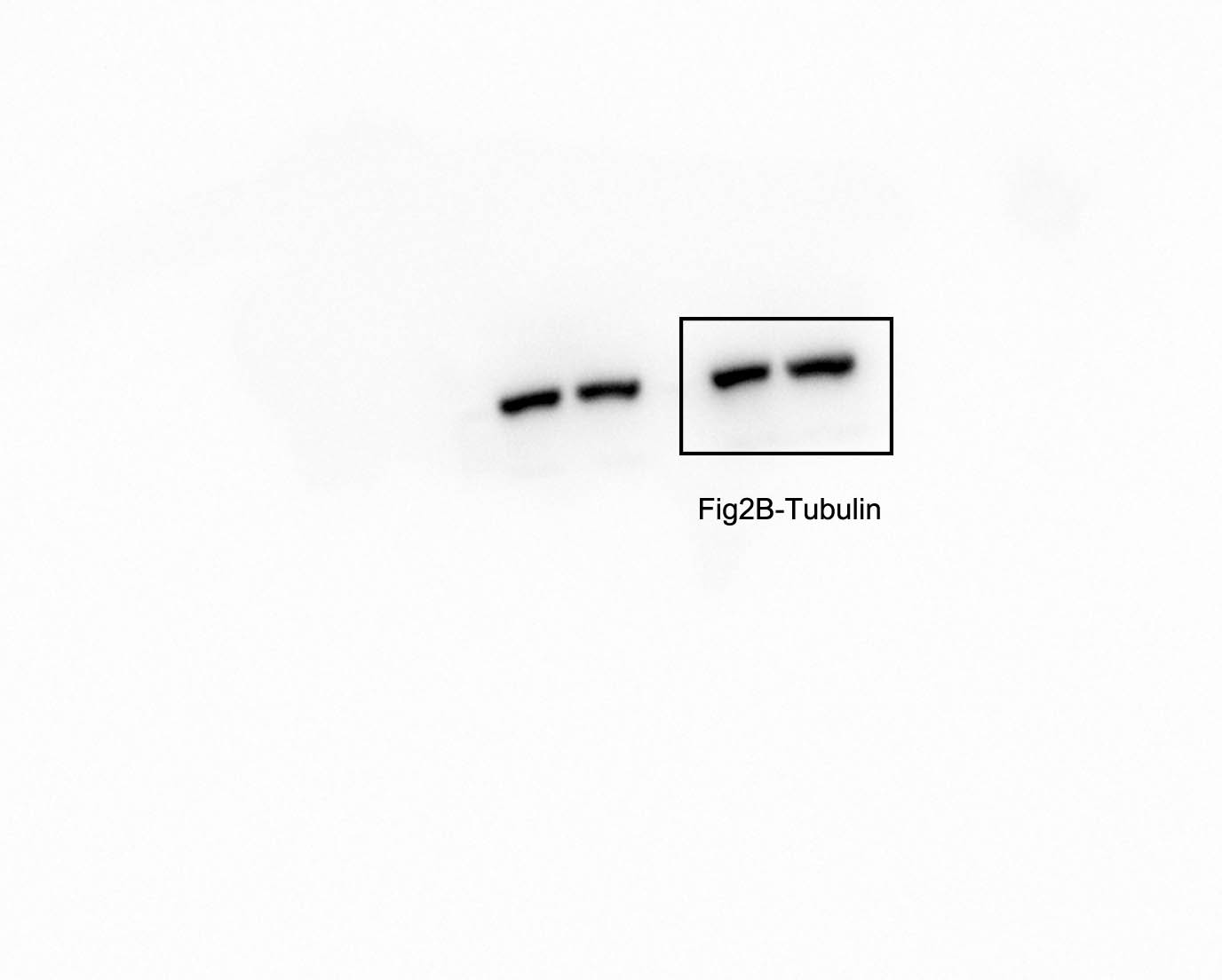

Supplement: Figure 2—source data 3. [file elife-94898-fig2-data3.zip › Fig2B-Tubulin.jpg]

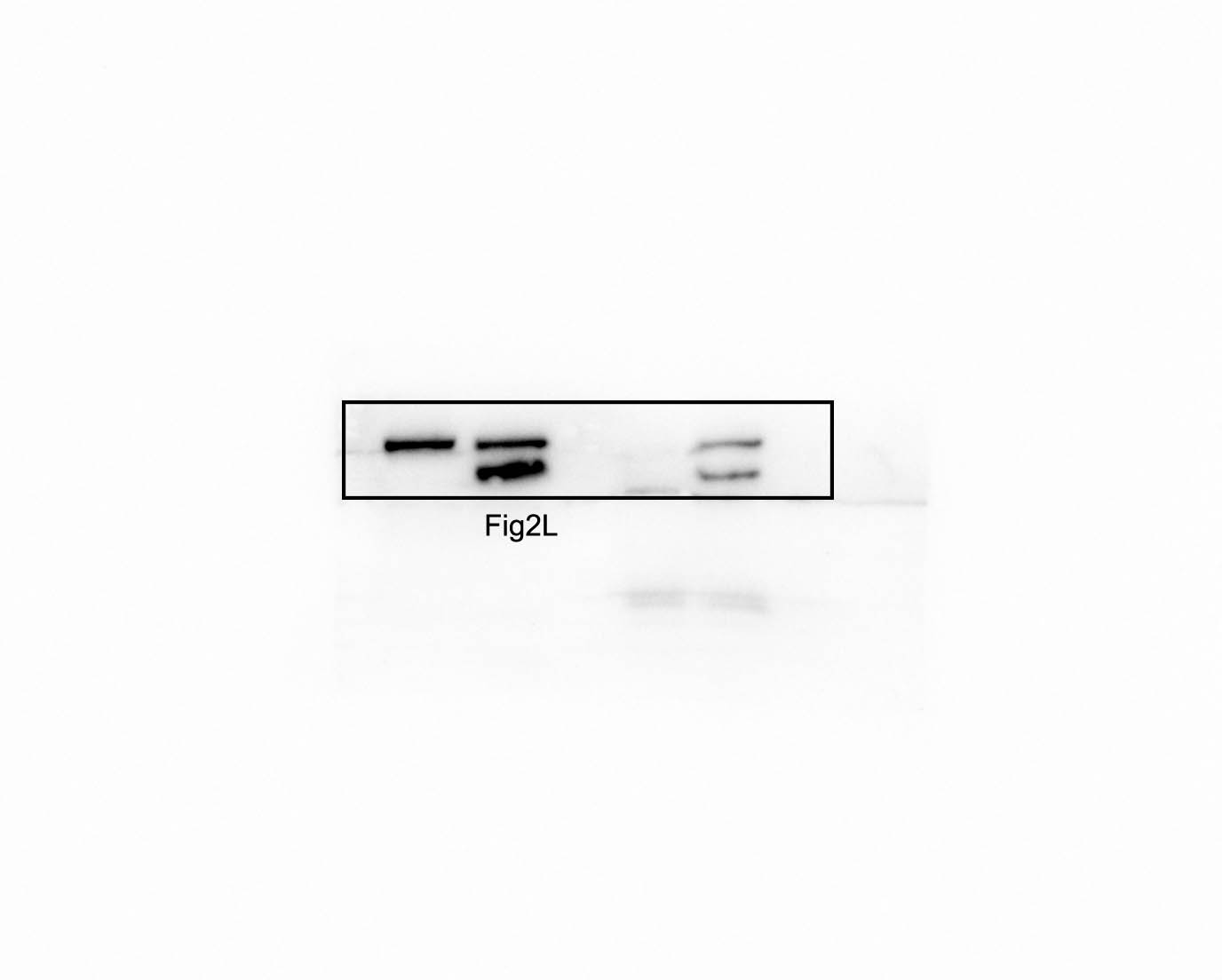

Supplement: Figure 2—source data 3. [file elife-94898-fig2-data3.zip › Fig2L.jpg]

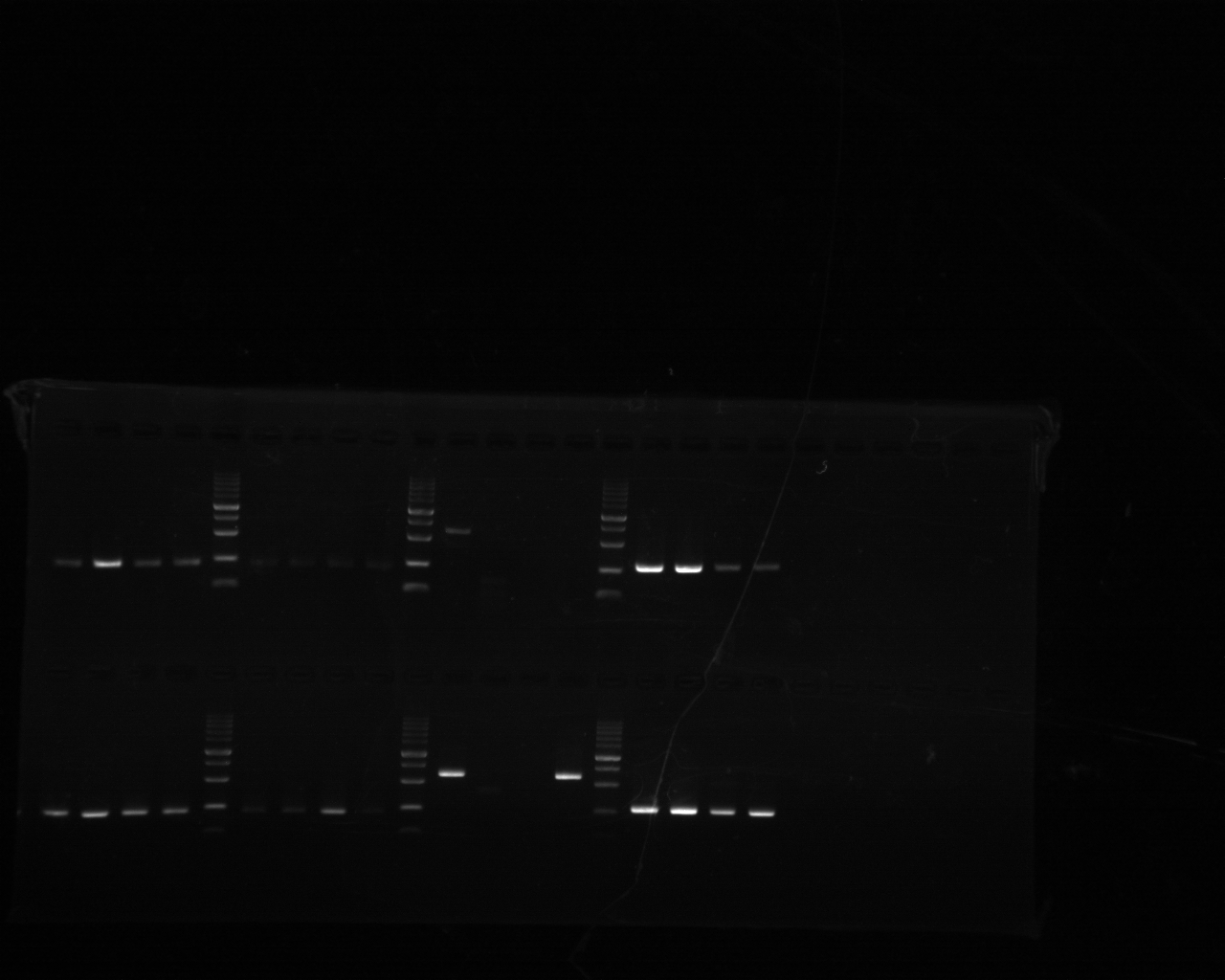

Supplement: Figure 3—source data 2. [file elife-94898-fig3-data2.zip › Fig3A-β-actin.tif]

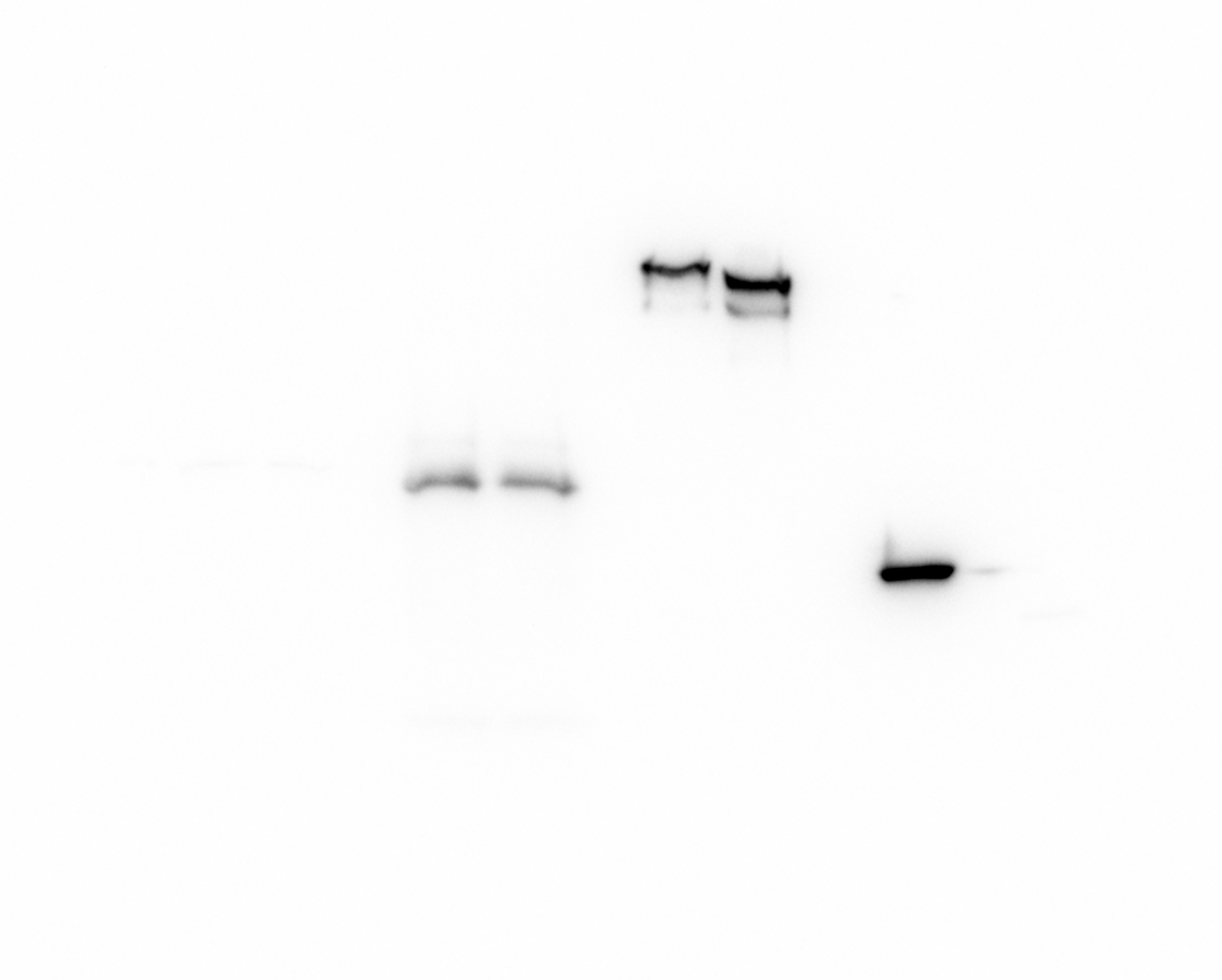

Supplement: Figure 3—source data 2. [file elife-94898-fig3-data2.zip › Fig3B-MDA5-Flag.Tif]

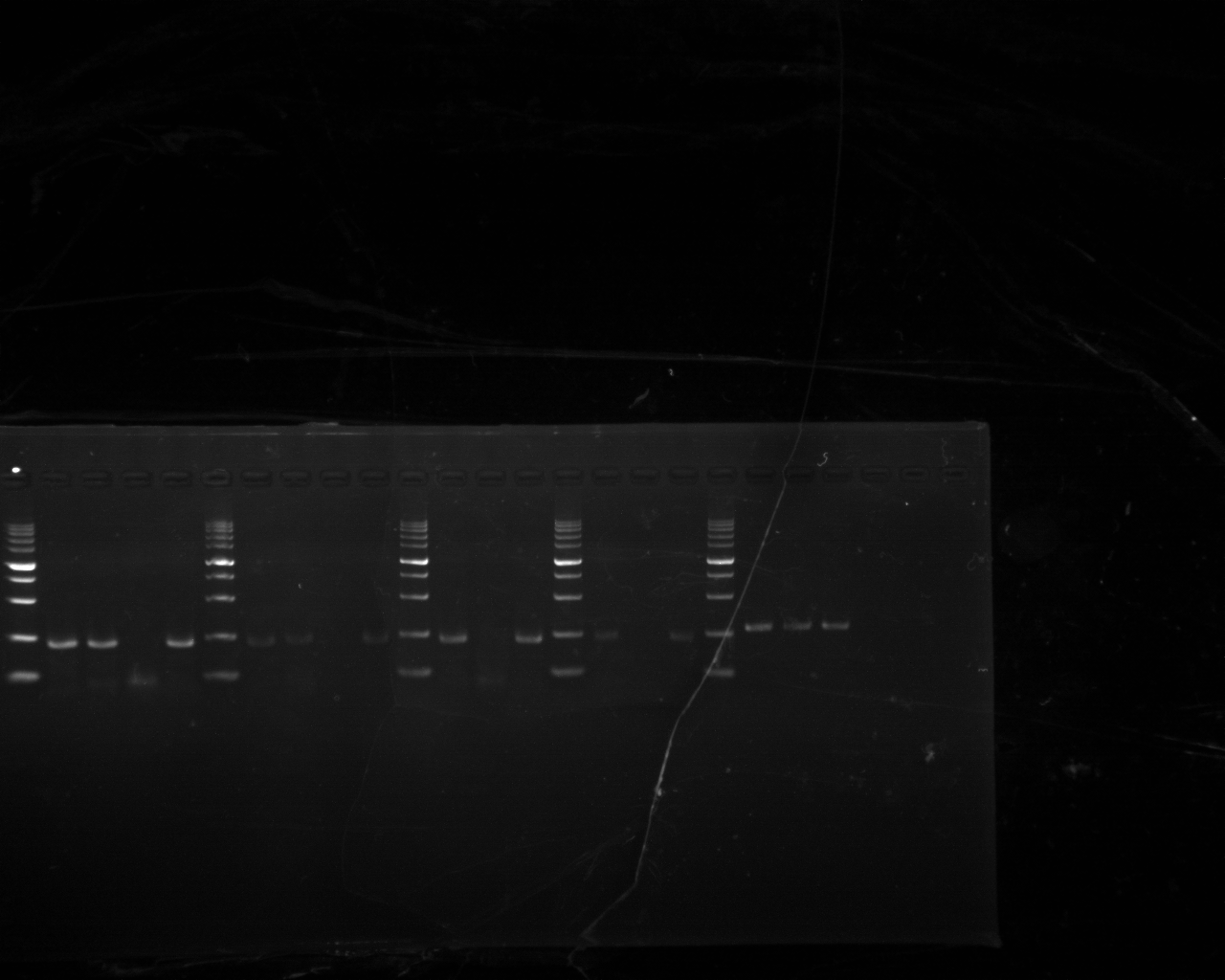

Supplement: Figure 3—source data 2. [file elife-94898-fig3-data2.zip › Fig3A-SCRV-M&G.tif]

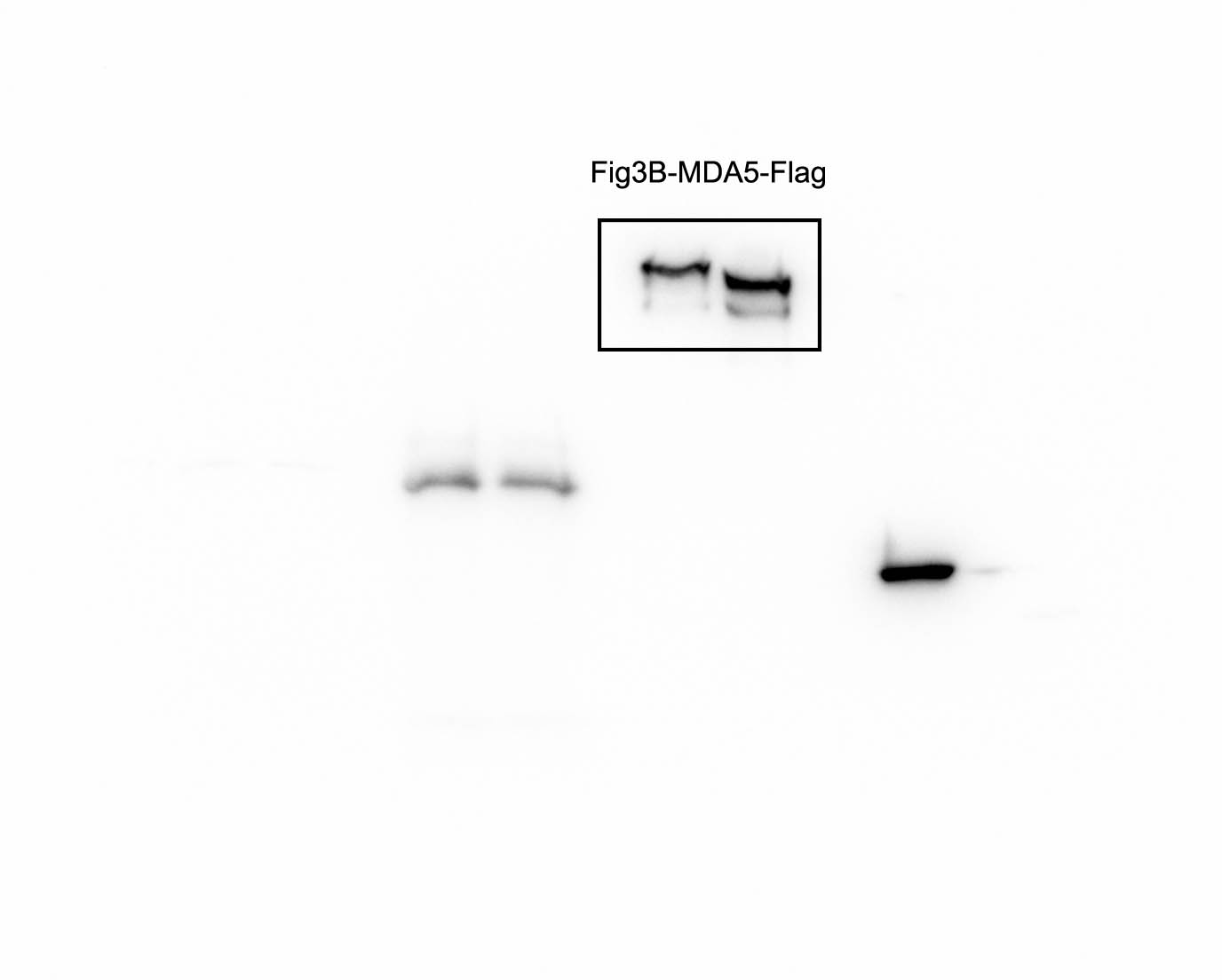

Supplement: Figure 3—source data 3. [file elife-94898-fig3-data3.zip › Fig3B-MDA5-Flag.jpg]

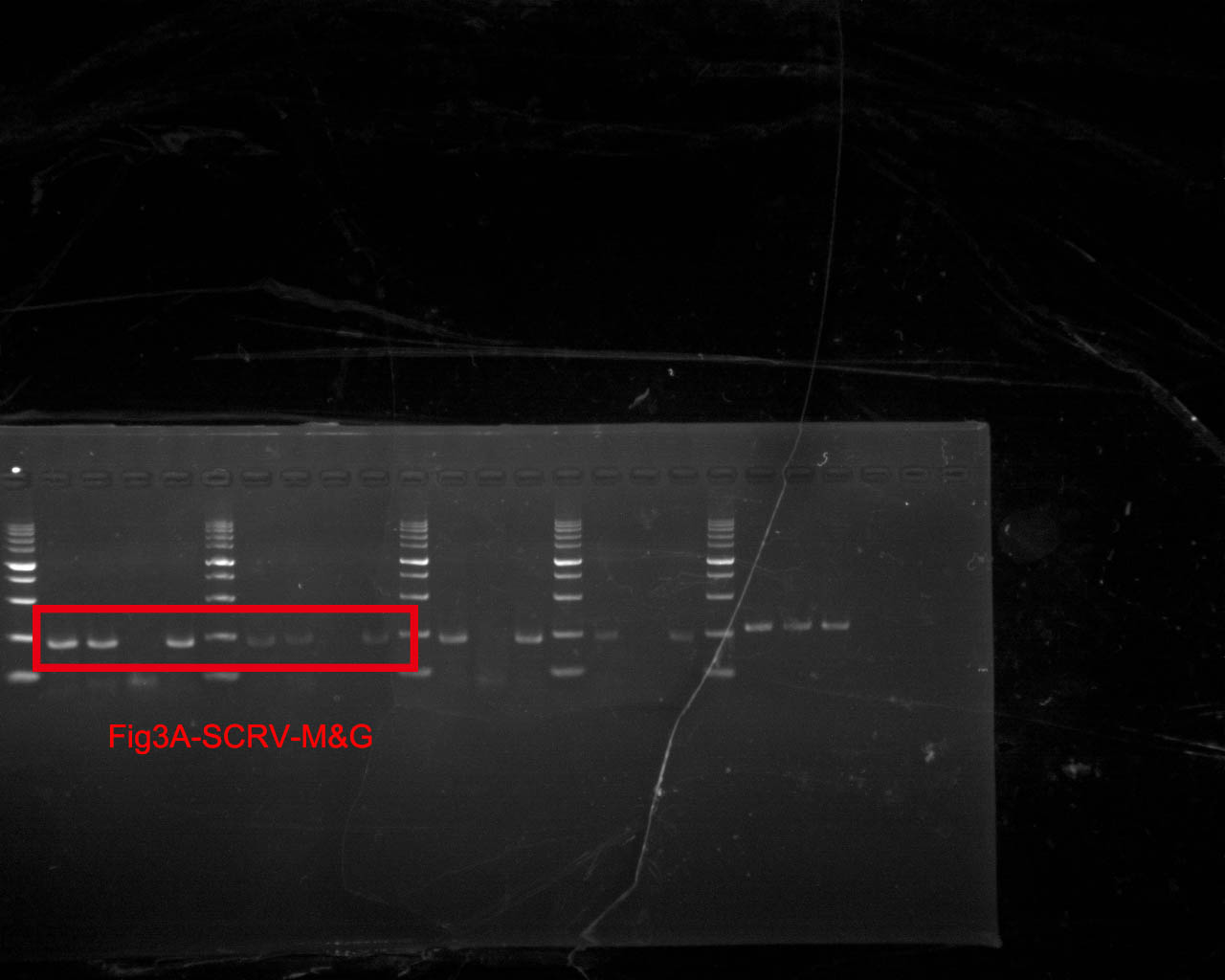

Supplement: Figure 3—source data 3. [file elife-94898-fig3-data3.zip › Fig3A-SCRV-M&G.jpg]

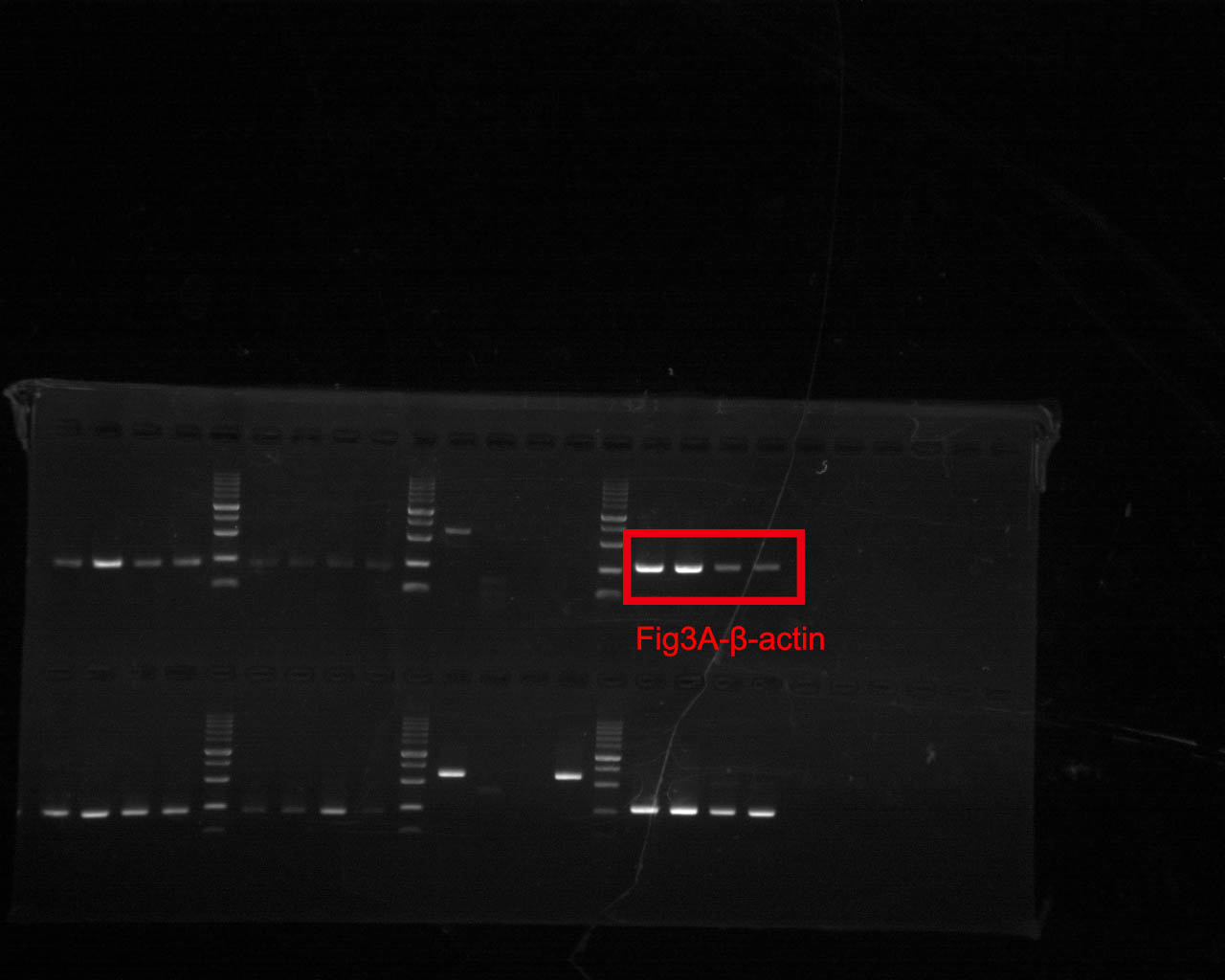

Supplement: Figure 3—source data 3. [file elife-94898-fig3-data3.zip › Fig3A-β-actin.jpg]

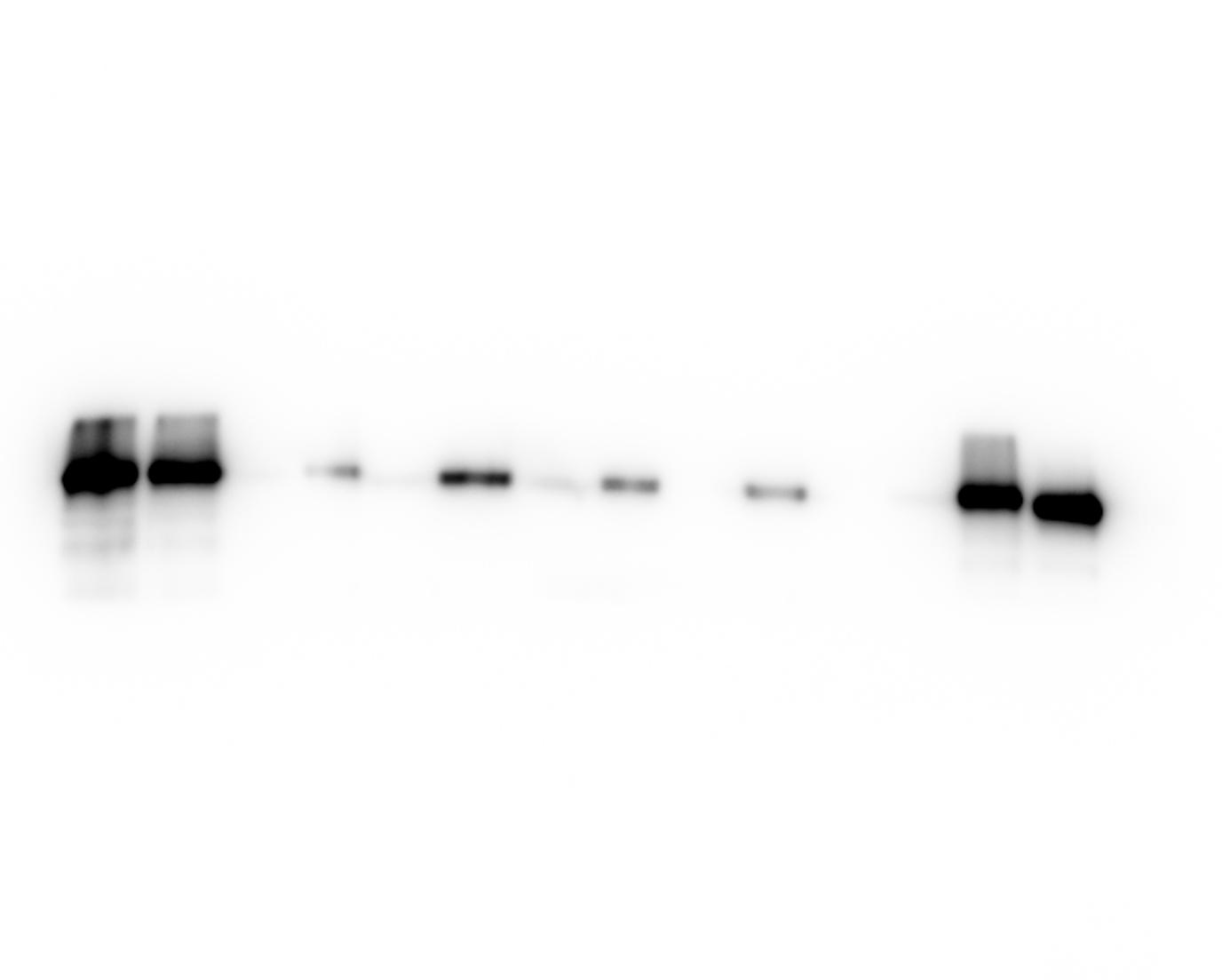

Supplement: Figure 4—source data 1. [file elife-94898-fig4-data1.zip › Fig4J-Flag.Tif]

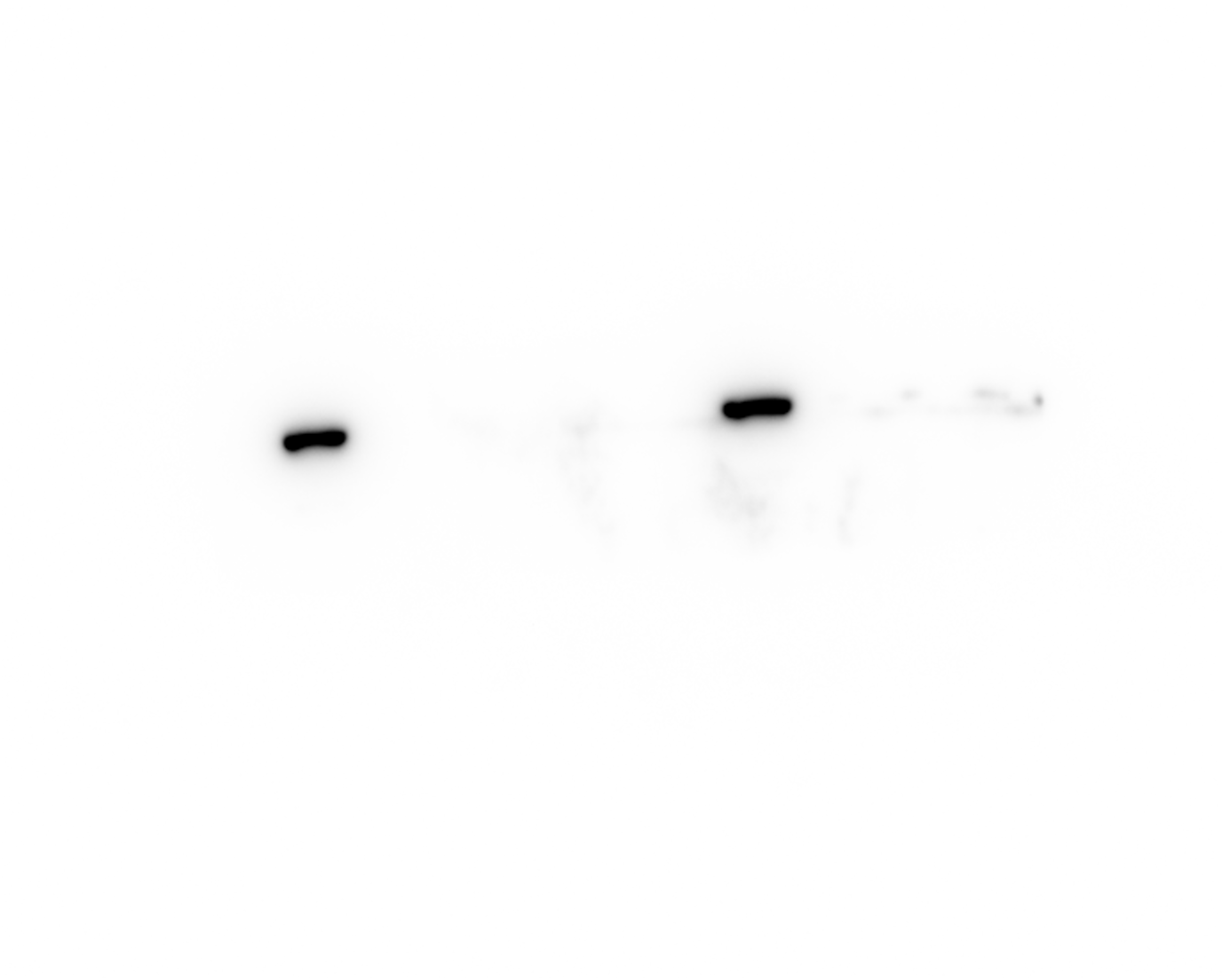

Supplement: Figure 4—source data 1. [file elife-94898-fig4-data1.zip › Fig4J-Tubulin.Tif]

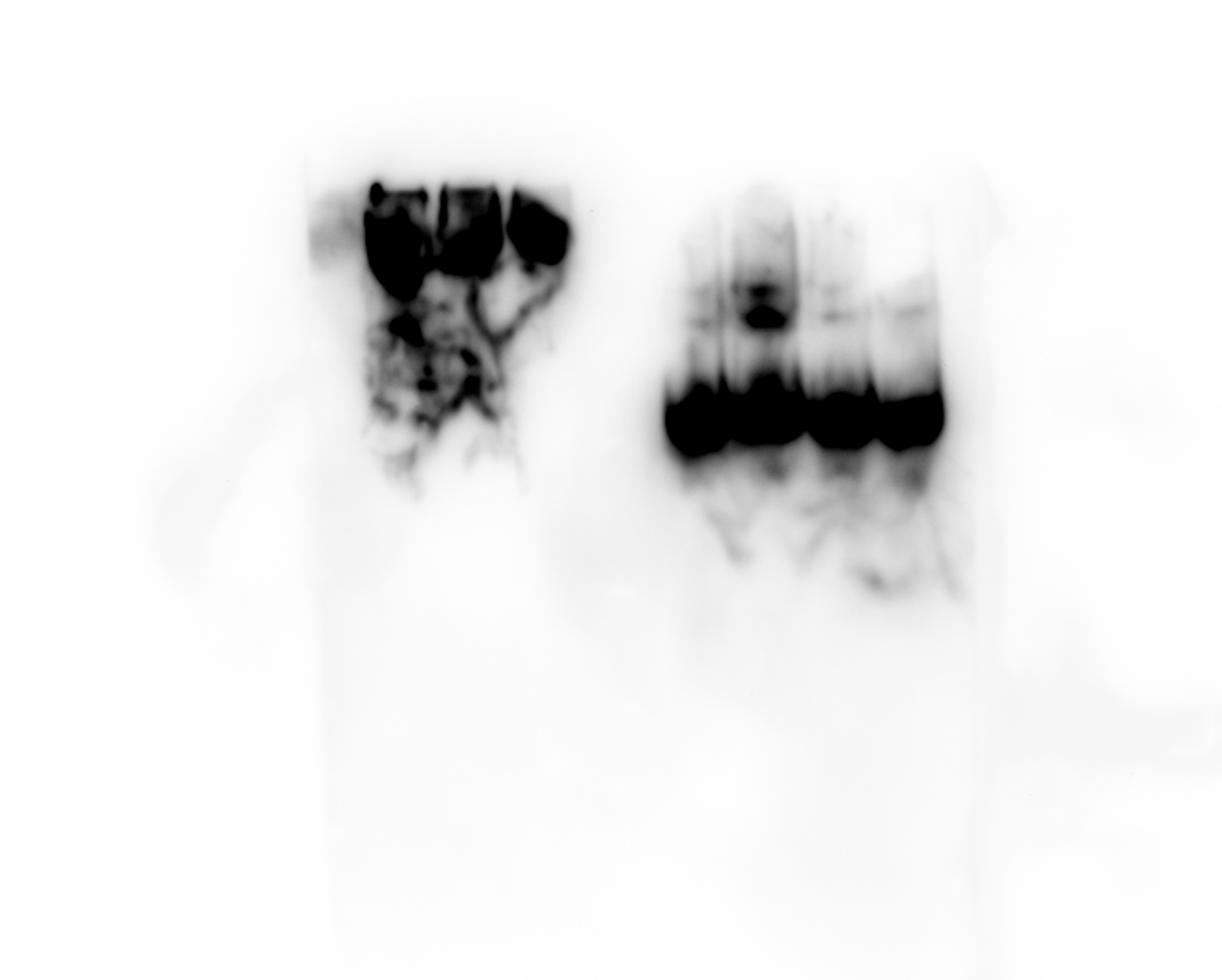

Supplement: Figure 4—source data 1. [file elife-94898-fig4-data1.zip › Fig4K-IRF3.Tif]

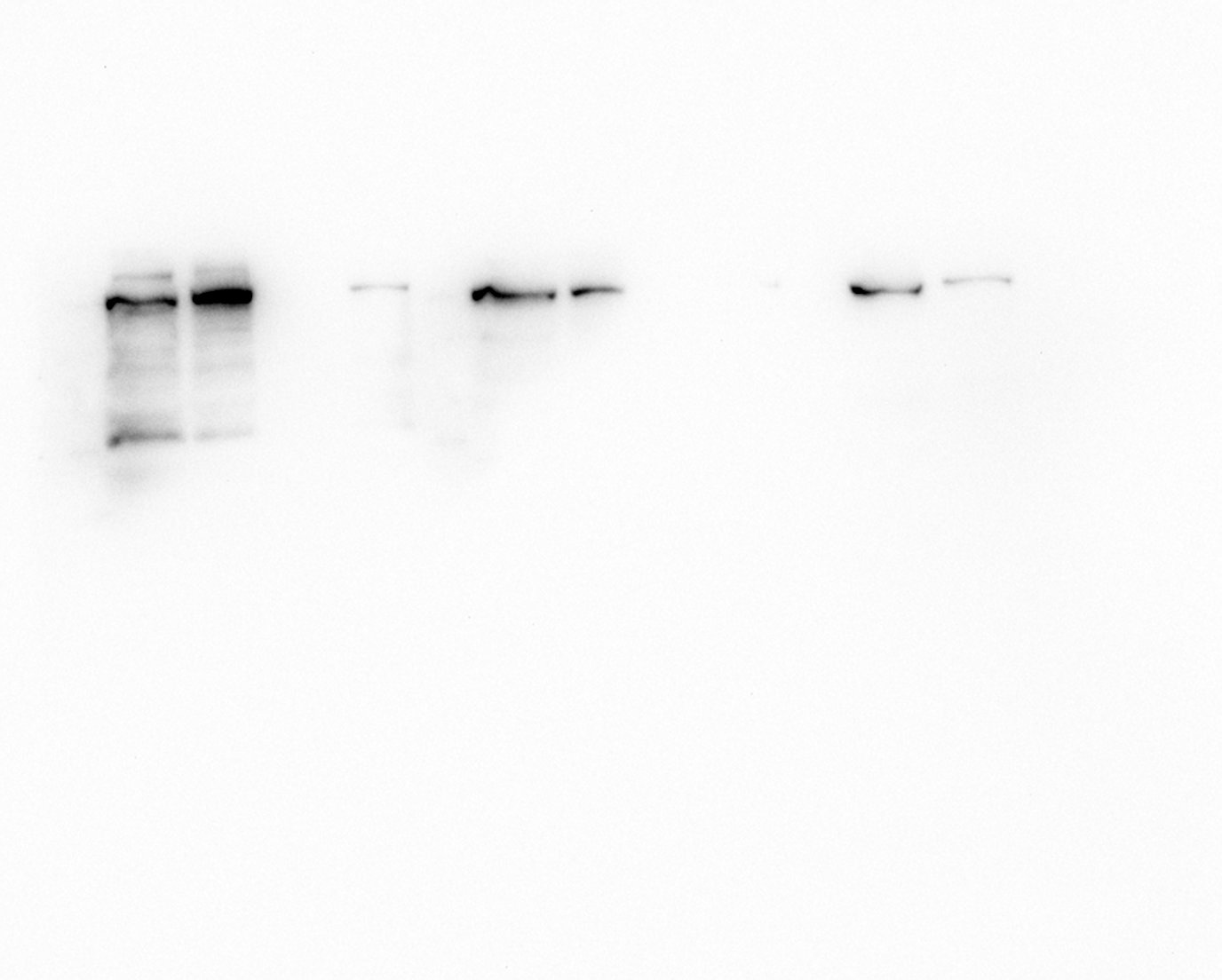

Supplement: Figure 4—source data 1. [file elife-94898-fig4-data1.zip › Fig4L-Flag.Tif]

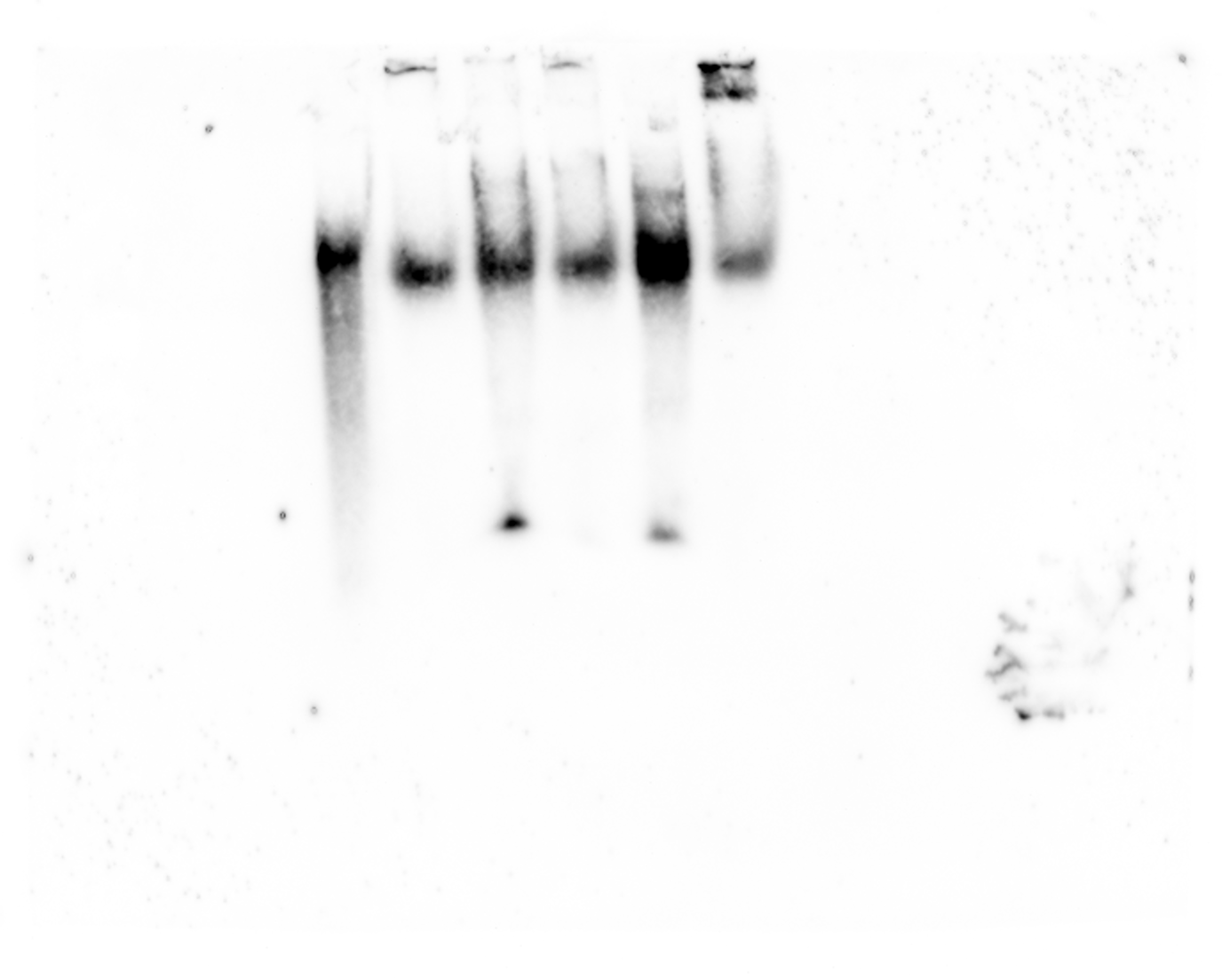

Supplement: Figure 4—source data 1. [file elife-94898-fig4-data1.zip › Fig4M.Tif]

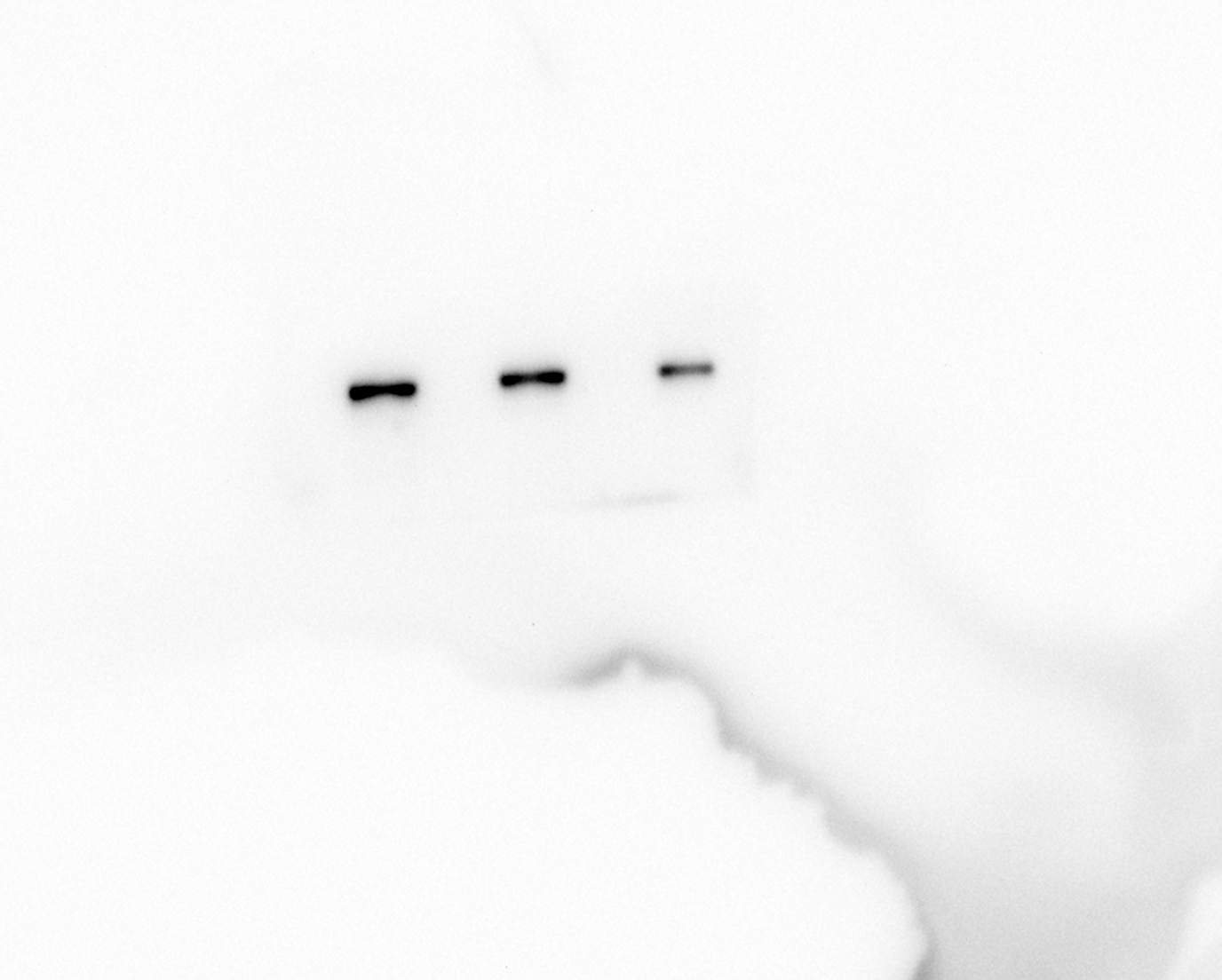

Supplement: Figure 4—source data 1. [file elife-94898-fig4-data1.zip › Fig4B-mmiMDA5-Flag.Tif]

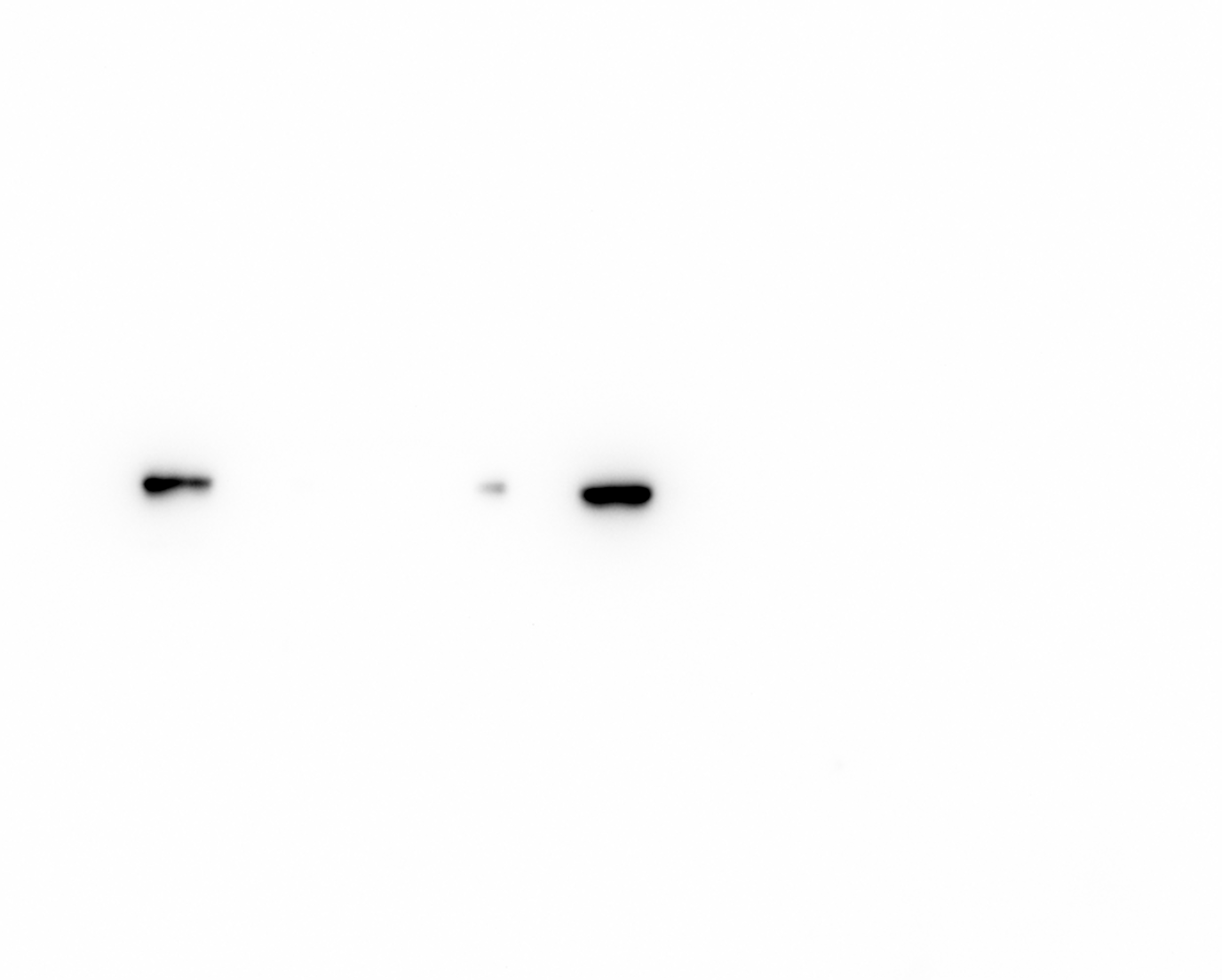

Supplement: Figure 4—source data 1. [file elife-94898-fig4-data1.zip › Fig4B-Tubulin.Tif]

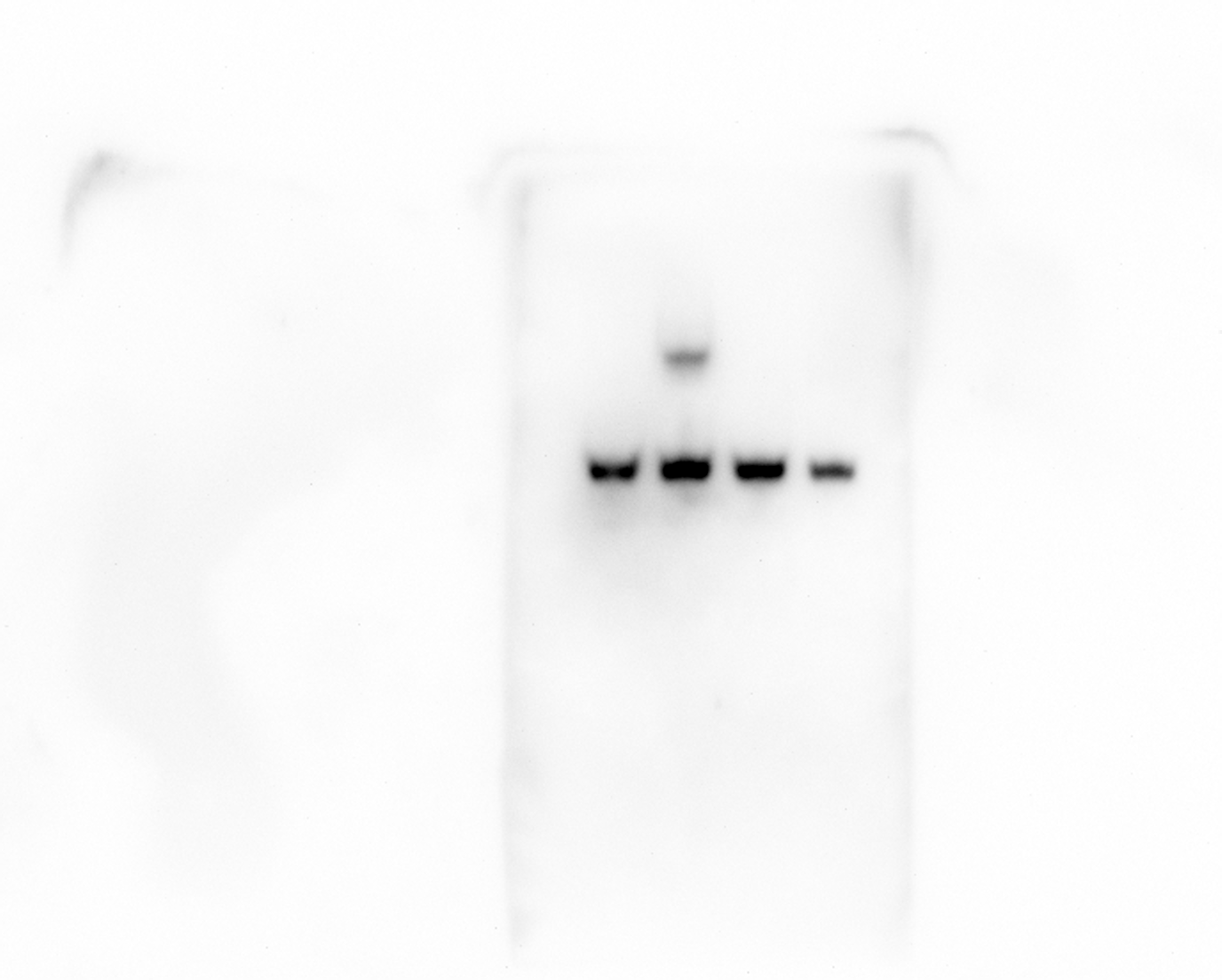

Supplement: Figure 4—source data 1. [file elife-94898-fig4-data1.zip › Fig4C-HA.Tif]

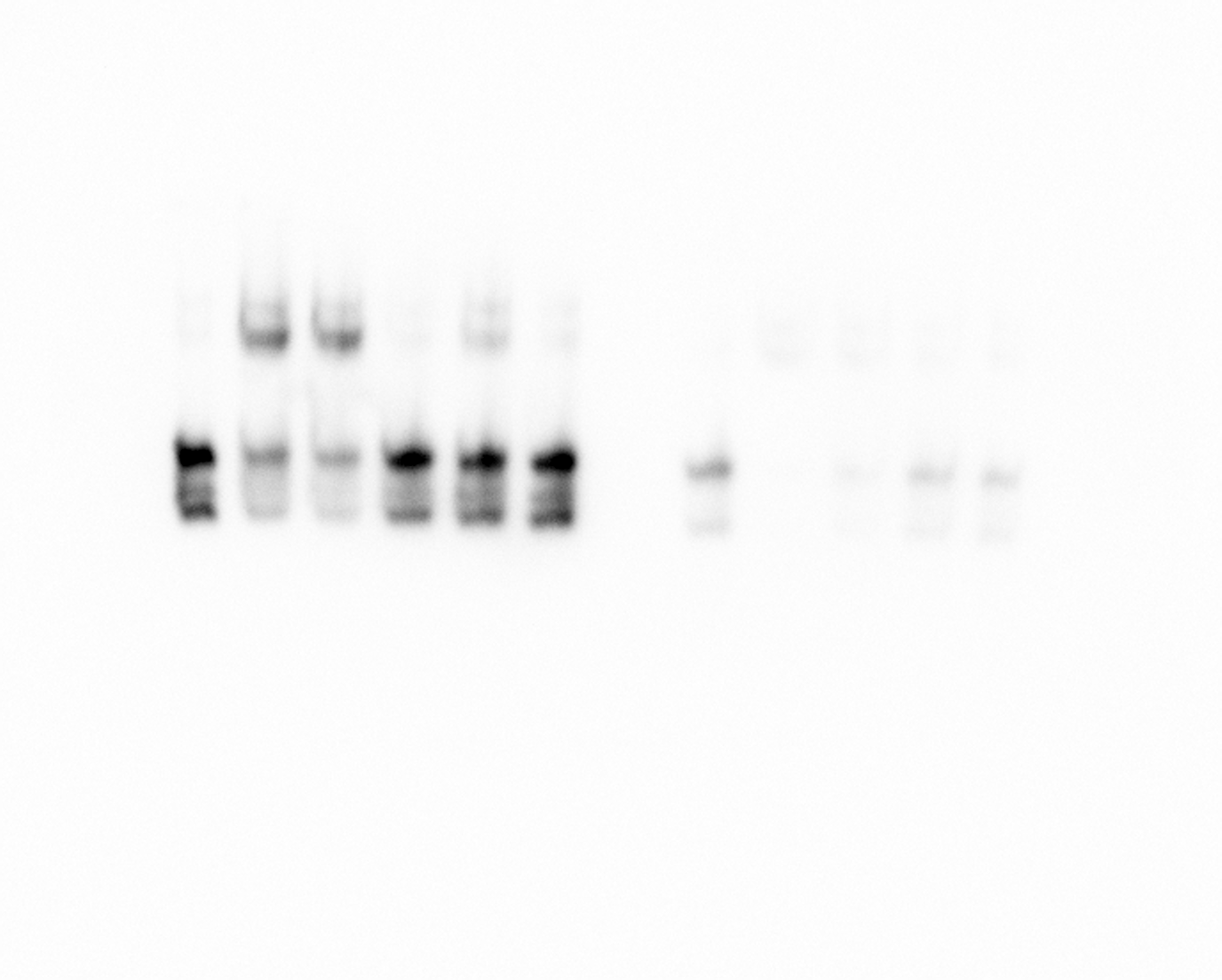

Supplement: Figure 4—source data 1. [file elife-94898-fig4-data1.zip › Fig4D-HA.Tif]

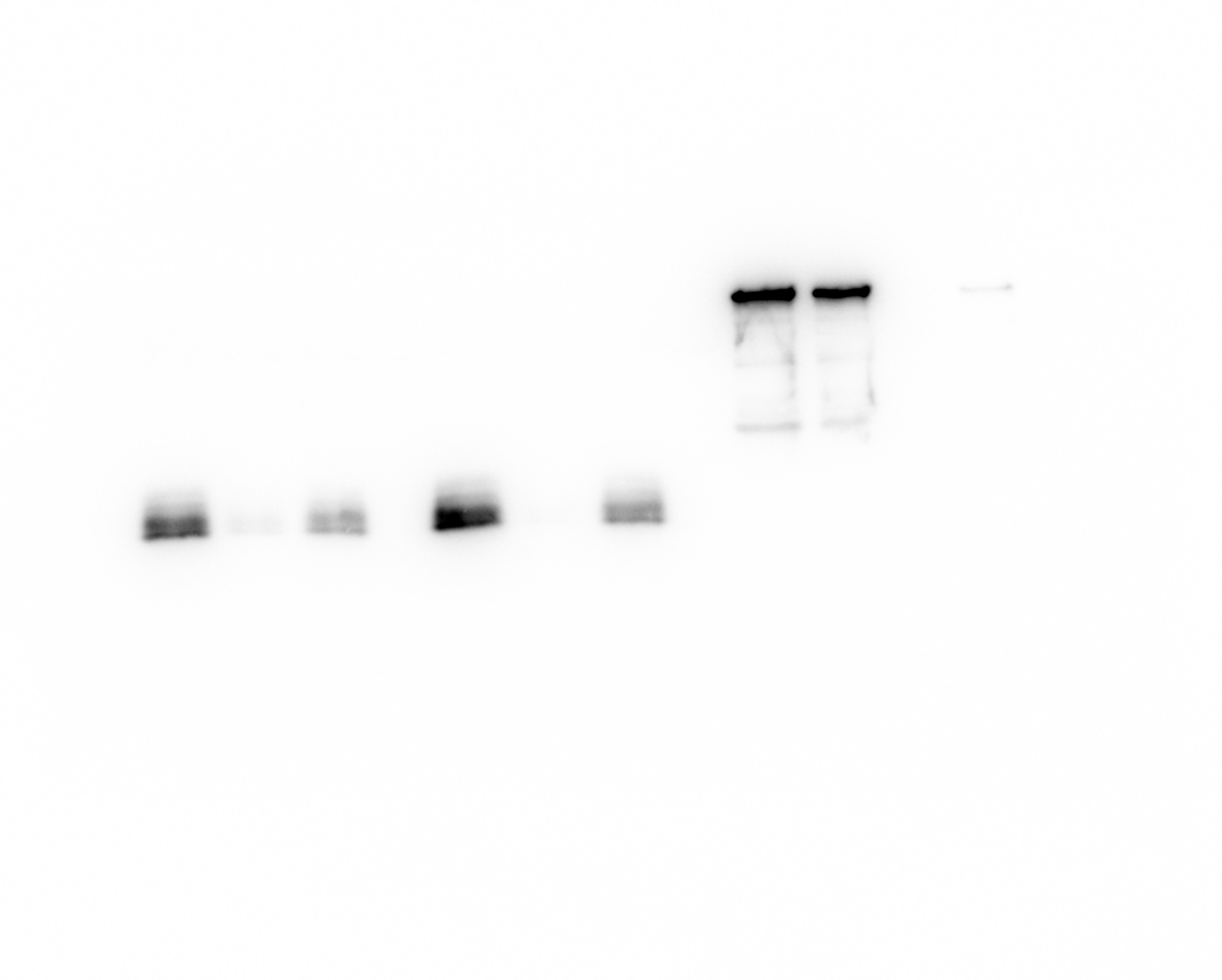

Supplement: Figure 4—source data 1. [file elife-94898-fig4-data1.zip › Fig4E-Flag.Tif]

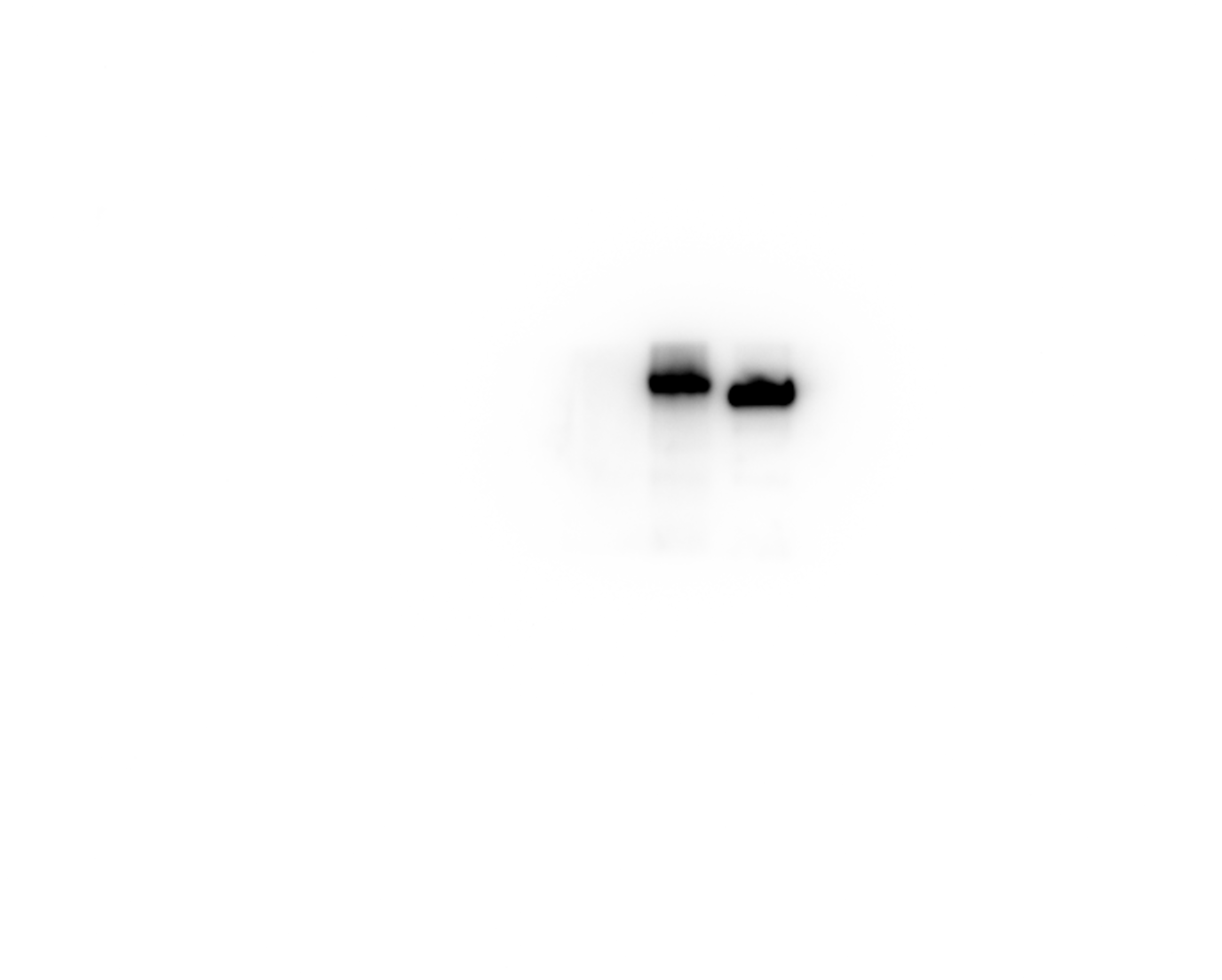

Supplement: Figure 4—source data 1. [file elife-94898-fig4-data1.zip › Fig4F-His.Tif]

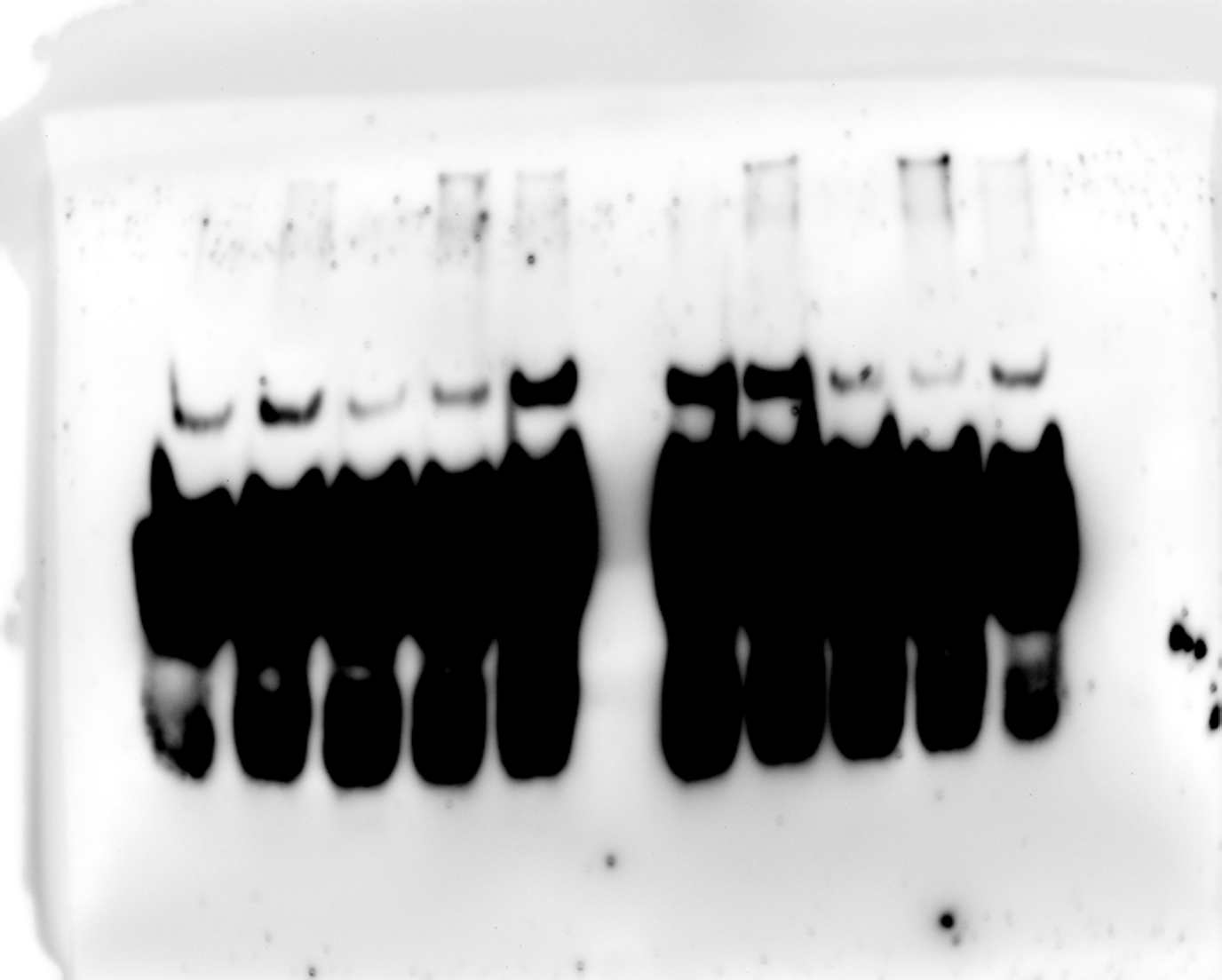

Supplement: Figure 4—source data 1. [file elife-94898-fig4-data1.zip › Fig4G.Tif]

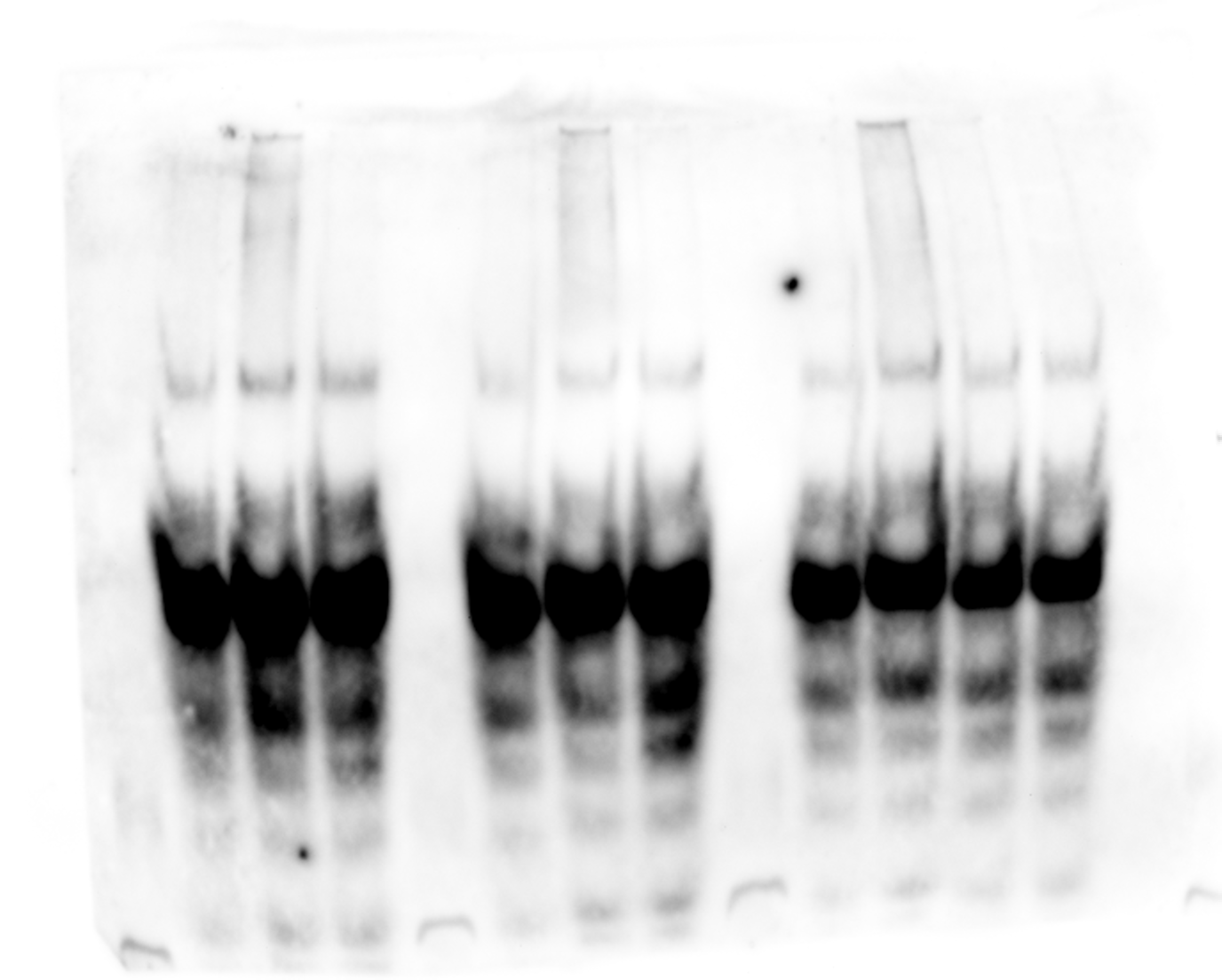

Supplement: Figure 4—source data 1. [file elife-94898-fig4-data1.zip › Fig4H.Tif]

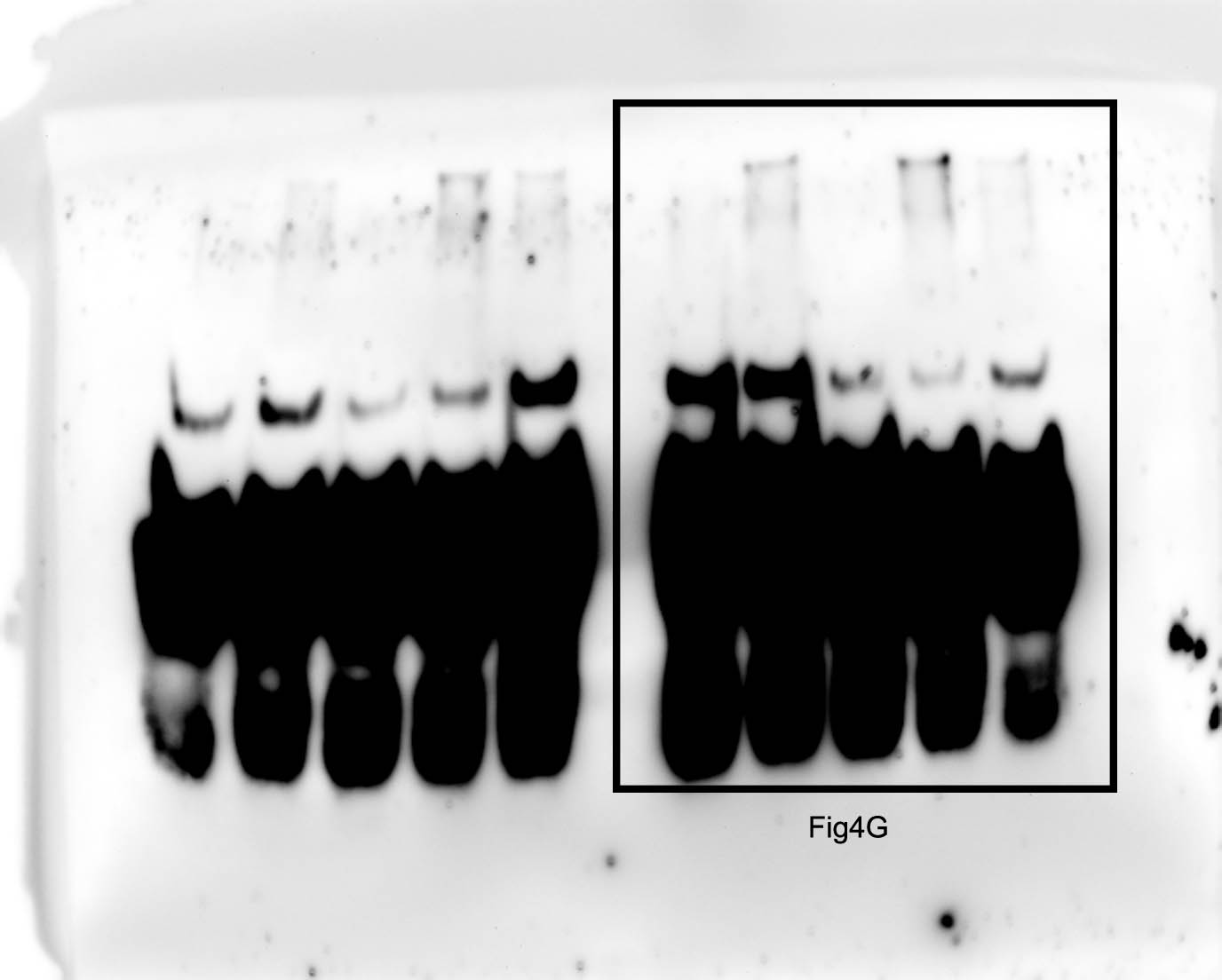

Supplement: Figure 4—source data 2. [file elife-94898-fig4-data2.zip › Fig4G.jpg]

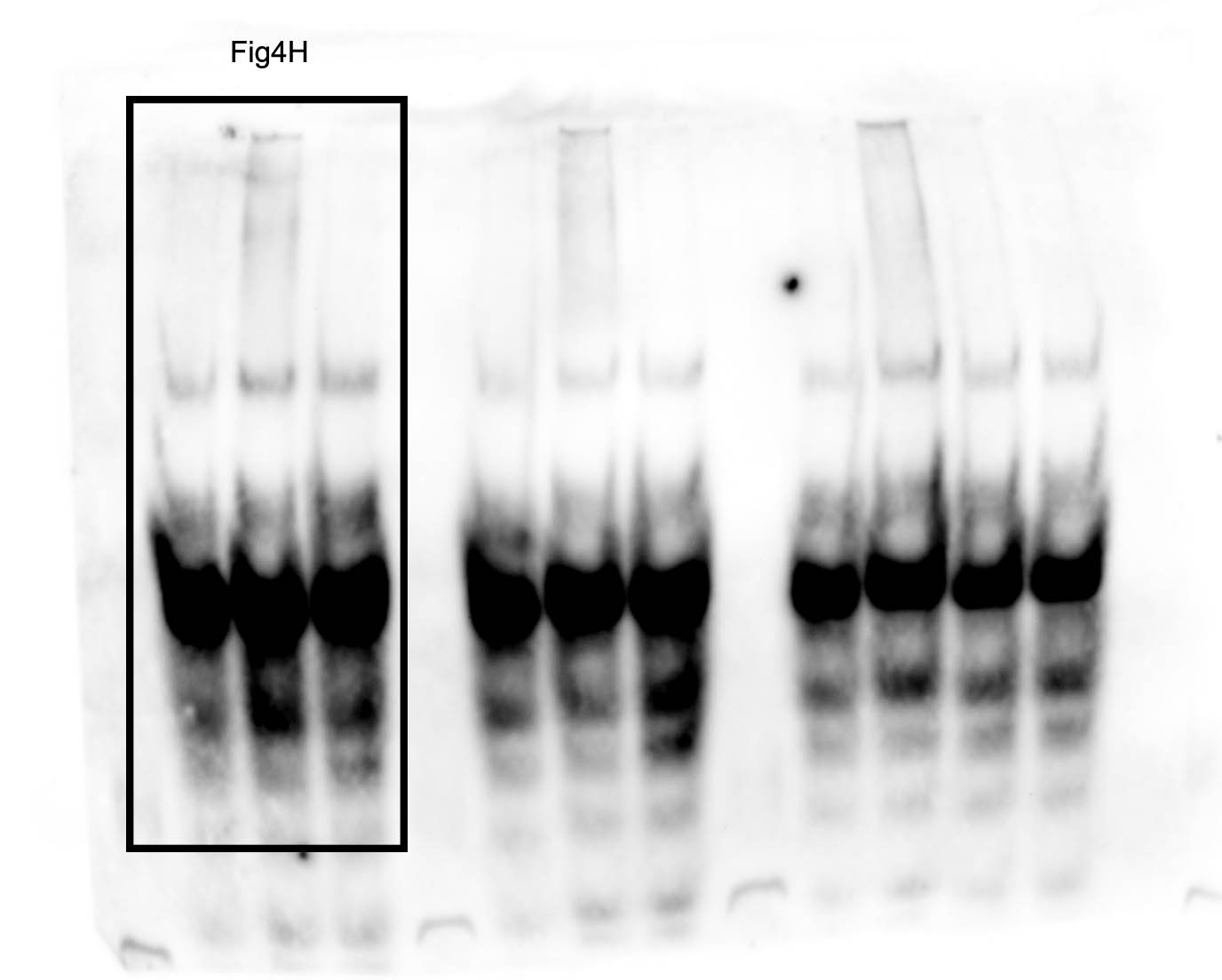

Supplement: Figure 4—source data 2. [file elife-94898-fig4-data2.zip › Fig4H.jpg]

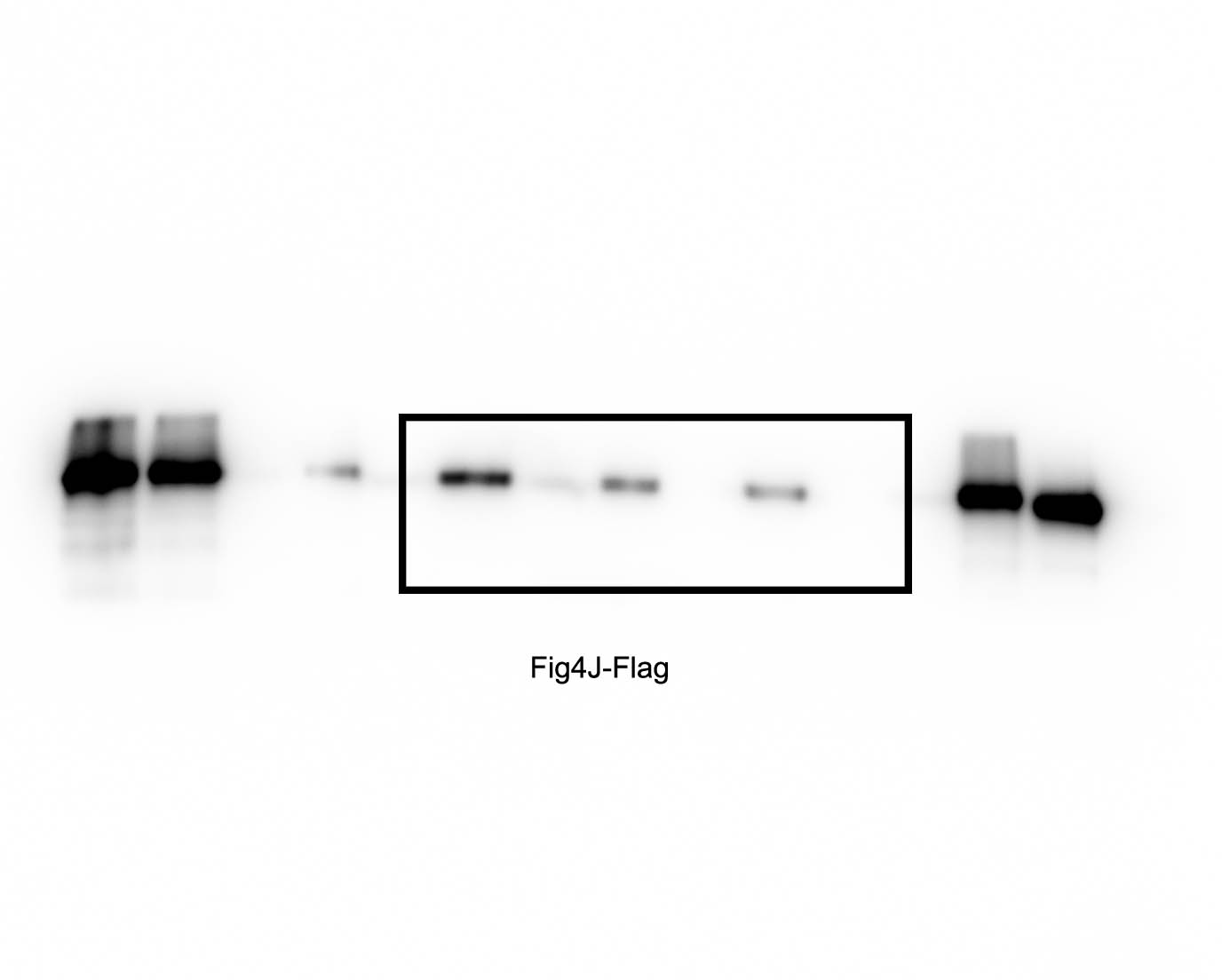

Supplement: Figure 4—source data 2. [file elife-94898-fig4-data2.zip › Fig4J-Flag.jpg]

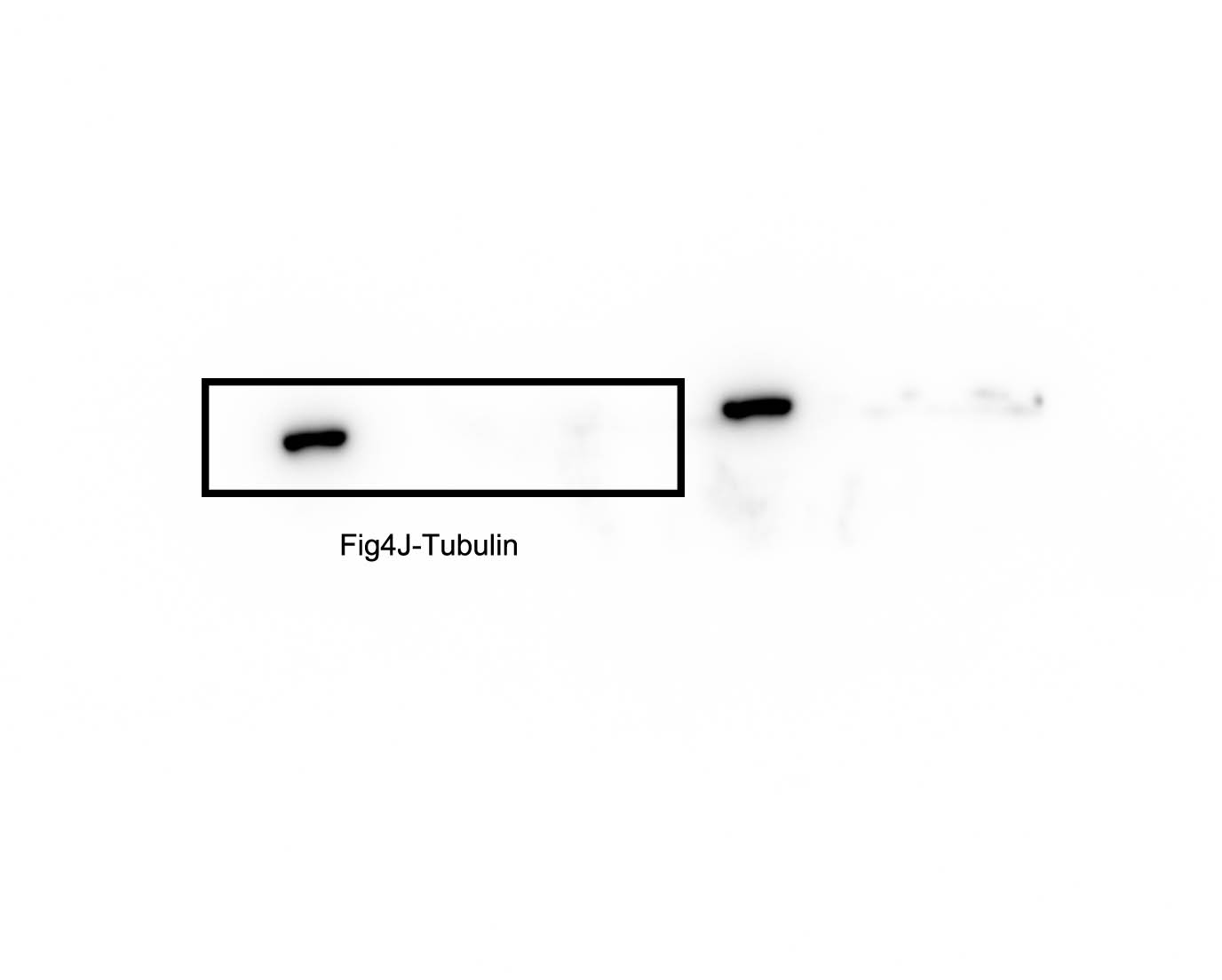

Supplement: Figure 4—source data 2. [file elife-94898-fig4-data2.zip › Fig4J-Tubulin.jpg]

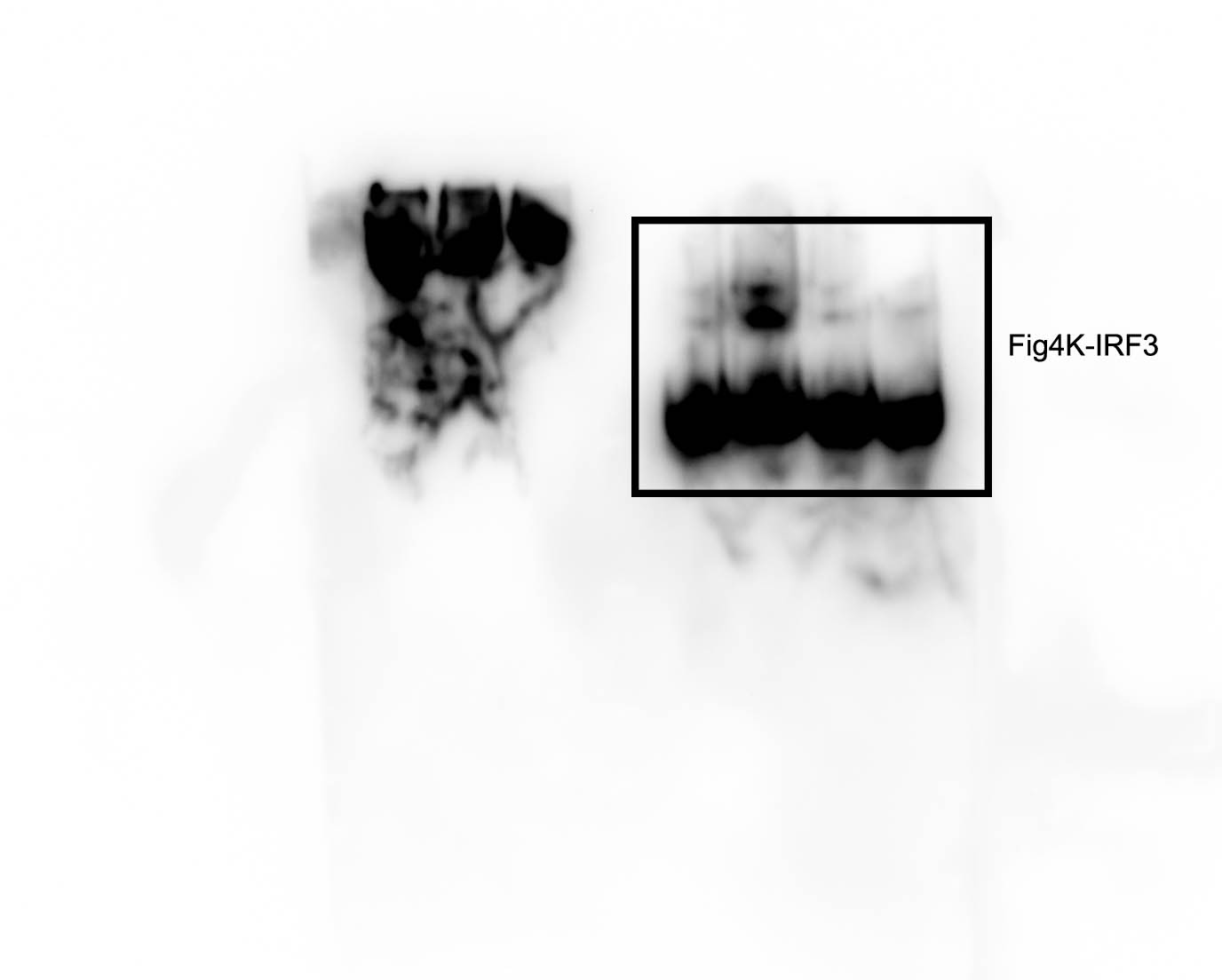

Supplement: Figure 4—source data 2. [file elife-94898-fig4-data2.zip › Fig4K-IRF3.jpg]

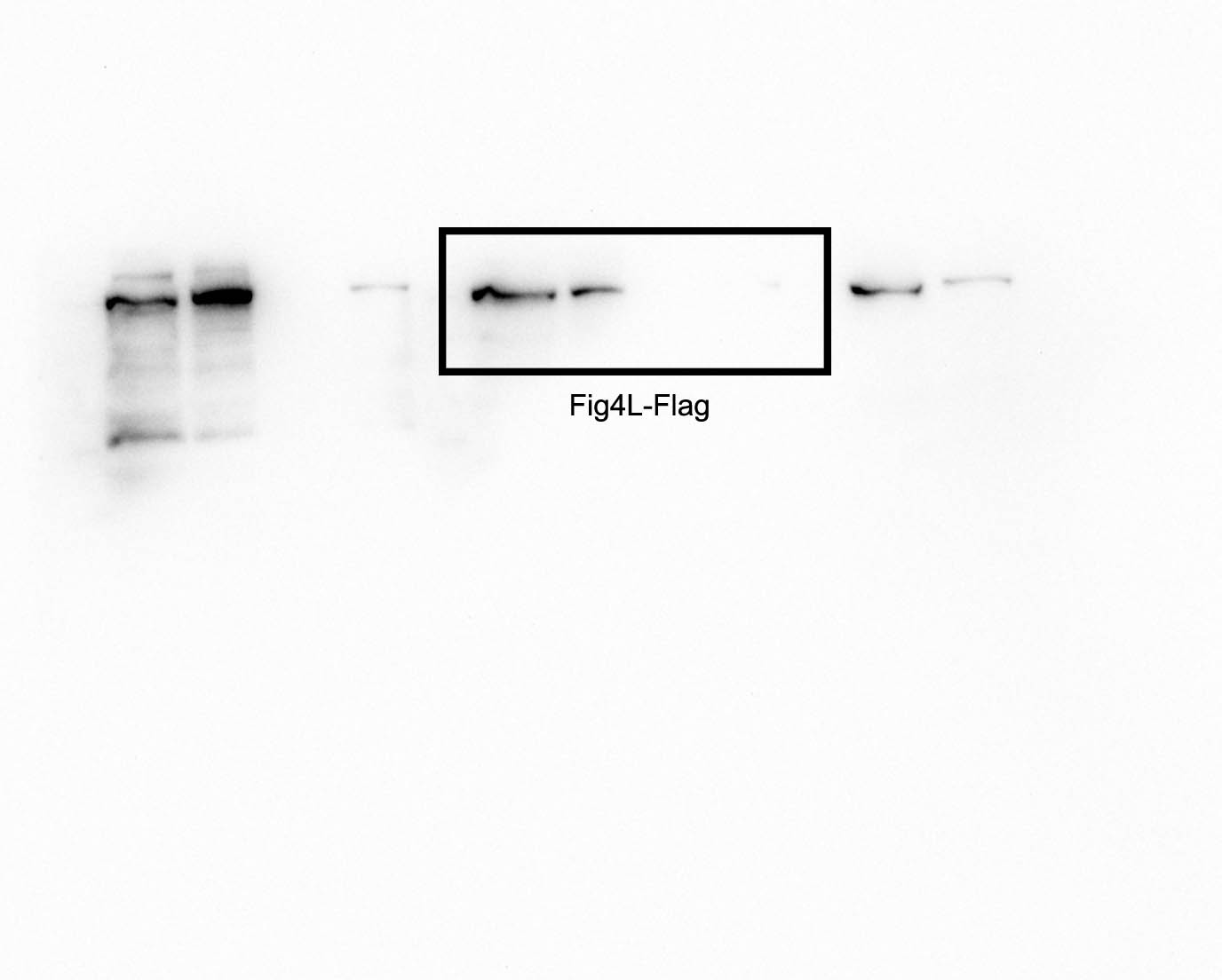

Supplement: Figure 4—source data 2. [file elife-94898-fig4-data2.zip › Fig4L-Flag.jpg]

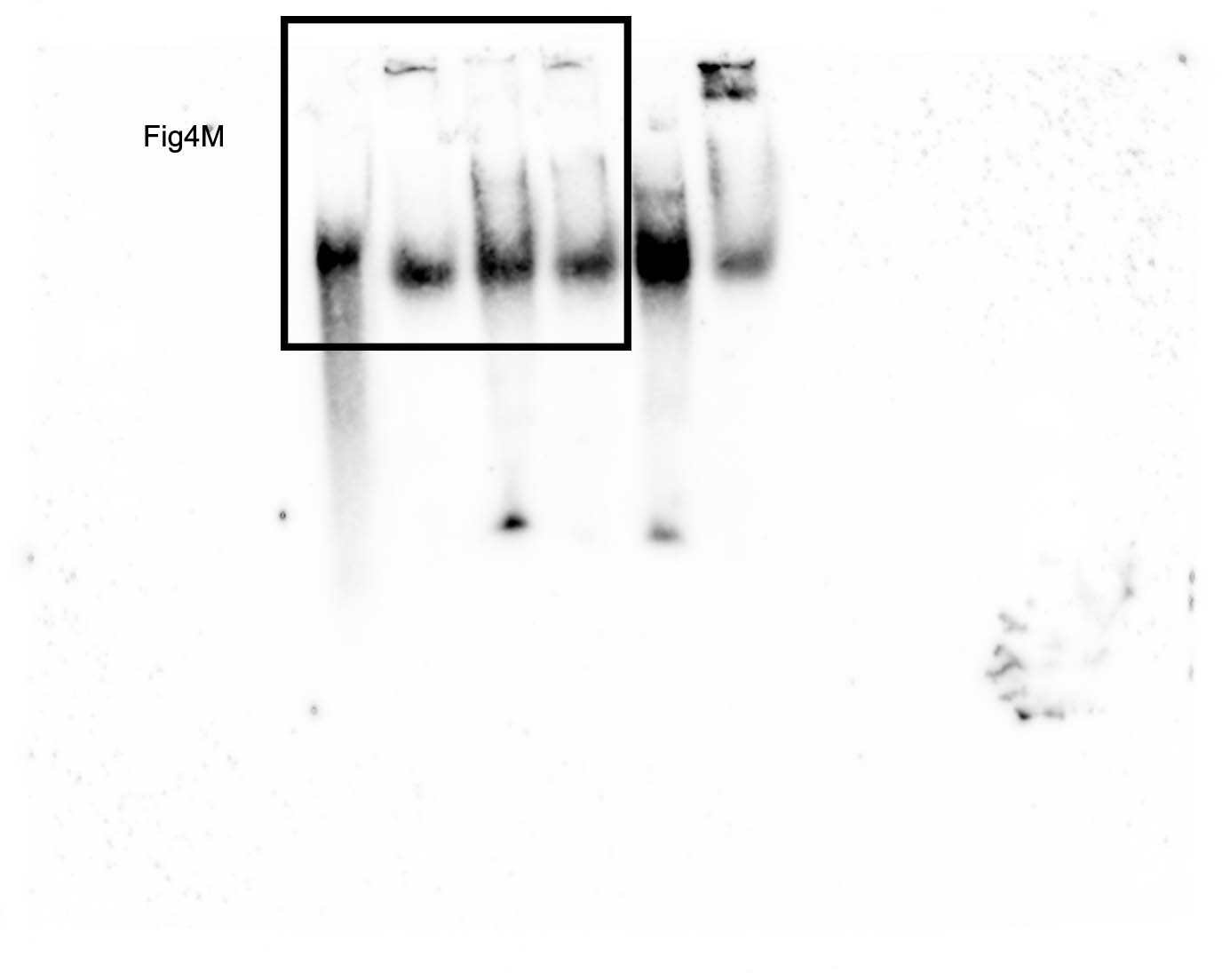

Supplement: Figure 4—source data 2. [file elife-94898-fig4-data2.zip › Fig4M.jpg]

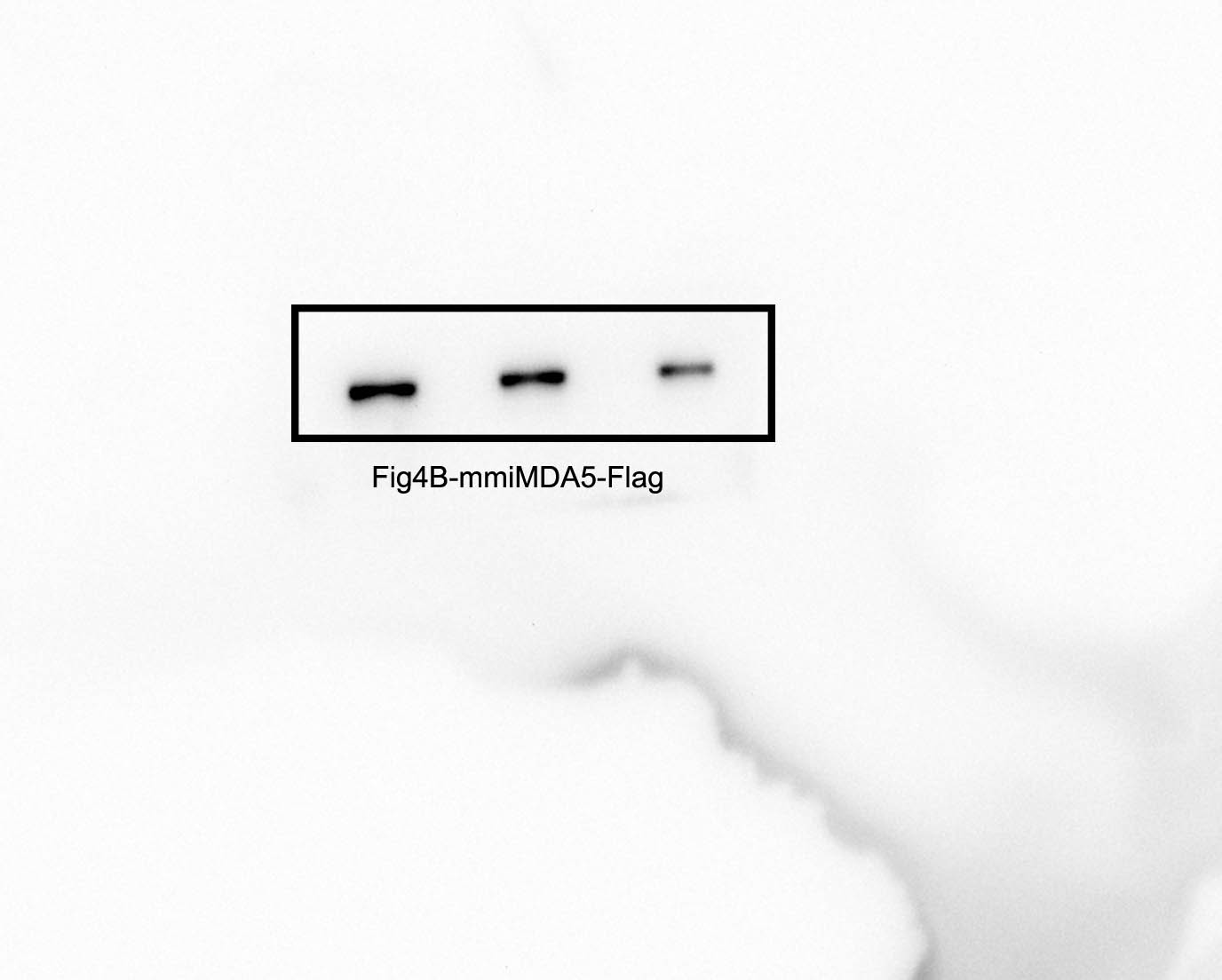

Supplement: Figure 4—source data 2. [file elife-94898-fig4-data2.zip › Fig4B-mmiMDA5-Flag.jpg]

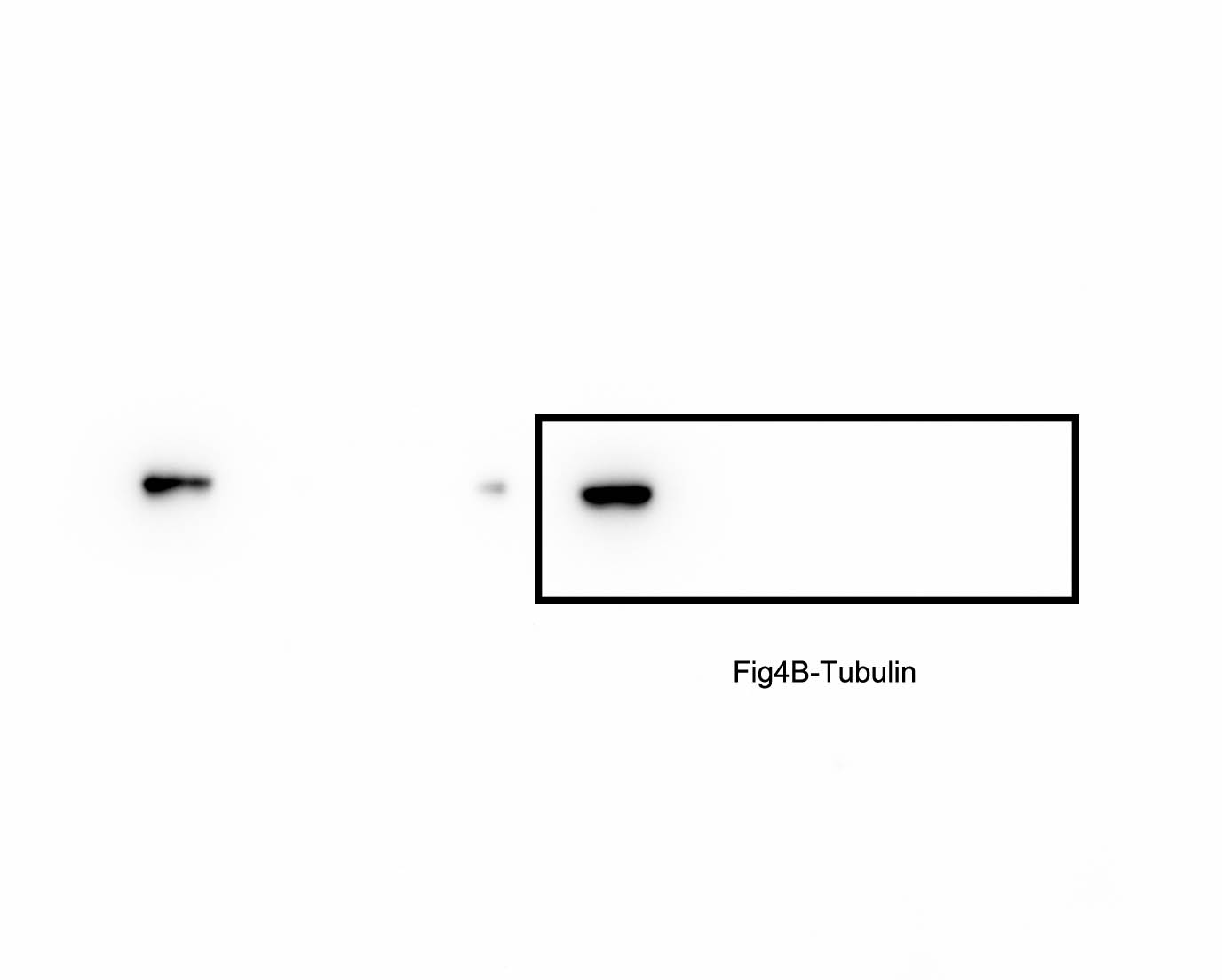

Supplement: Figure 4—source data 2. [file elife-94898-fig4-data2.zip › Fig4B-Tubulin.jpg]

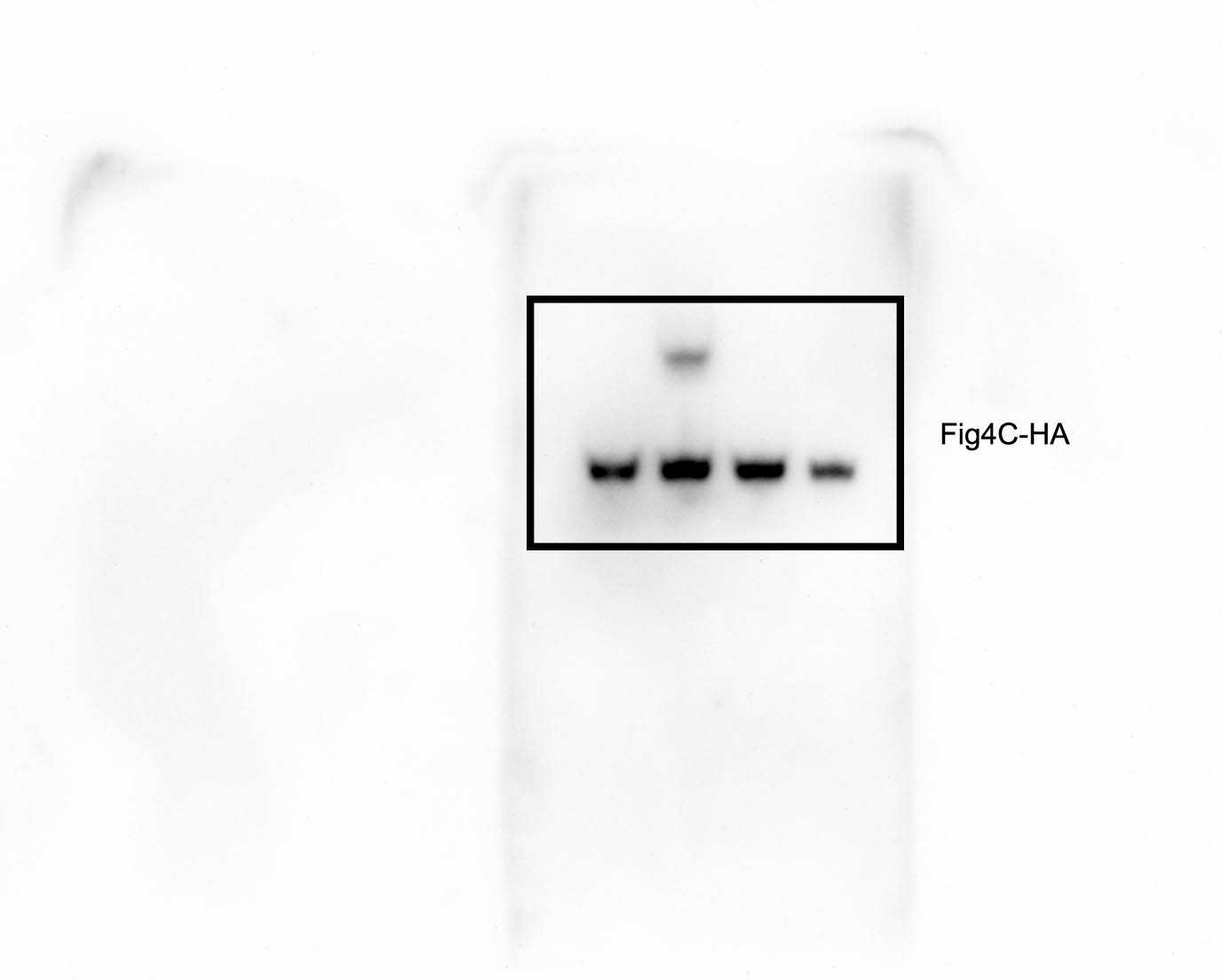

Supplement: Figure 4—source data 2. [file elife-94898-fig4-data2.zip › Fig4C-HA.jpg]

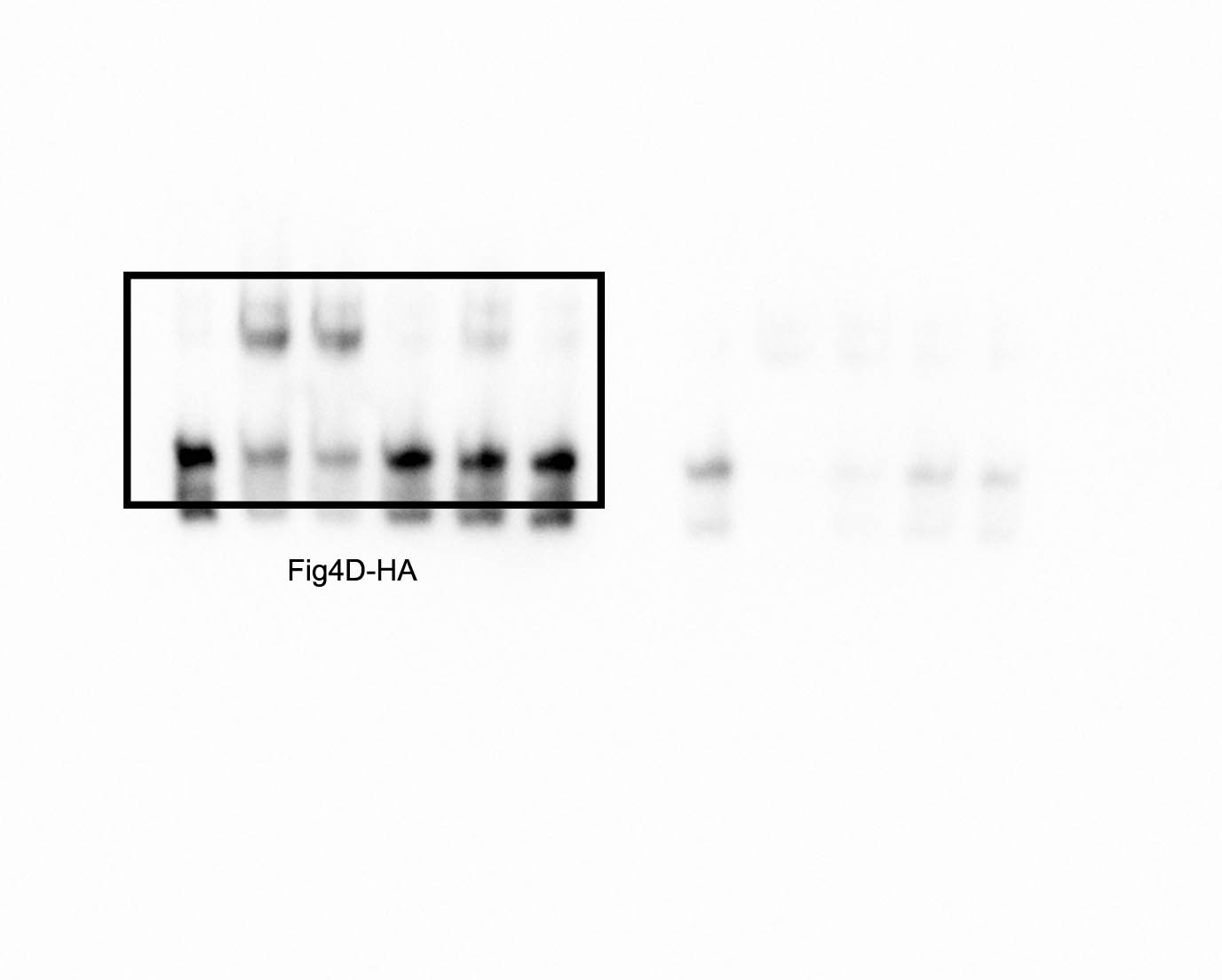

Supplement: Figure 4—source data 2. [file elife-94898-fig4-data2.zip › Fig4D-HA.jpg]

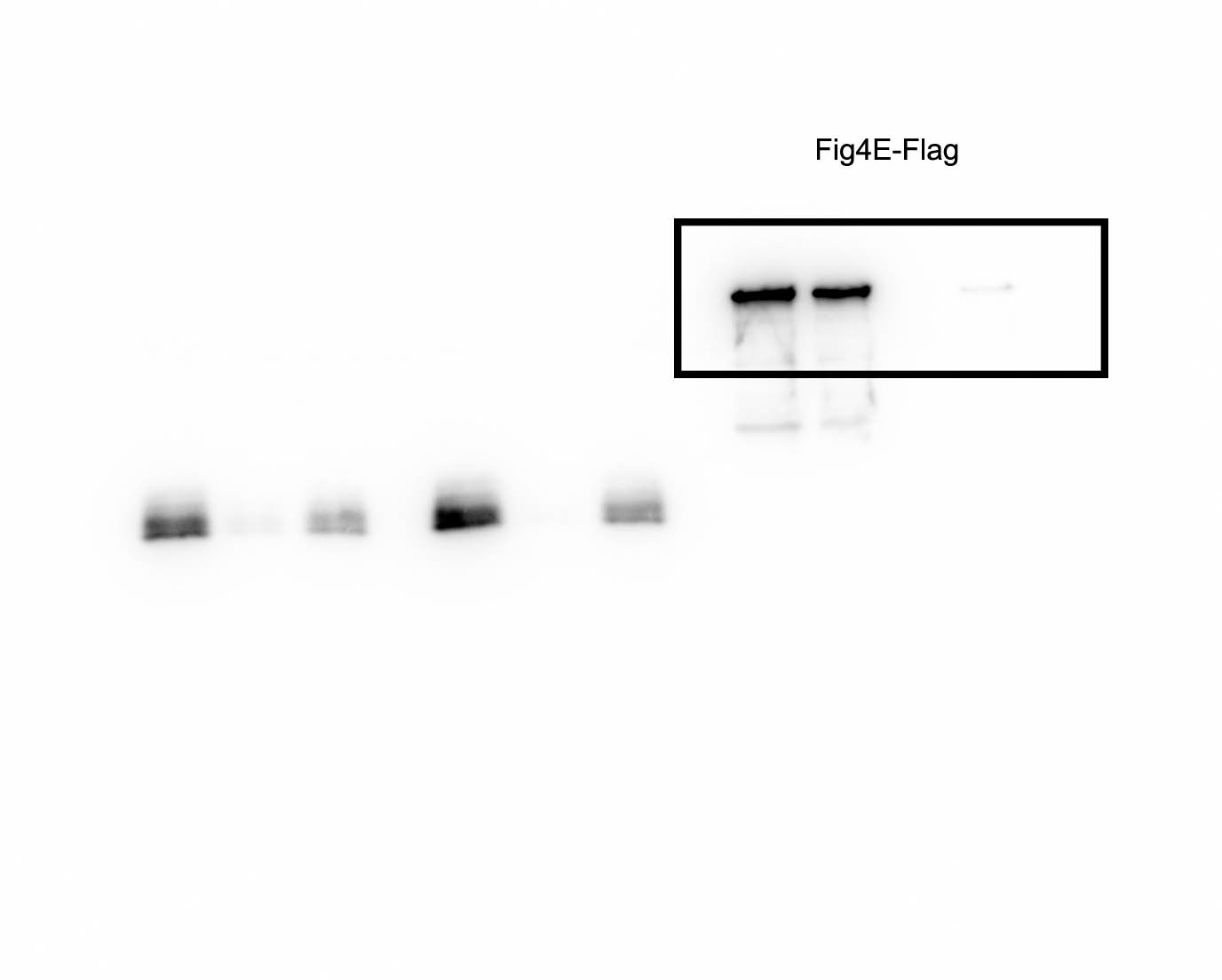

Supplement: Figure 4—source data 2. [file elife-94898-fig4-data2.zip › Fig4E-Flag.jpg]

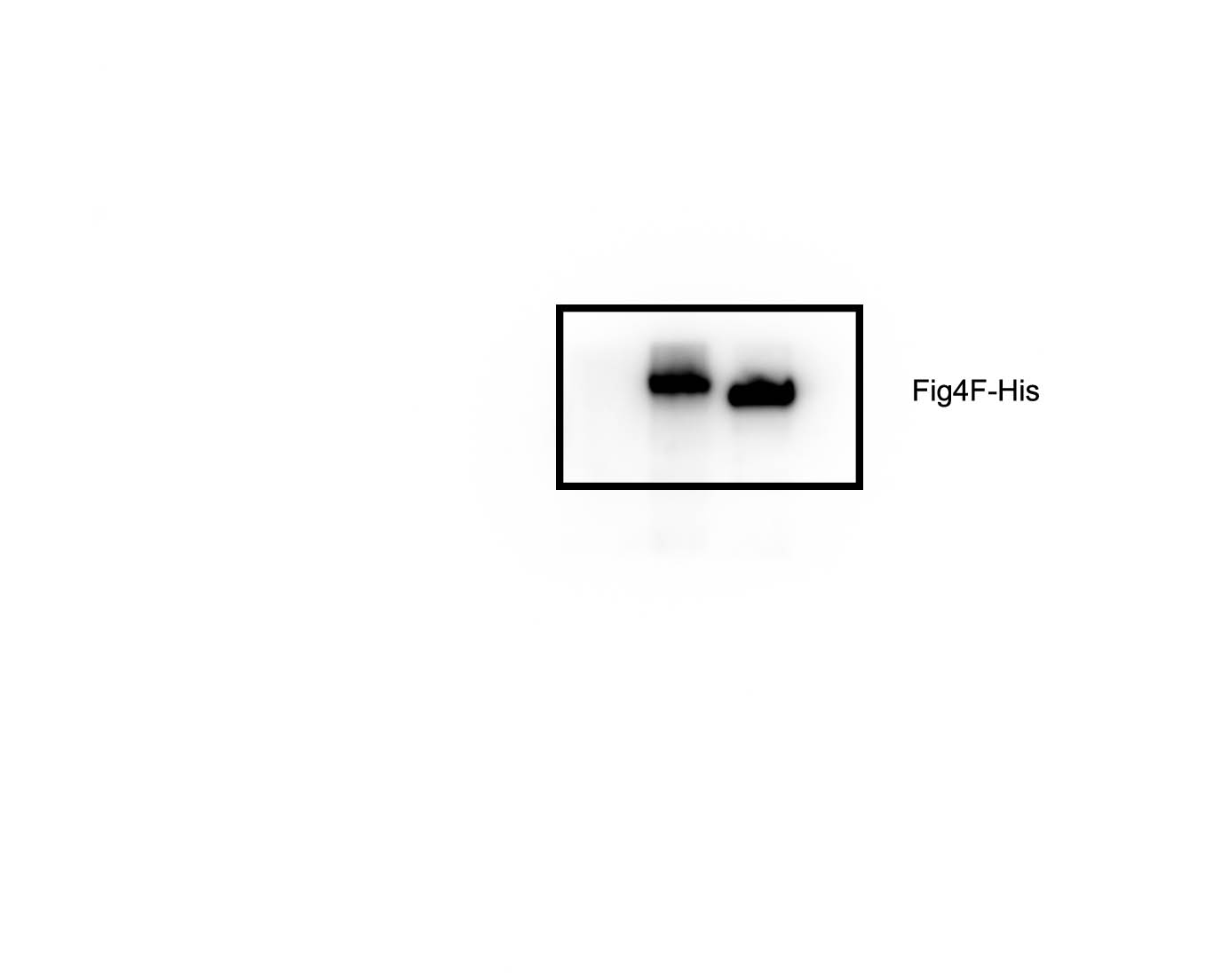

Supplement: Figure 4—source data 2. [file elife-94898-fig4-data2.zip › Fig4F-His.jpg]

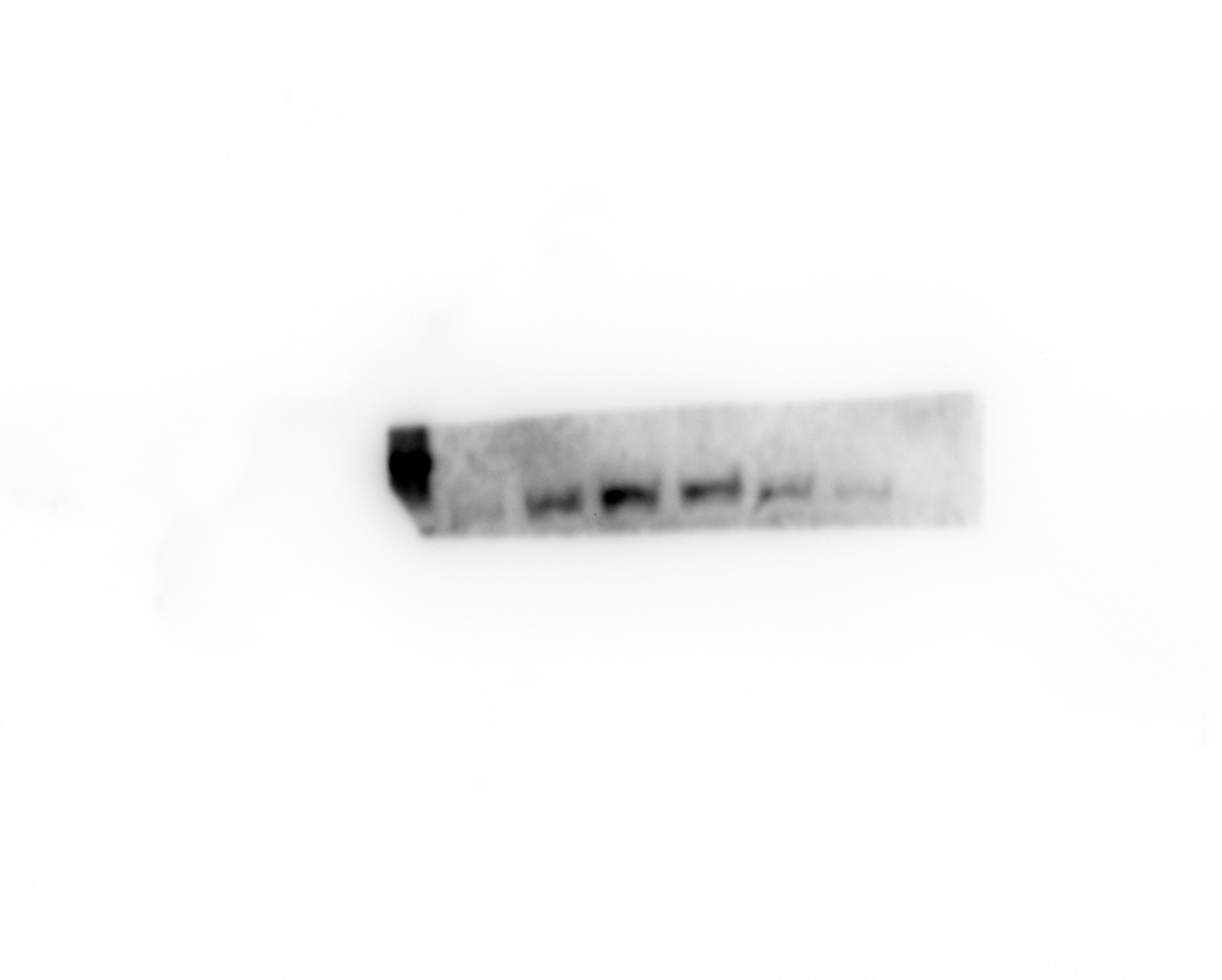

Supplement: Figure 5—source data 2. [file elife-94898-fig5-data2.zip › Fig5J-MDA5.Tif]

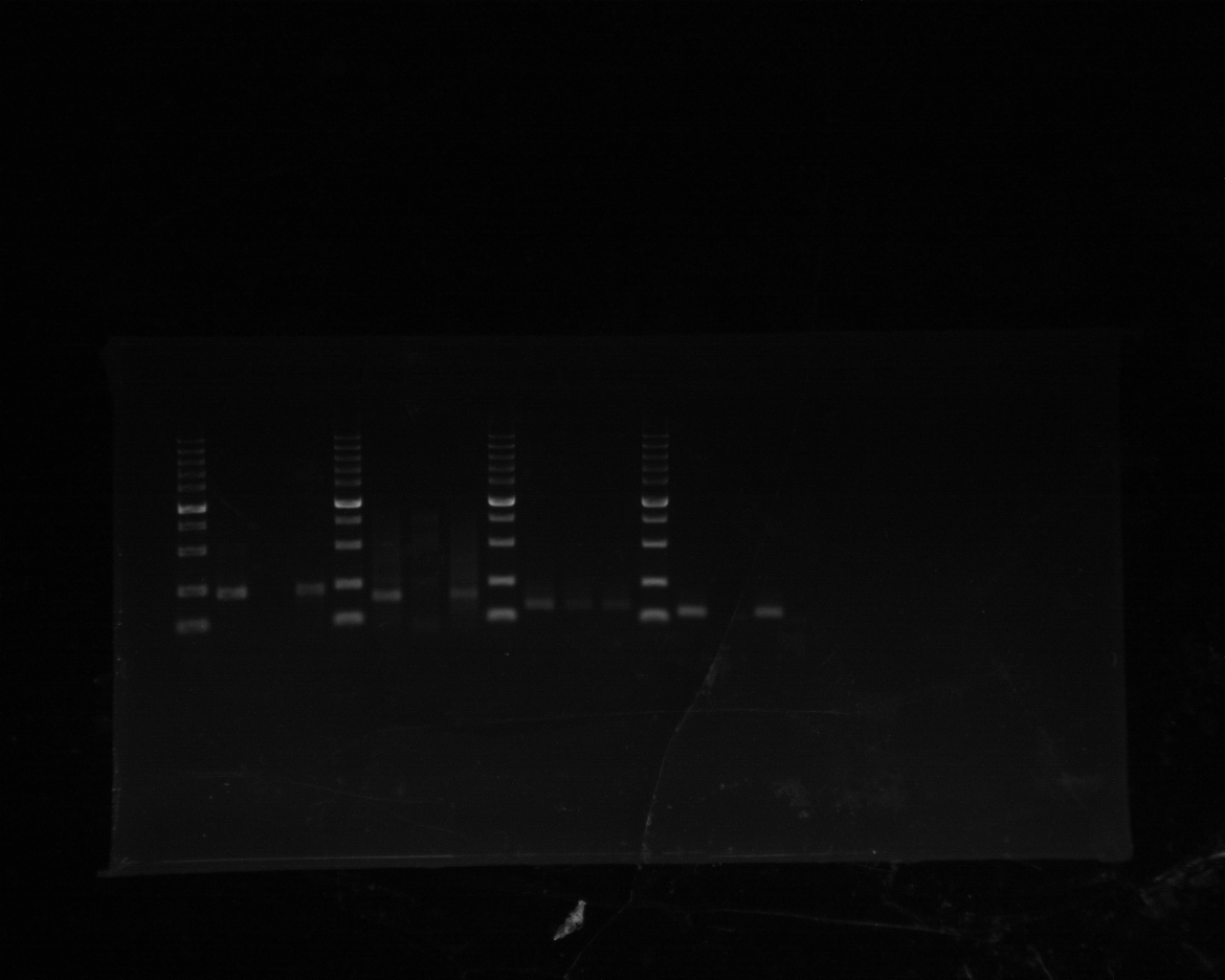

Supplement: Figure 5—source data 2. [file elife-94898-fig5-data2.zip › Fig5E-MDA5-m6A.tif]

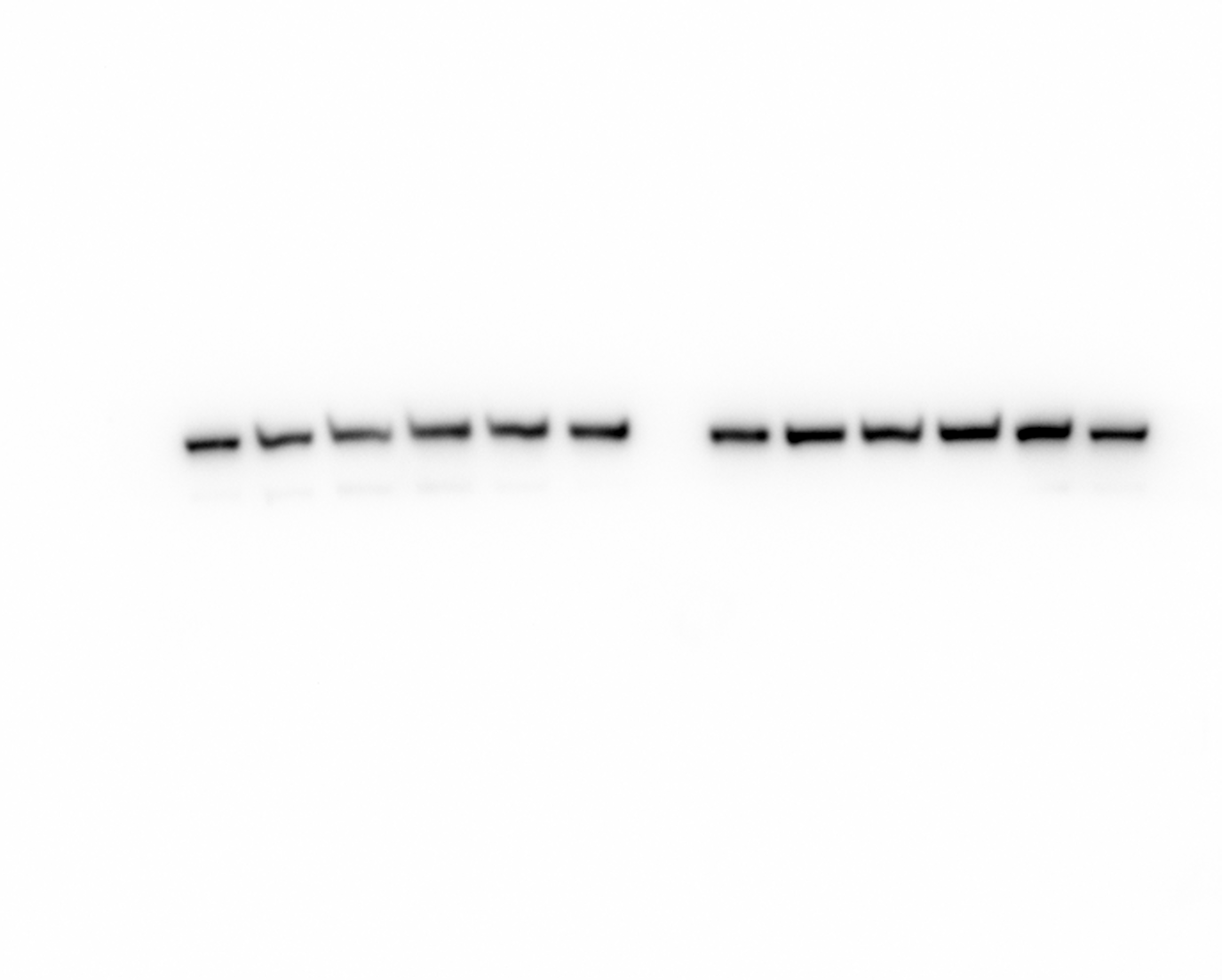

Supplement: Figure 5—source data 2. [file elife-94898-fig5-data2.zip › Fig5I+5J-Tubulin.Tif]

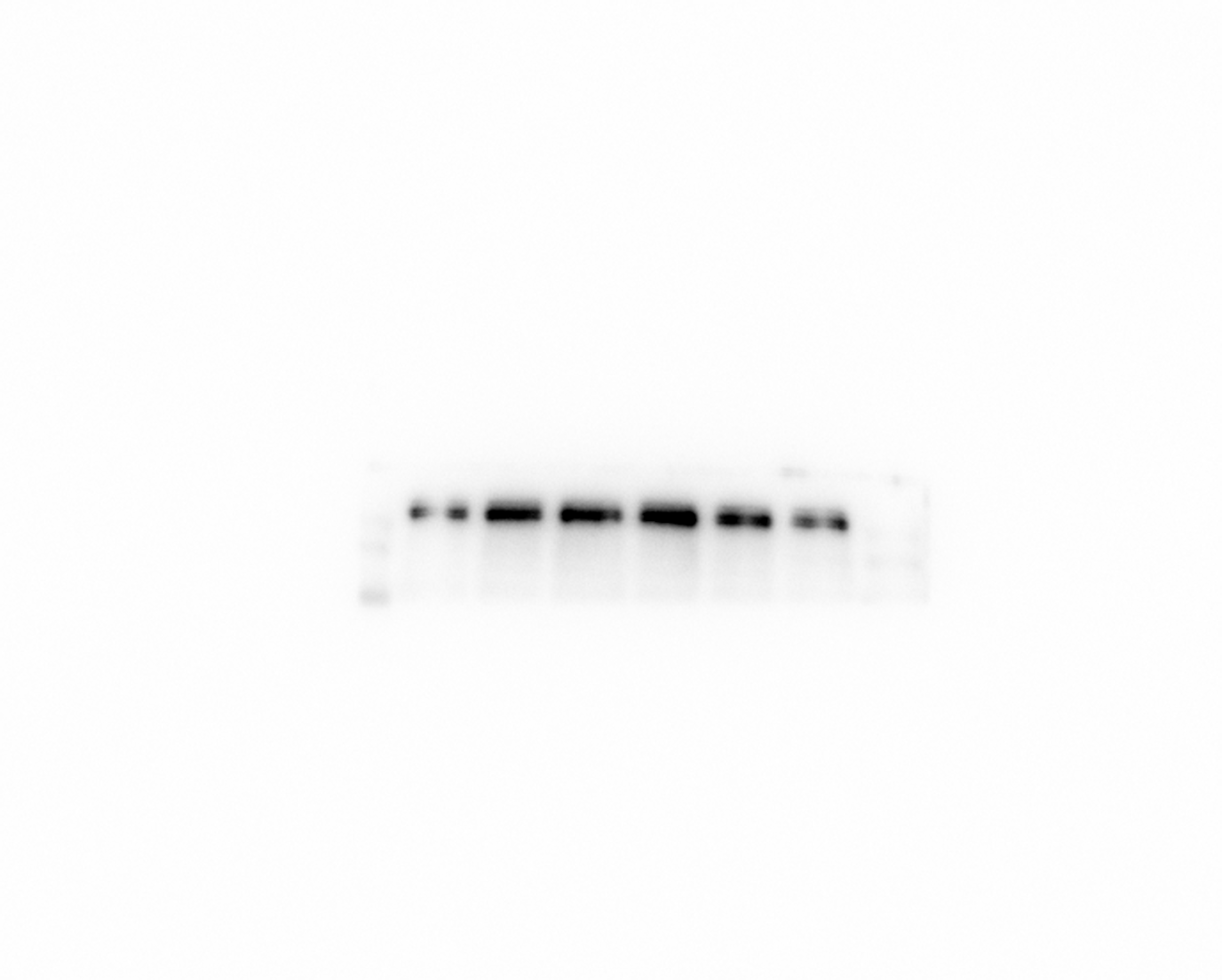

Supplement: Figure 5—source data 2. [file elife-94898-fig5-data2.zip › Fig5I-MDA5.Tif]

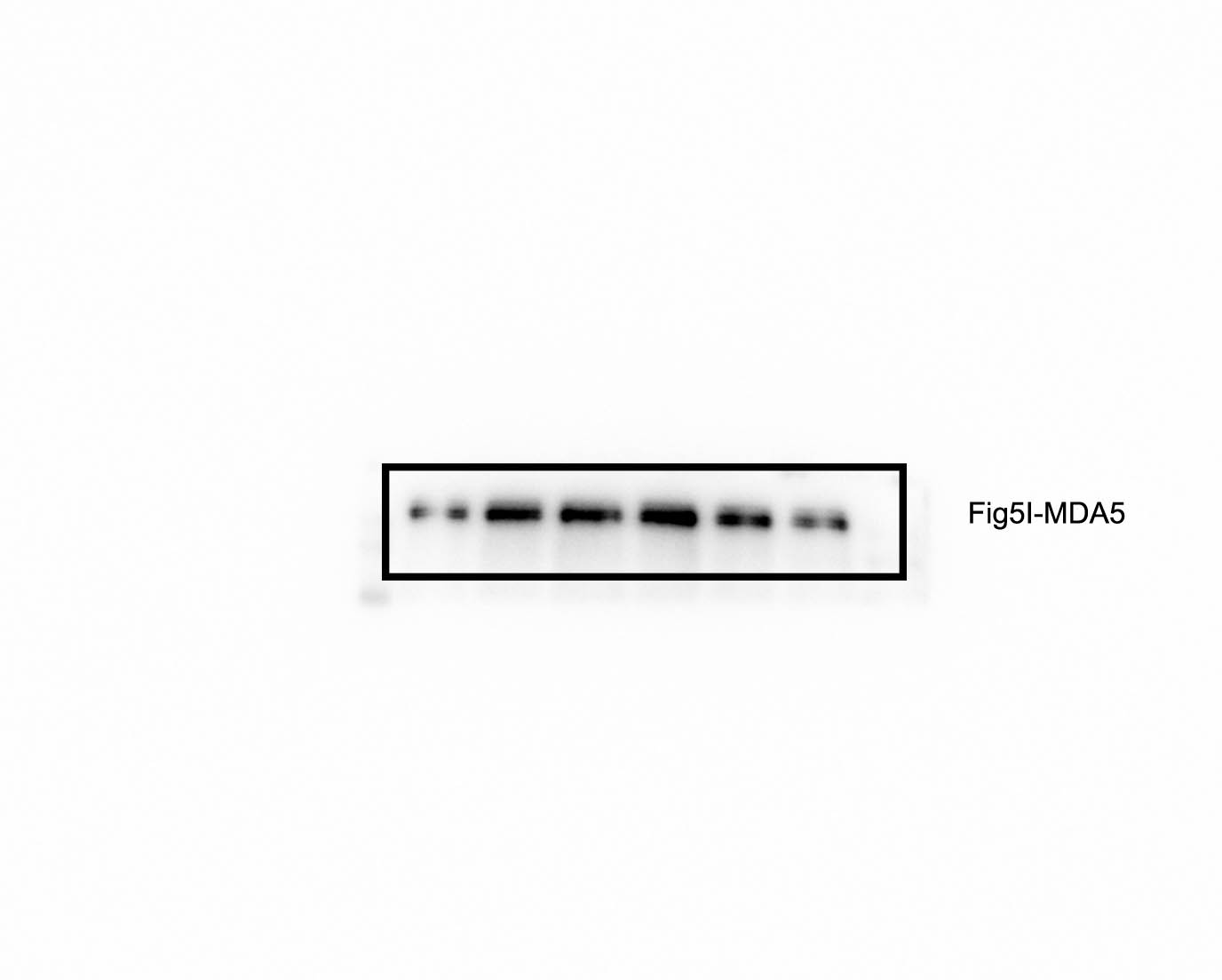

Supplement: Figure 5—source data 3. [file elife-94898-fig5-data3.zip › Fig5I-MDA5.jpg]

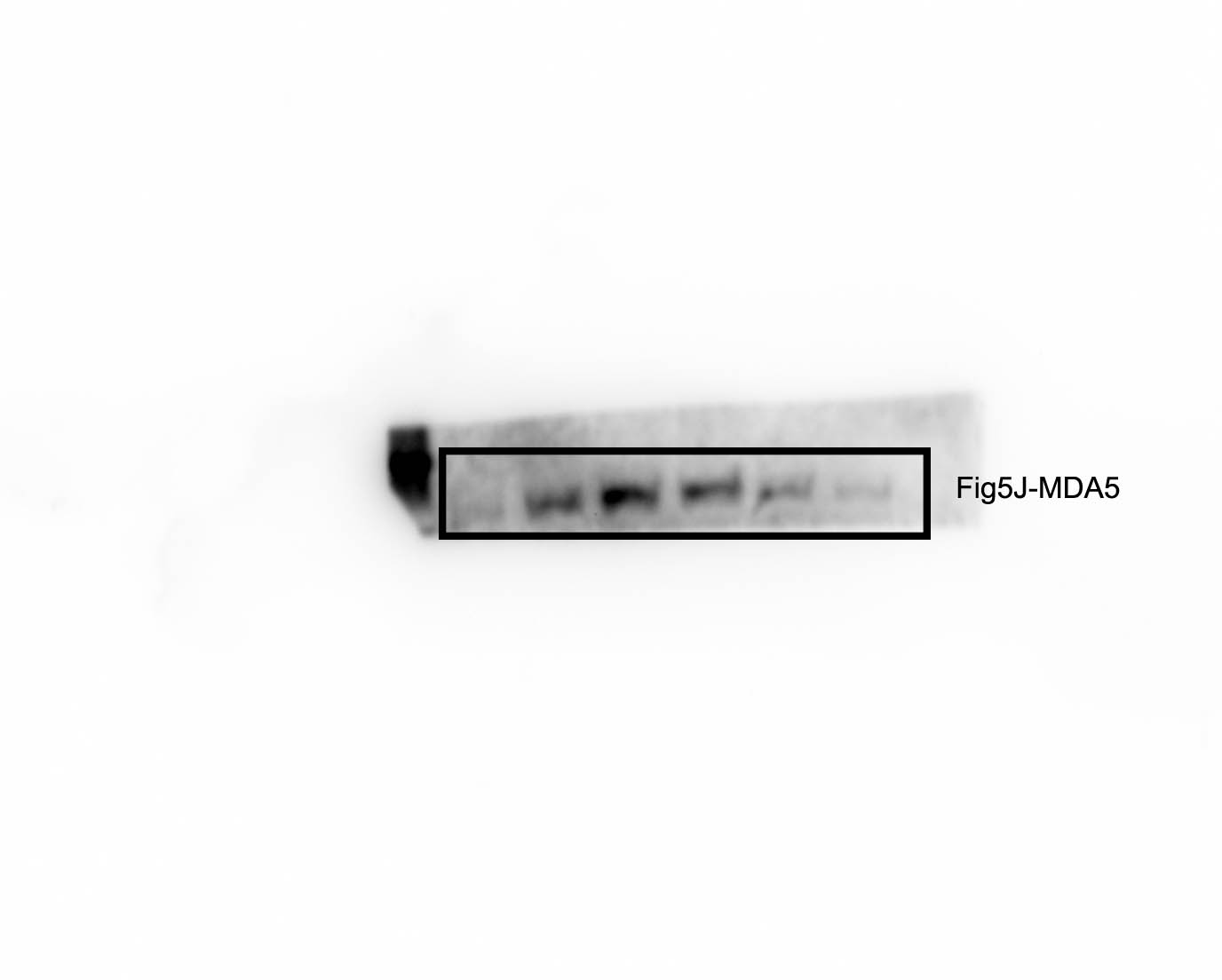

Supplement: Figure 5—source data 3. [file elife-94898-fig5-data3.zip › Fig5J-MDA5.jpg]

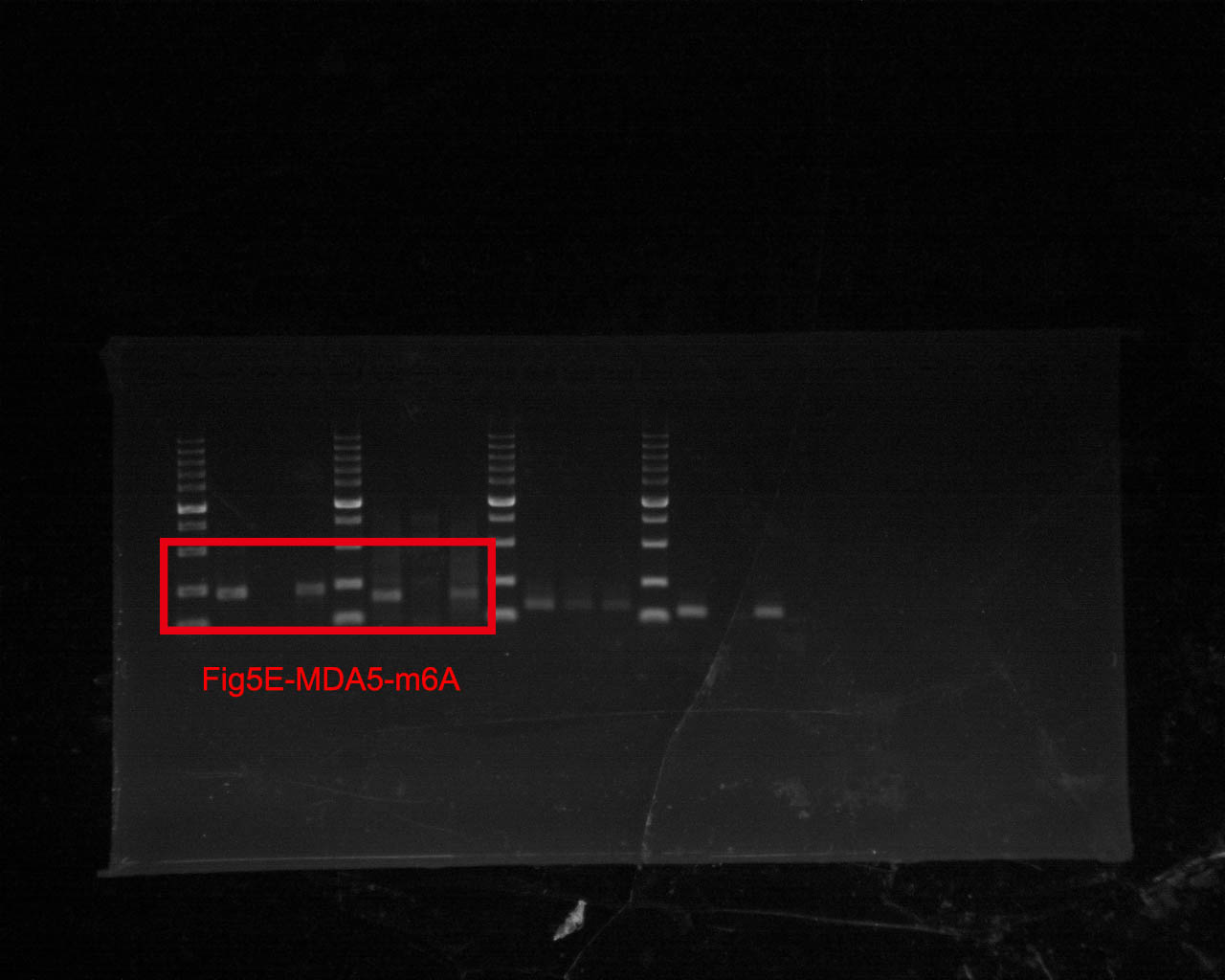

Supplement: Figure 5—source data 3. [file elife-94898-fig5-data3.zip › Fig5E-MDA5-m6A.jpg]

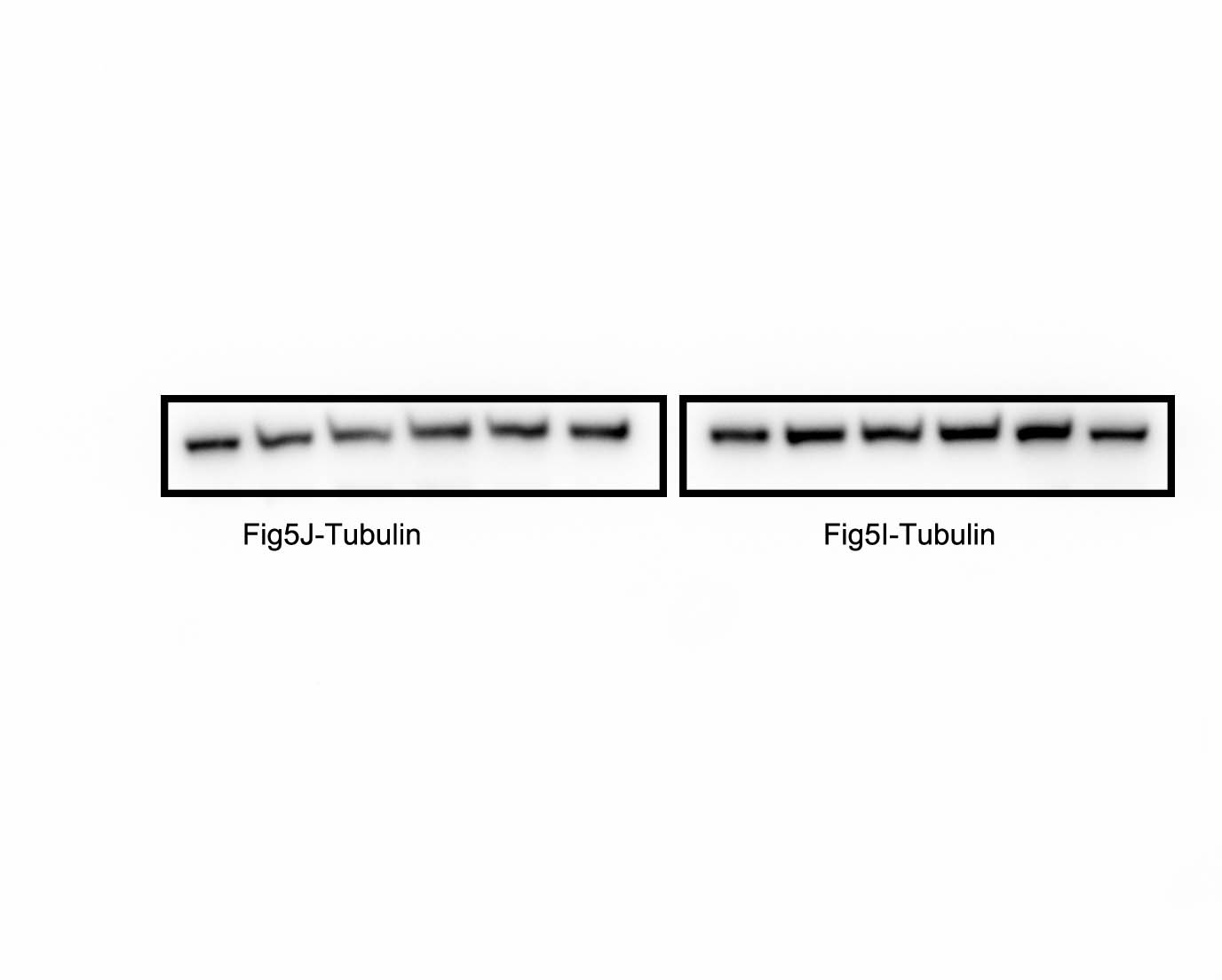

Supplement: Figure 5—source data 3. [file elife-94898-fig5-data3.zip › Fig5I+5J-Tubulin.jpg]

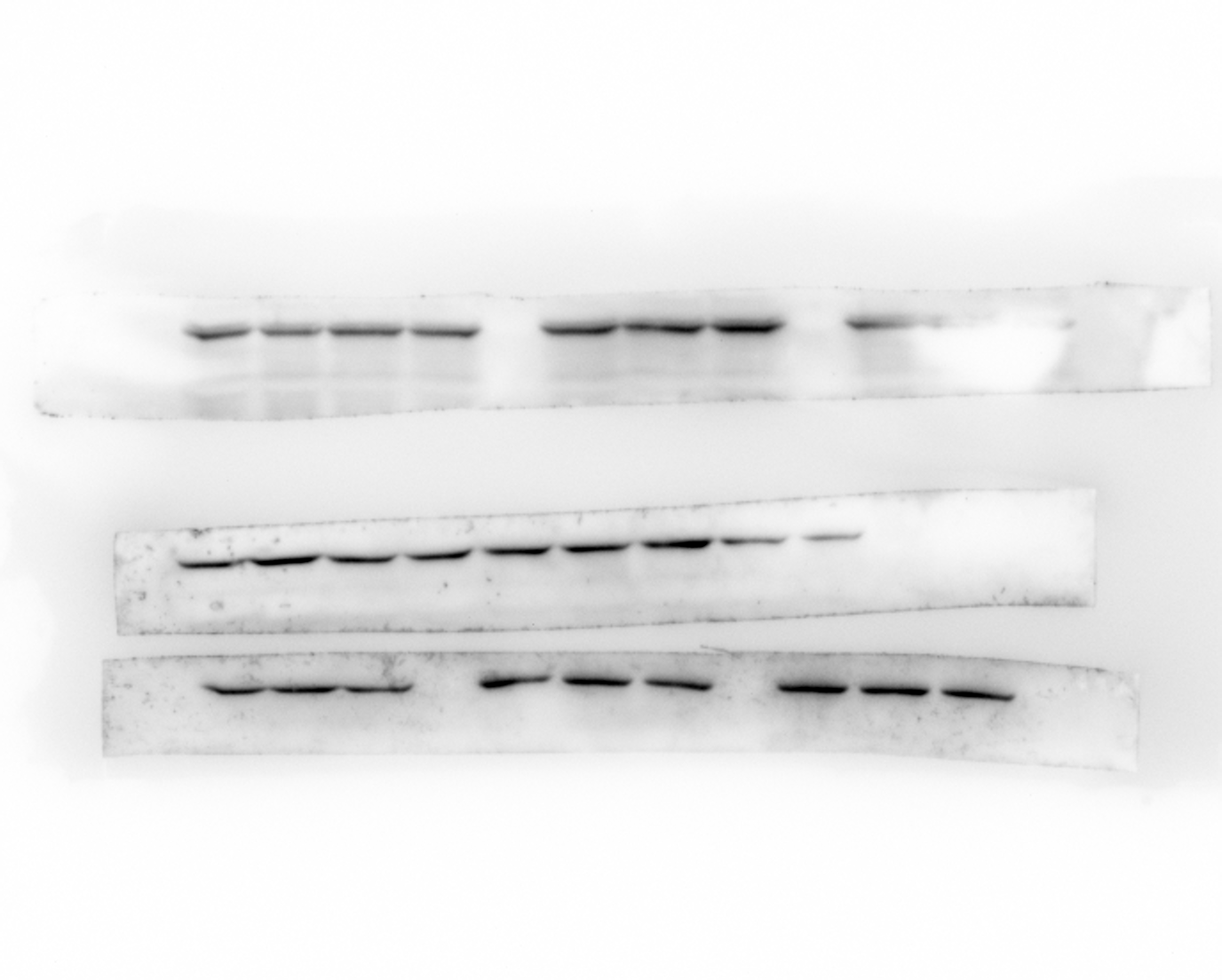

Supplement: Figure 6—source data 2. [file elife-94898-fig6-data2.zip › Fig6H-Tubulin.Tif]

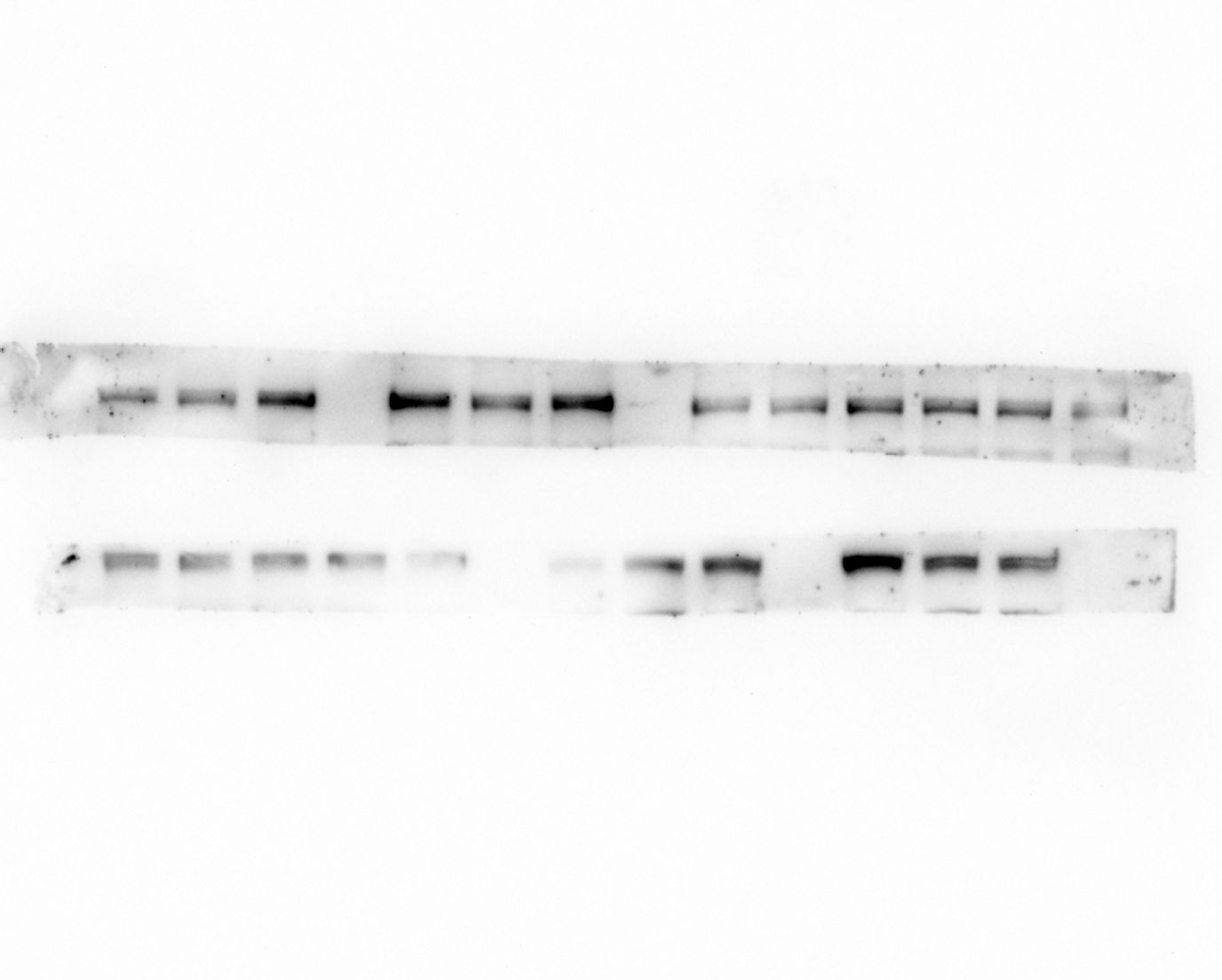

Supplement: Figure 6—source data 2. [file elife-94898-fig6-data2.zip › Fig6J-MDA5.Tif]

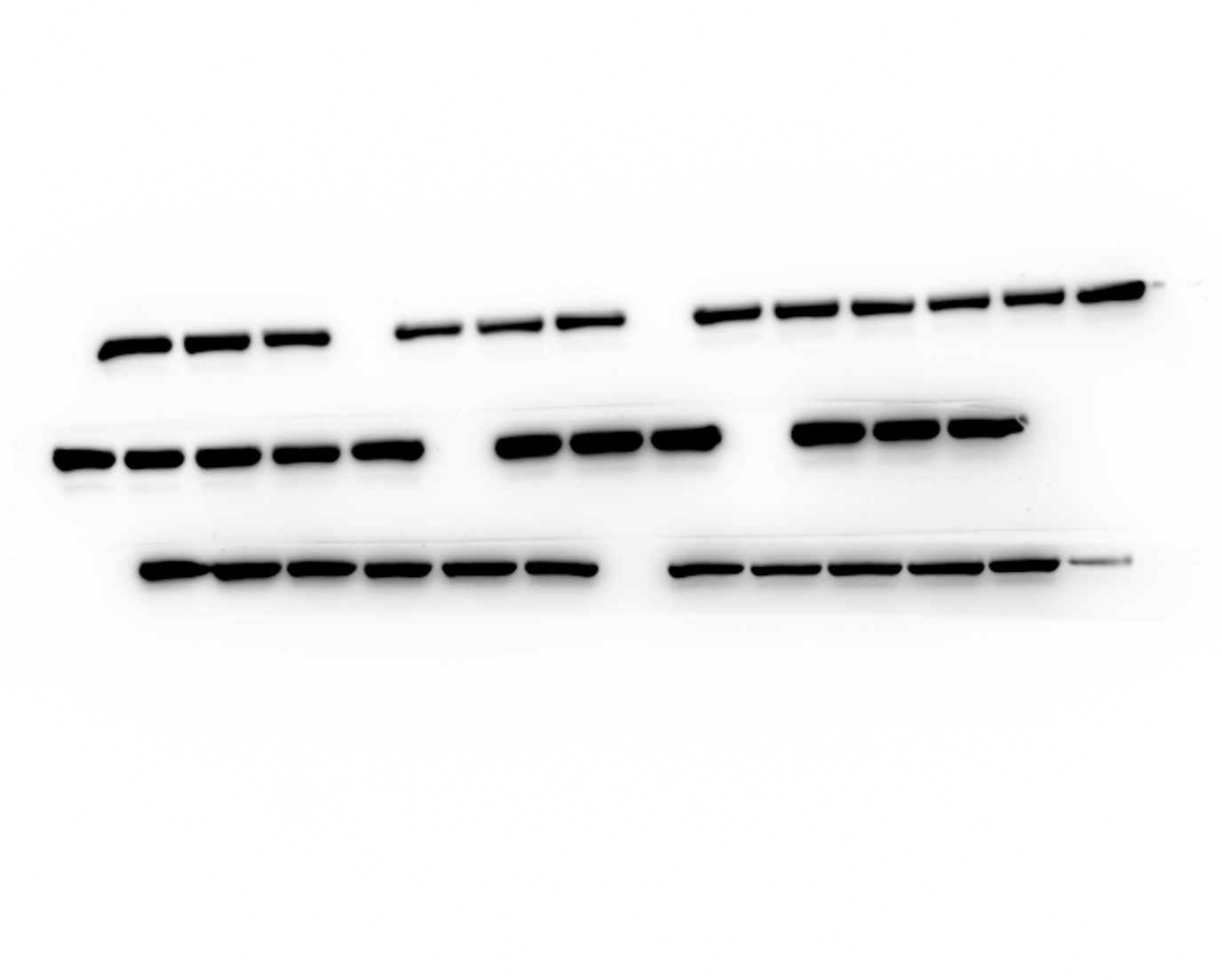

Supplement: Figure 6—source data 2. [file elife-94898-fig6-data2.zip › Fig6J-Tubulin.Tif]

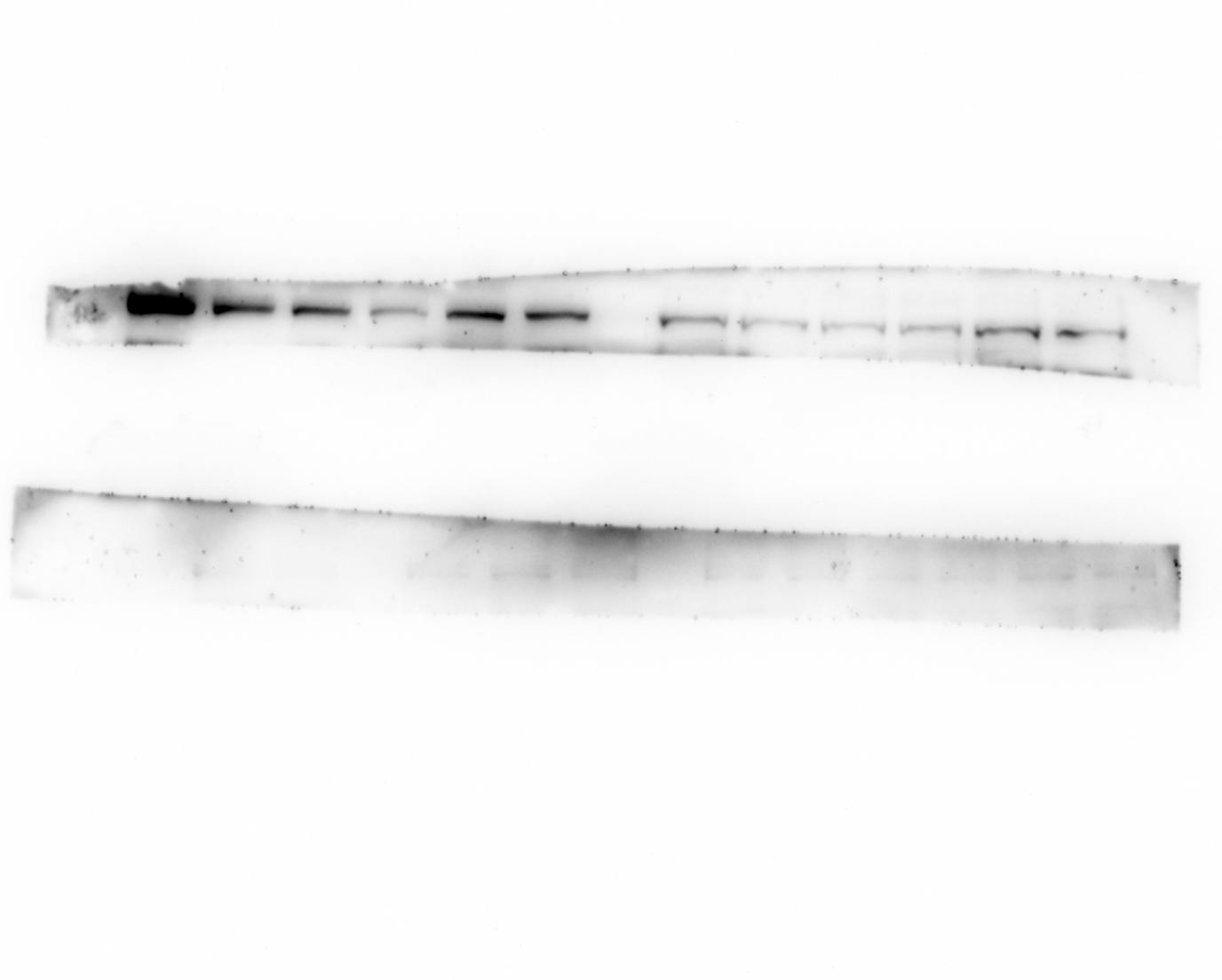

Supplement: Figure 6—source data 2. [file elife-94898-fig6-data2.zip › Fig6L-MDA5.Tif]

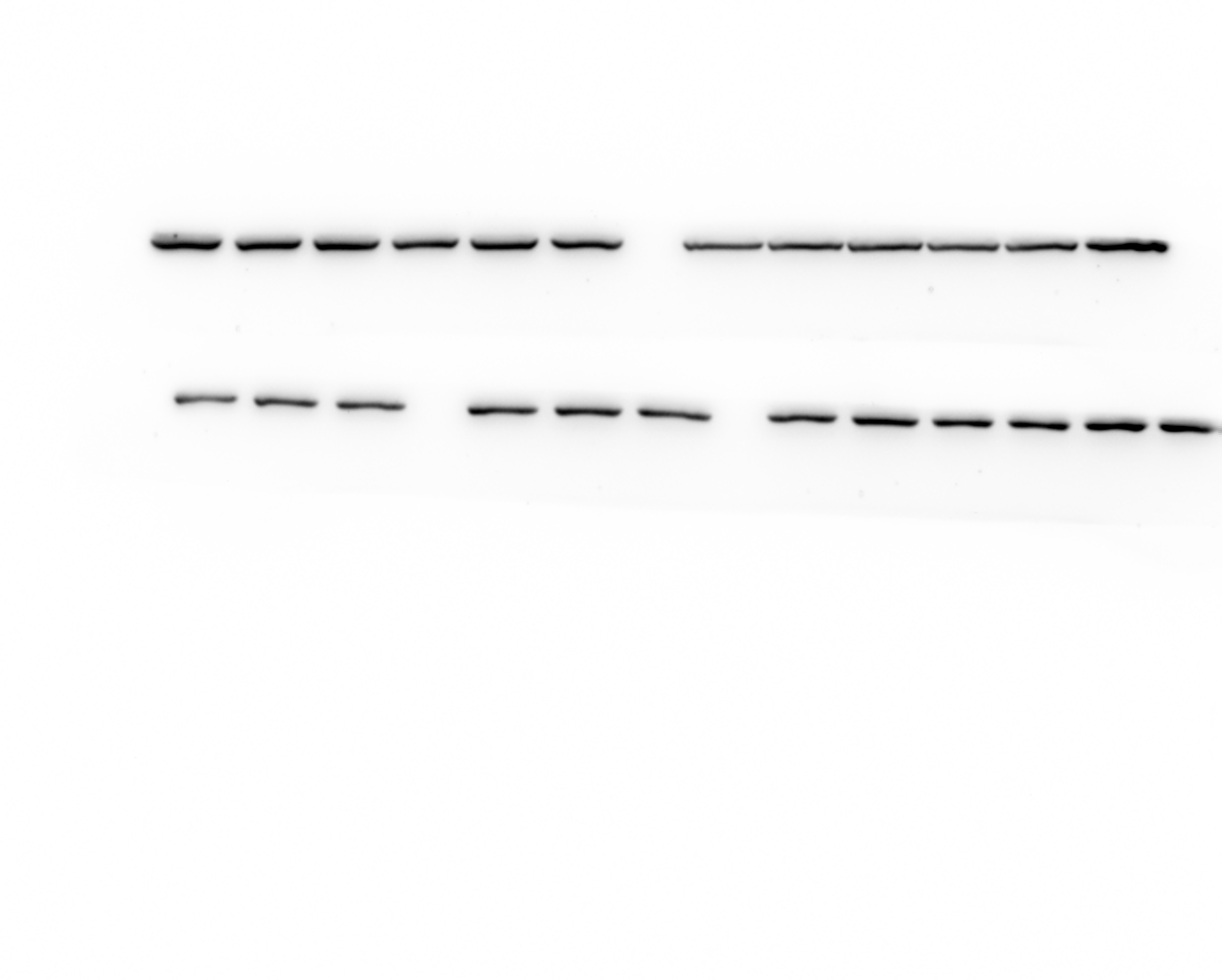

Supplement: Figure 6—source data 2. [file elife-94898-fig6-data2.zip › Fig6L-Tubulin.Tif]

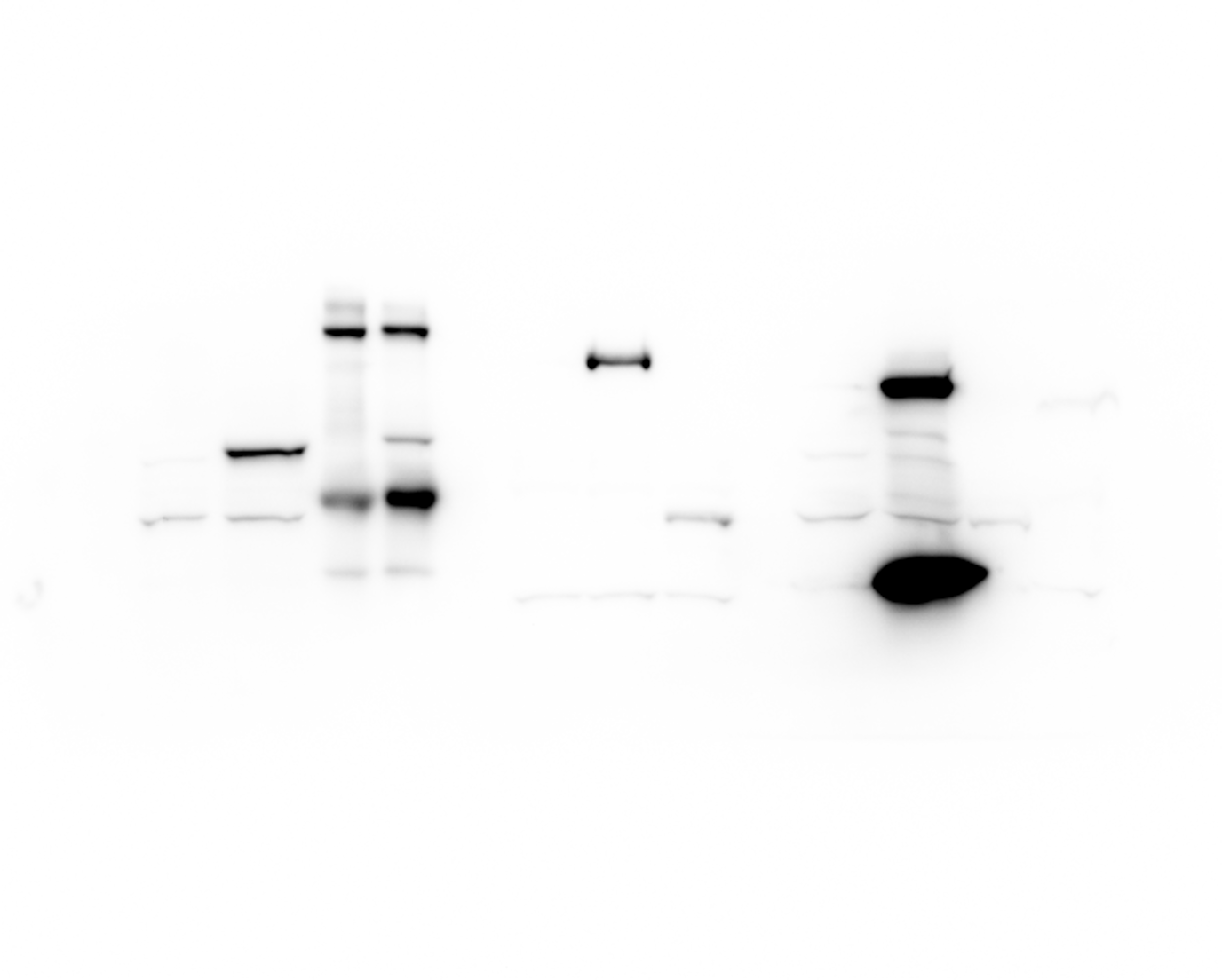

Supplement: Figure 6—source data 2. [file elife-94898-fig6-data2.zip › Fig6B-Flag.Tif]

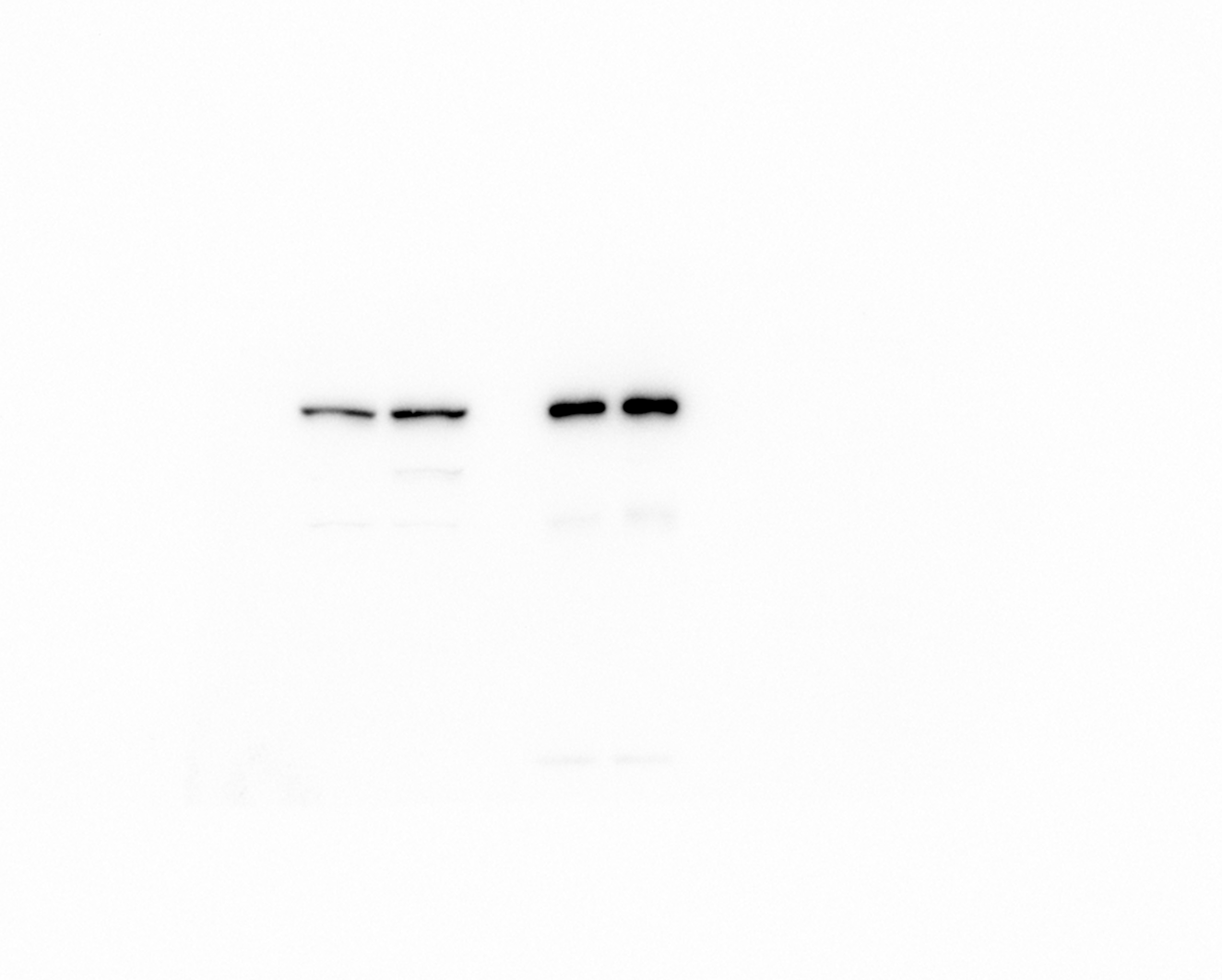

Supplement: Figure 6—source data 2. [file elife-94898-fig6-data2.zip › Fig6B-Myc.Tif]

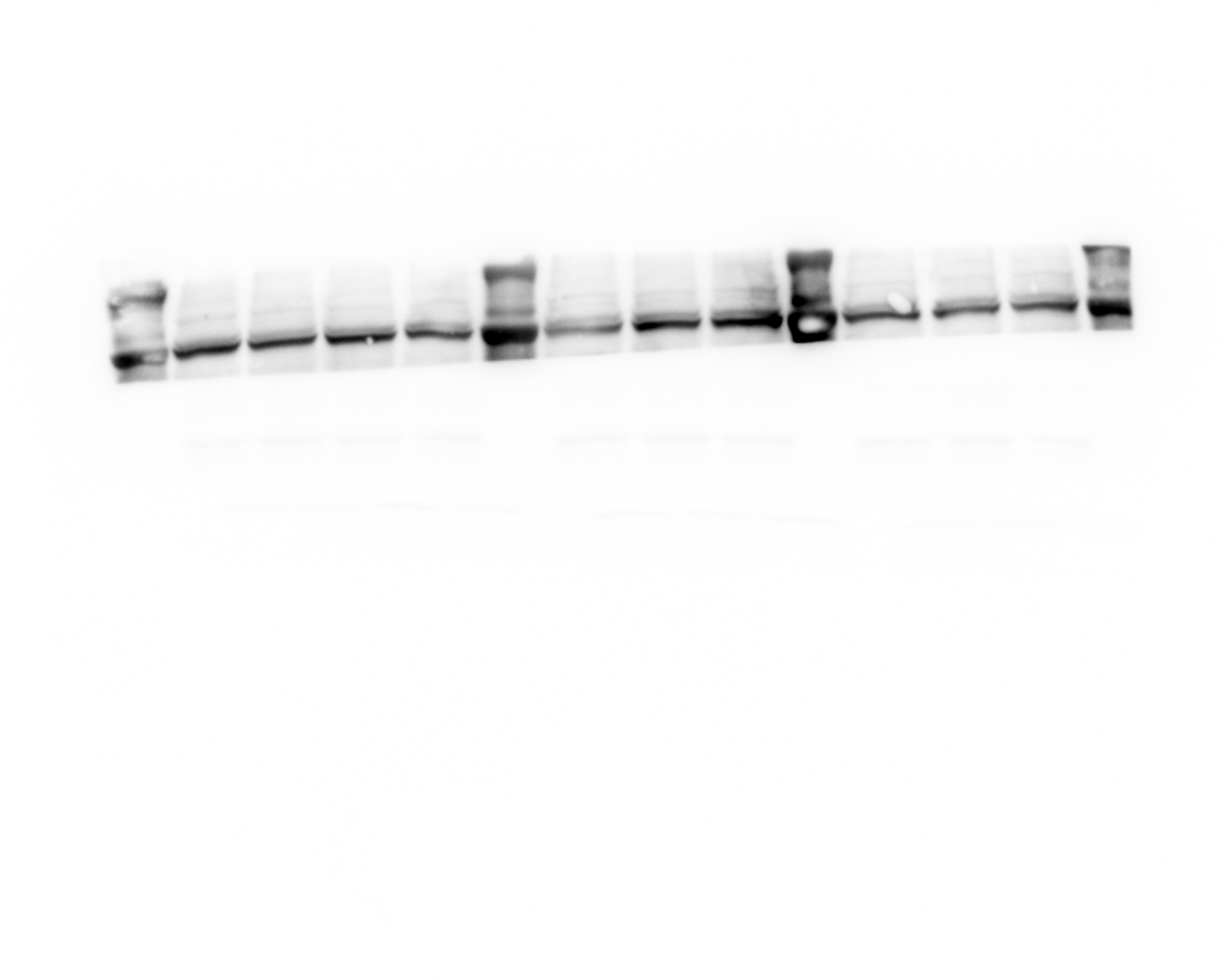

Supplement: Figure 6—source data 2. [file elife-94898-fig6-data2.zip › Fig6G-MDA5.Tif]

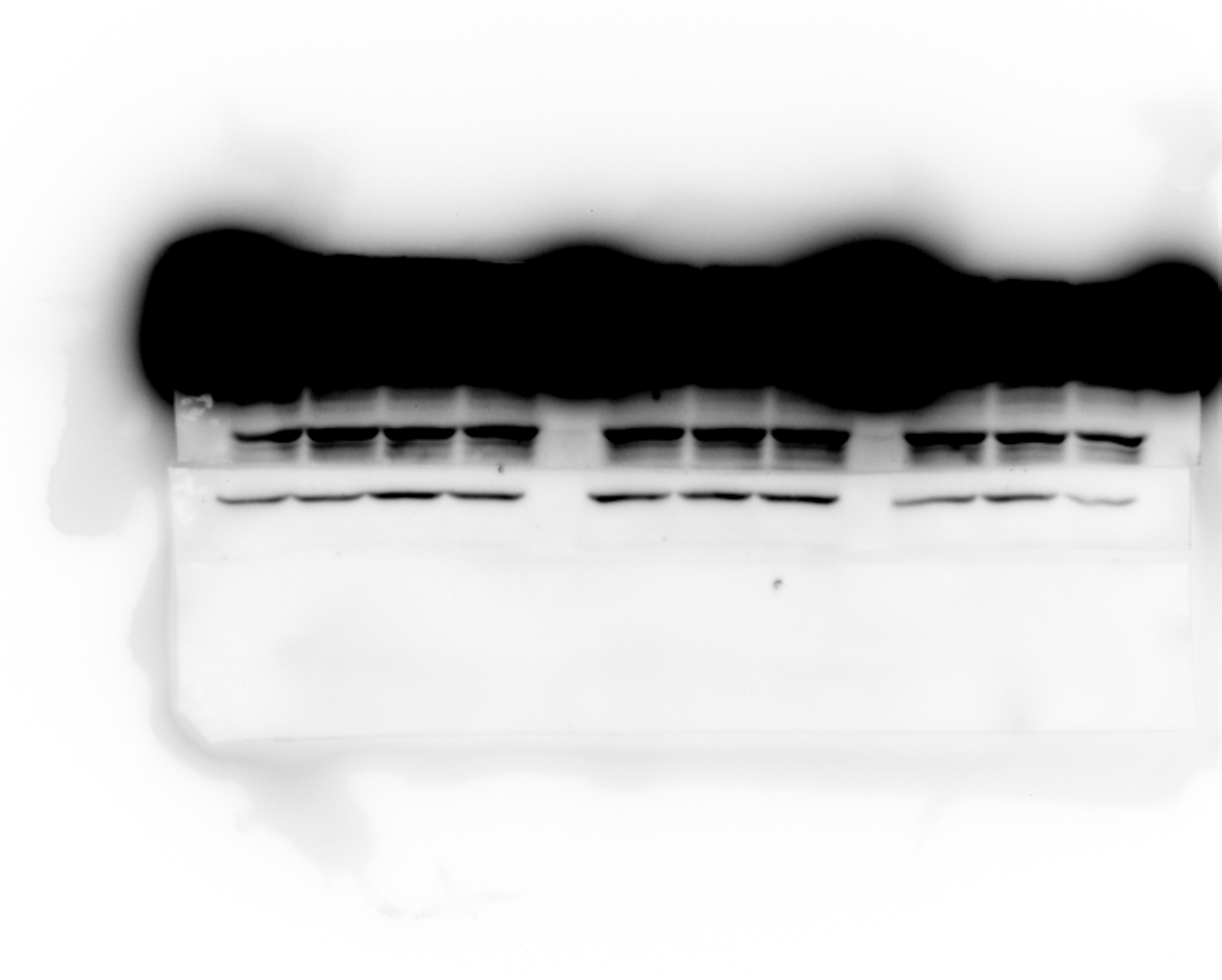

Supplement: Figure 6—source data 2. [file elife-94898-fig6-data2.zip › Fig6G-Tubulin.Tif]

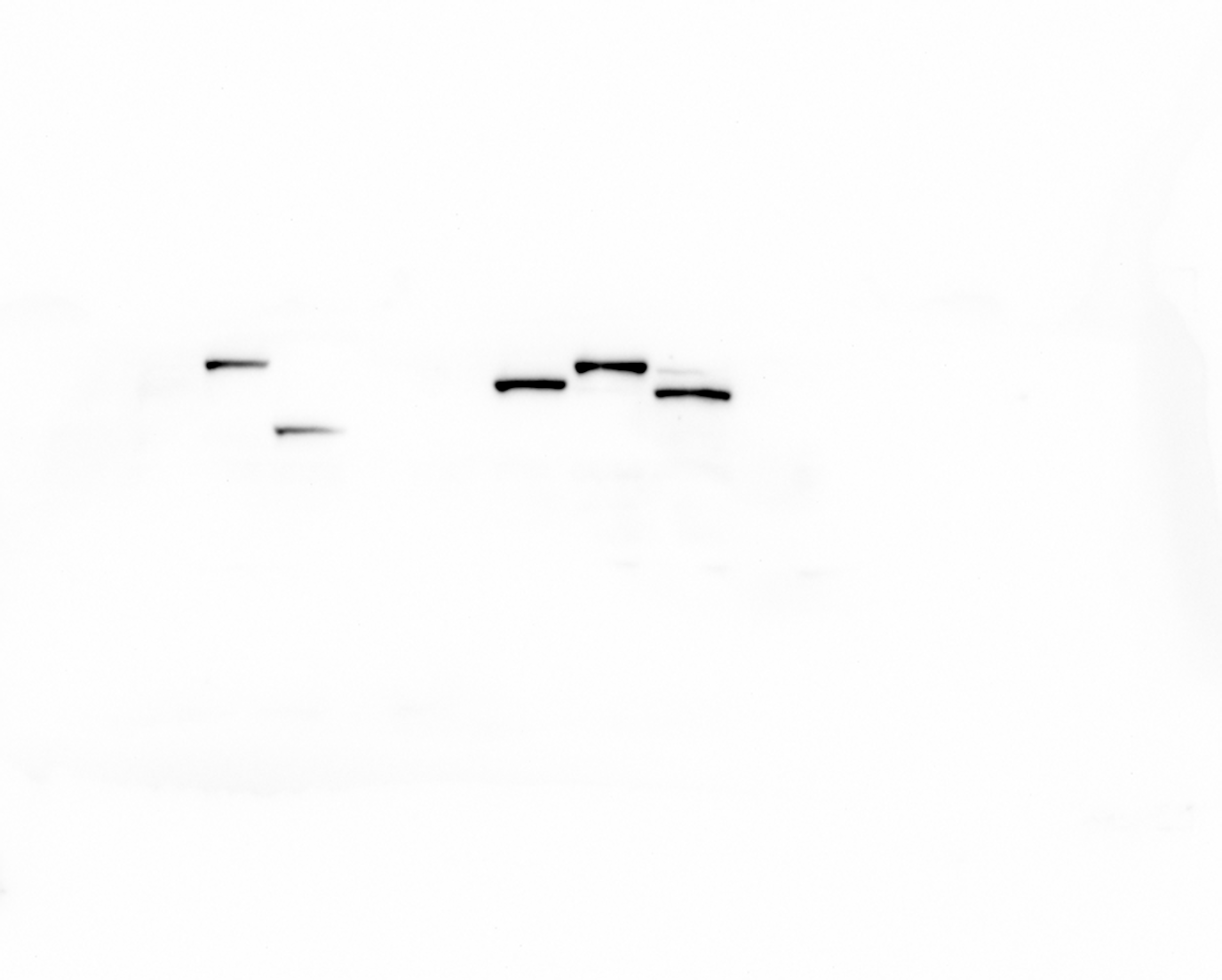

Supplement: Figure 6—source data 2. [file elife-94898-fig6-data2.zip › Fig6H-Flag.Tif]

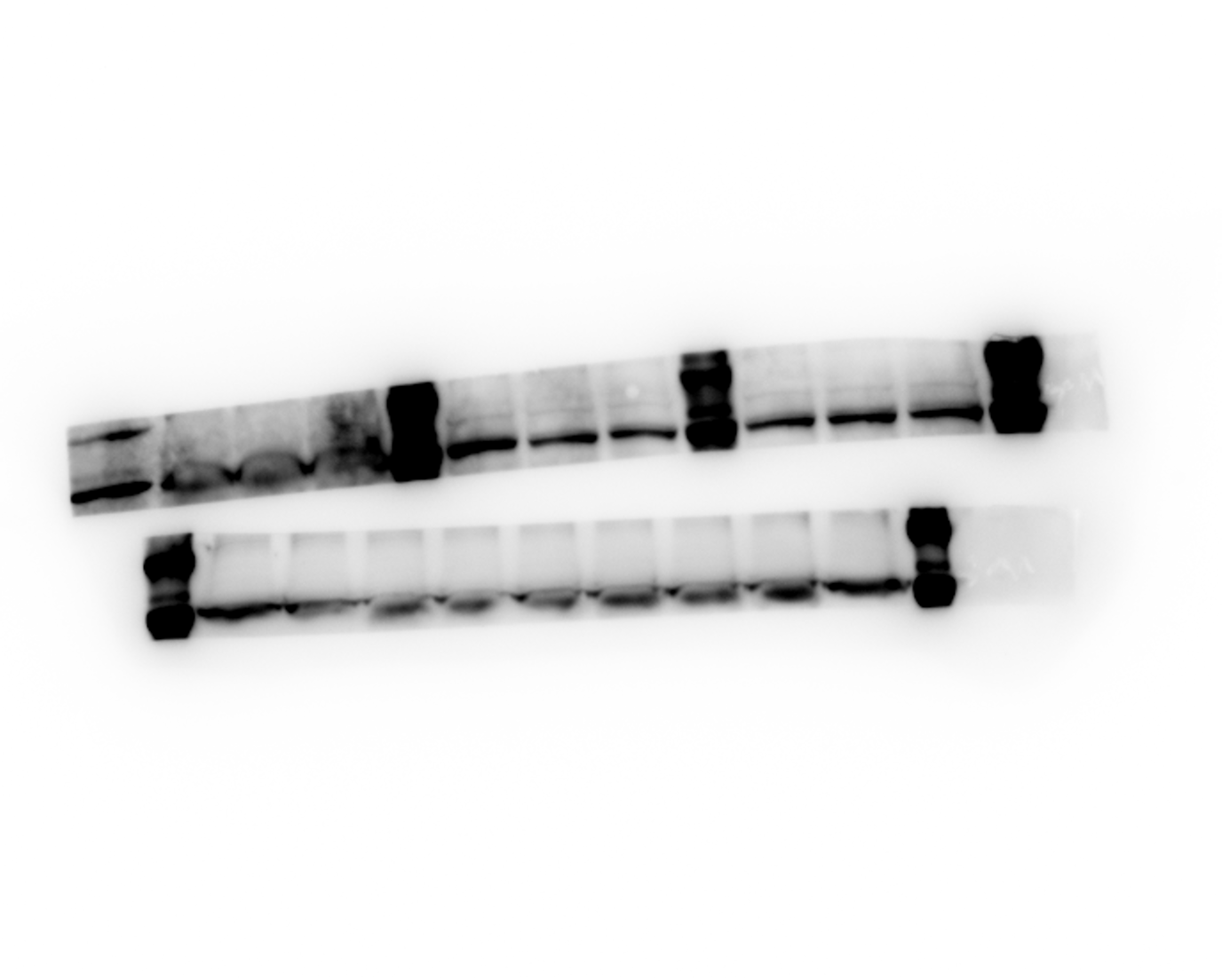

Supplement: Figure 6—source data 2. [file elife-94898-fig6-data2.zip › Fig6H-MDA5.Tif]

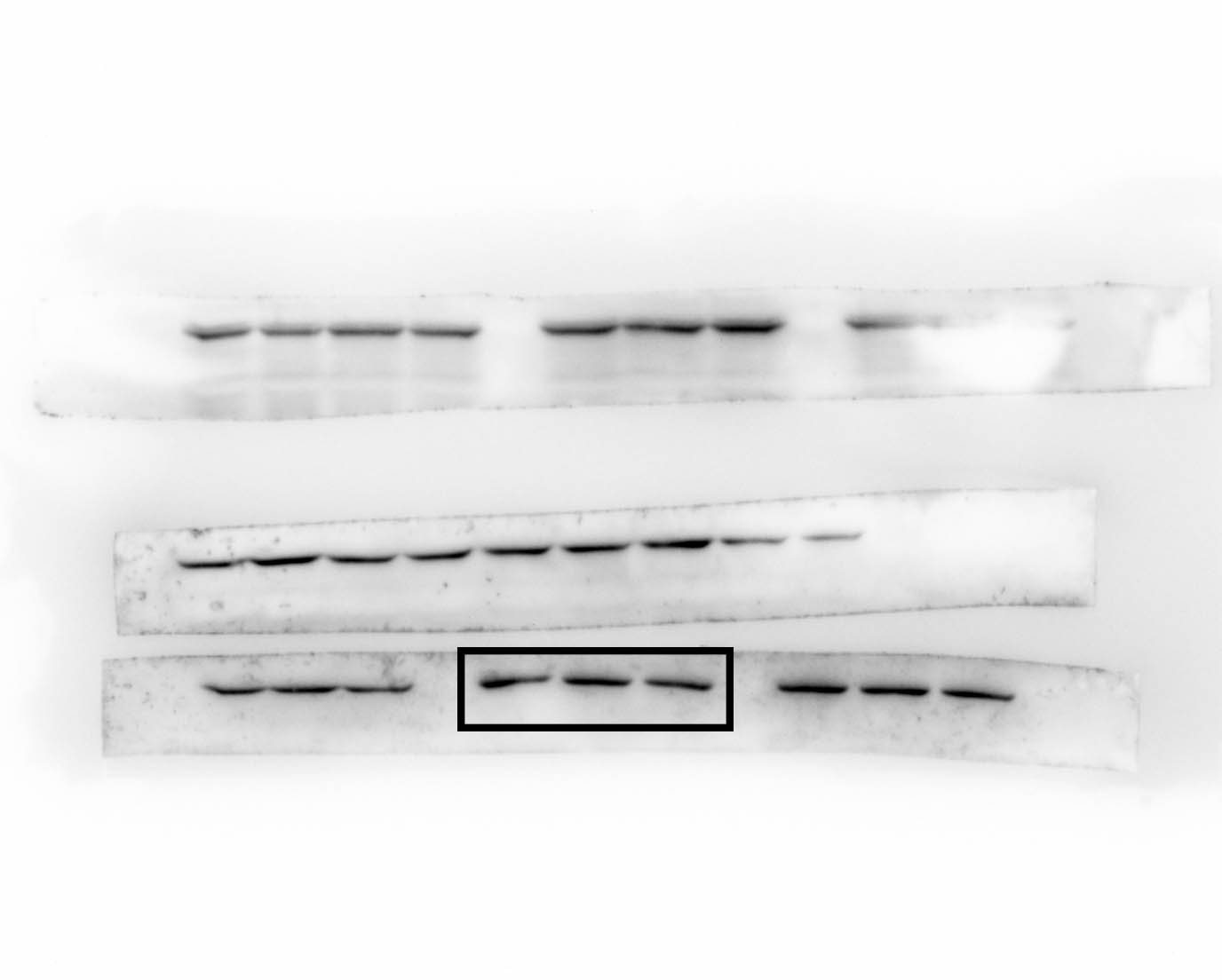

Supplement: Figure 6—source data 3. [file elife-94898-fig6-data3.zip › Fig6H-Tubulin.jpg]

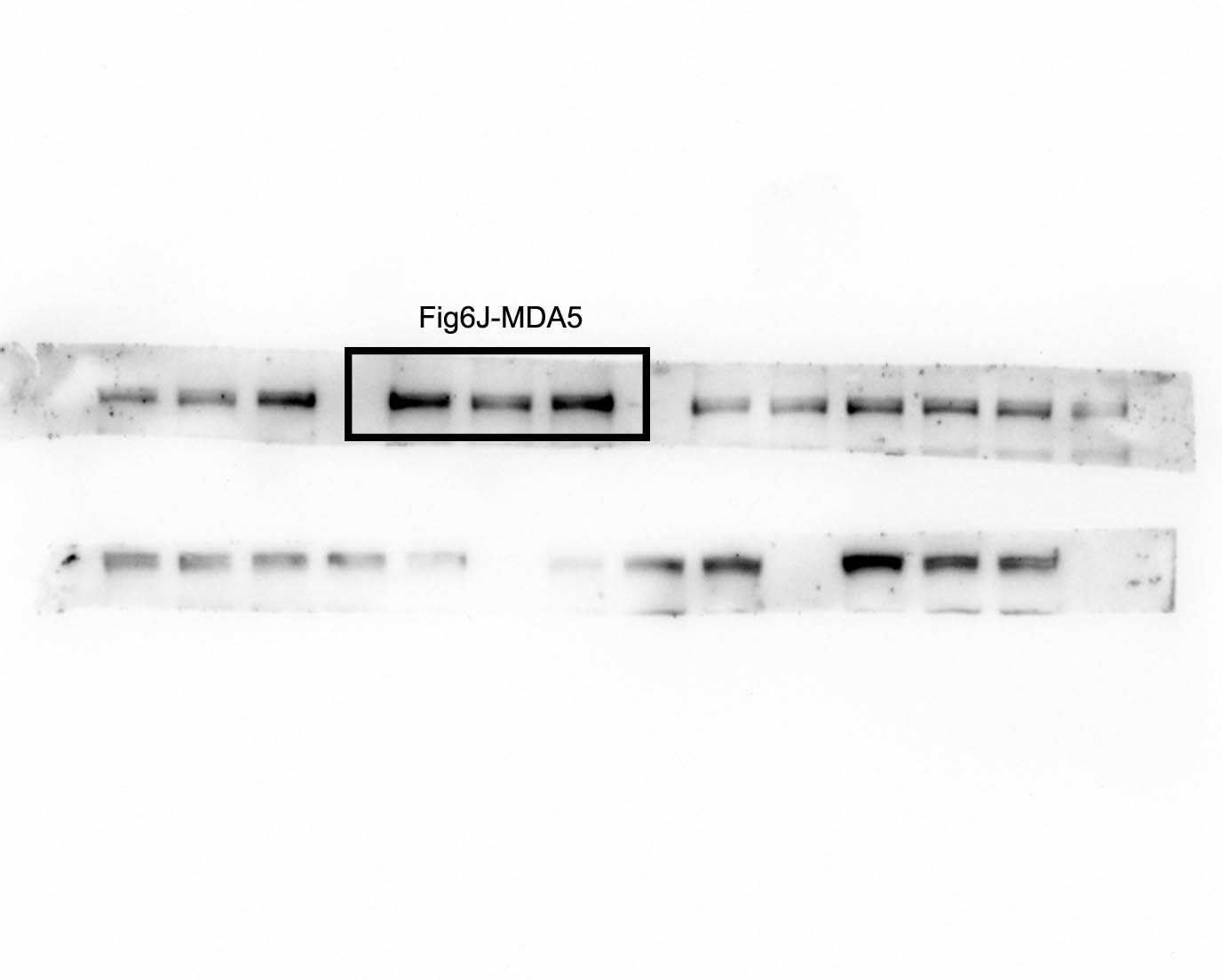

Supplement: Figure 6—source data 3. [file elife-94898-fig6-data3.zip › Fig6J-MDA5.jpg]

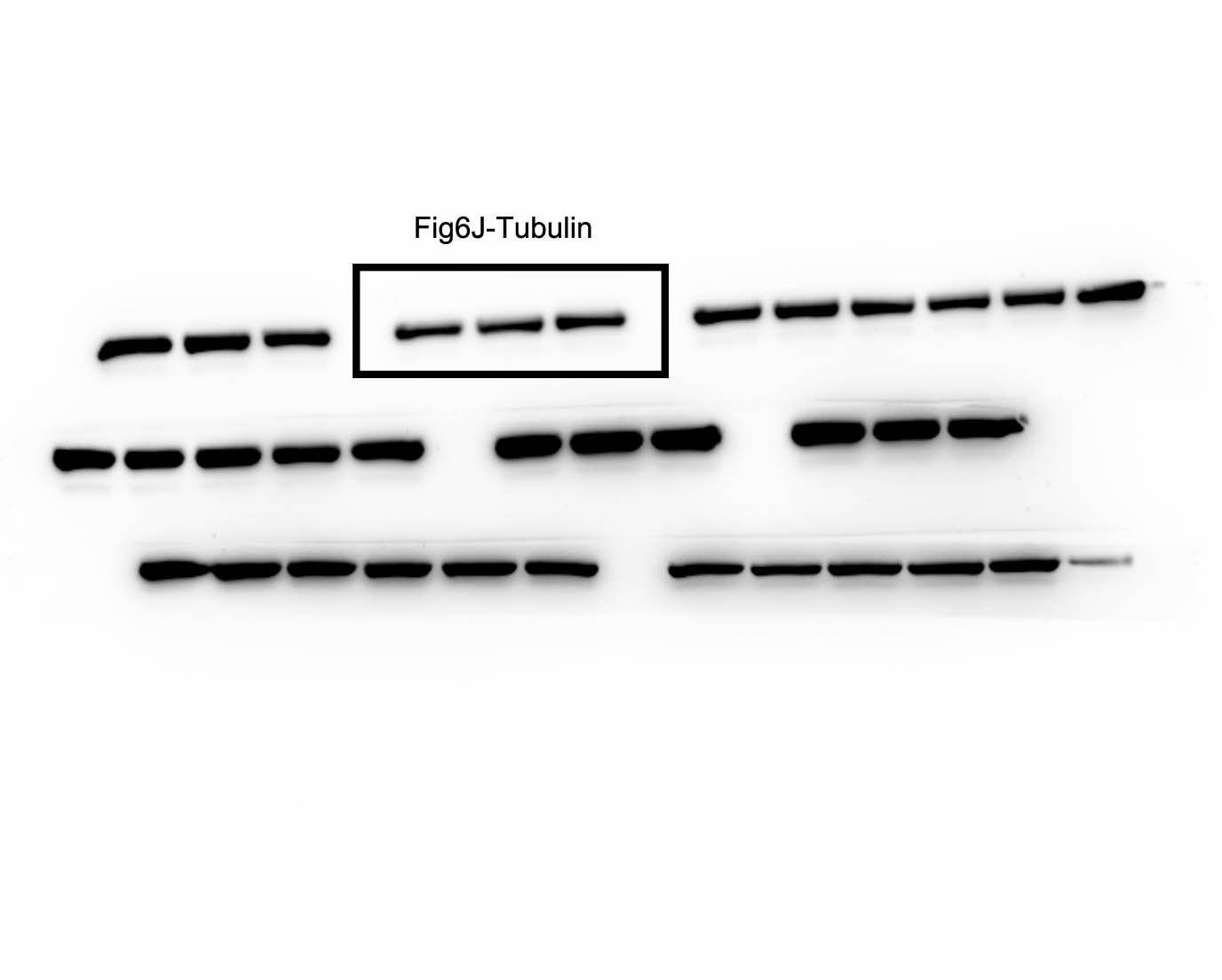

Supplement: Figure 6—source data 3. [file elife-94898-fig6-data3.zip › Fig6J-Tubulin.jpg]

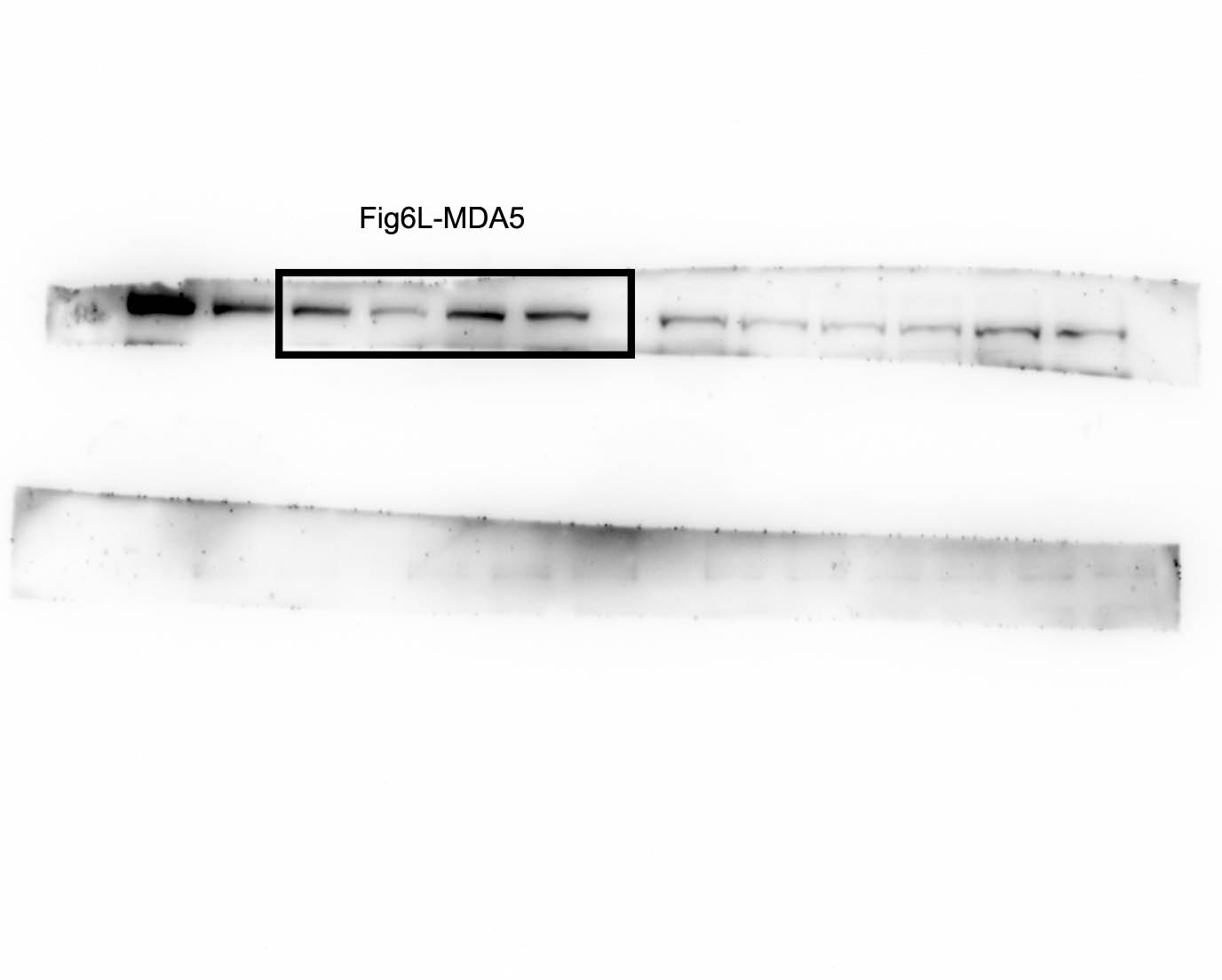

Supplement: Figure 6—source data 3. [file elife-94898-fig6-data3.zip › Fig6L-MDA5.jpg]

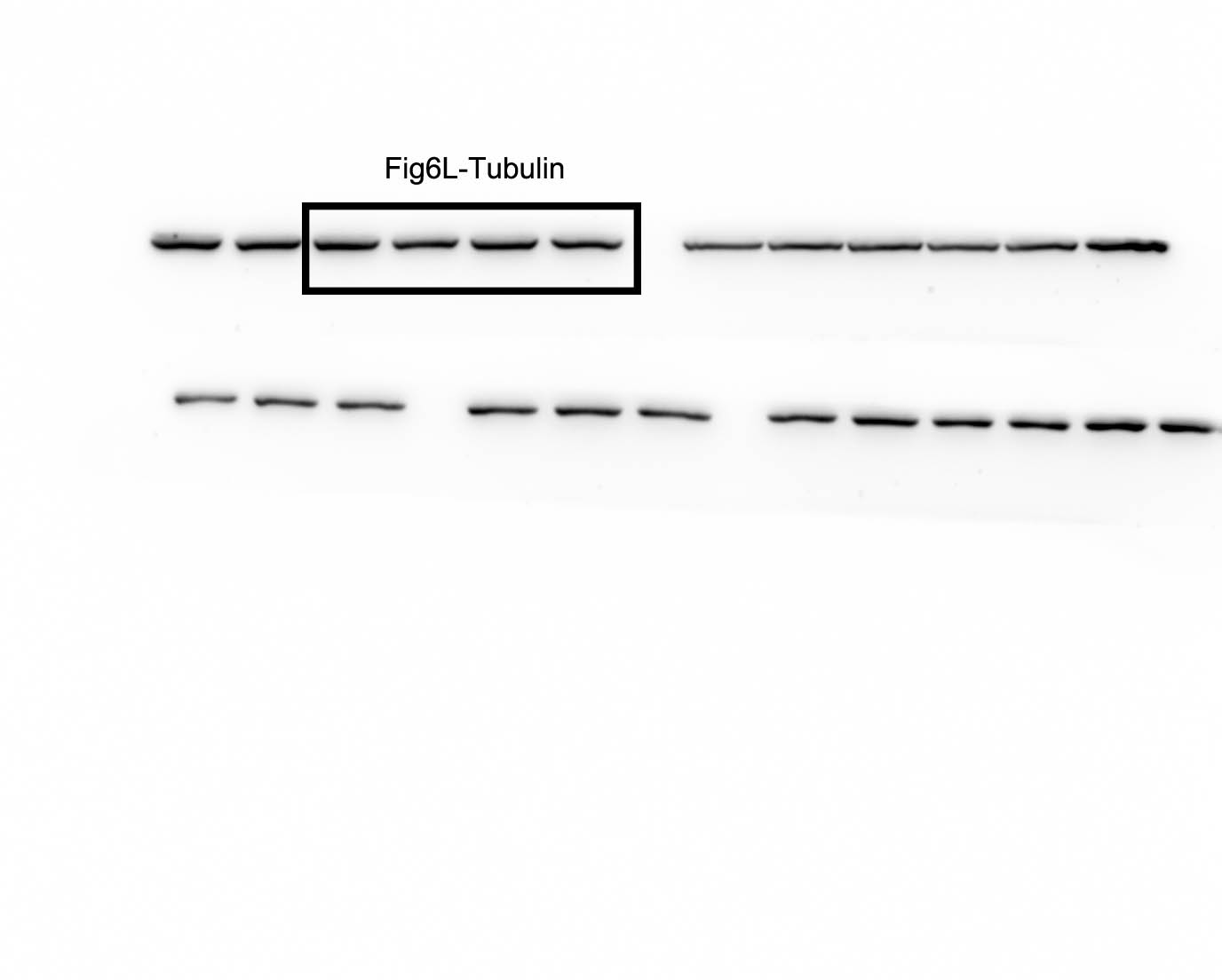

Supplement: Figure 6—source data 3. [file elife-94898-fig6-data3.zip › Fig6L-Tubulin.jpg]

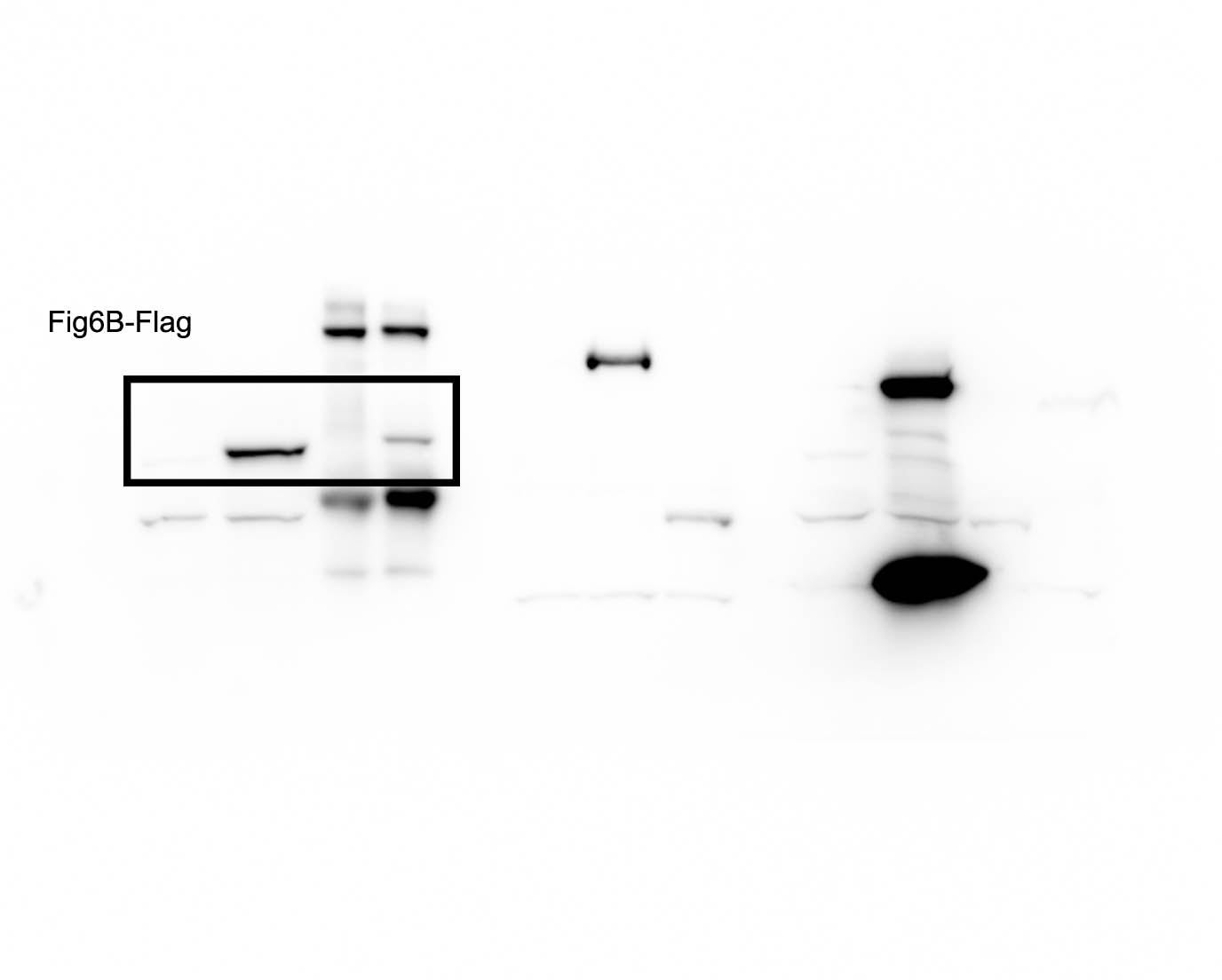

Supplement: Figure 6—source data 3. [file elife-94898-fig6-data3.zip › Fig6B-Flag.jpg]

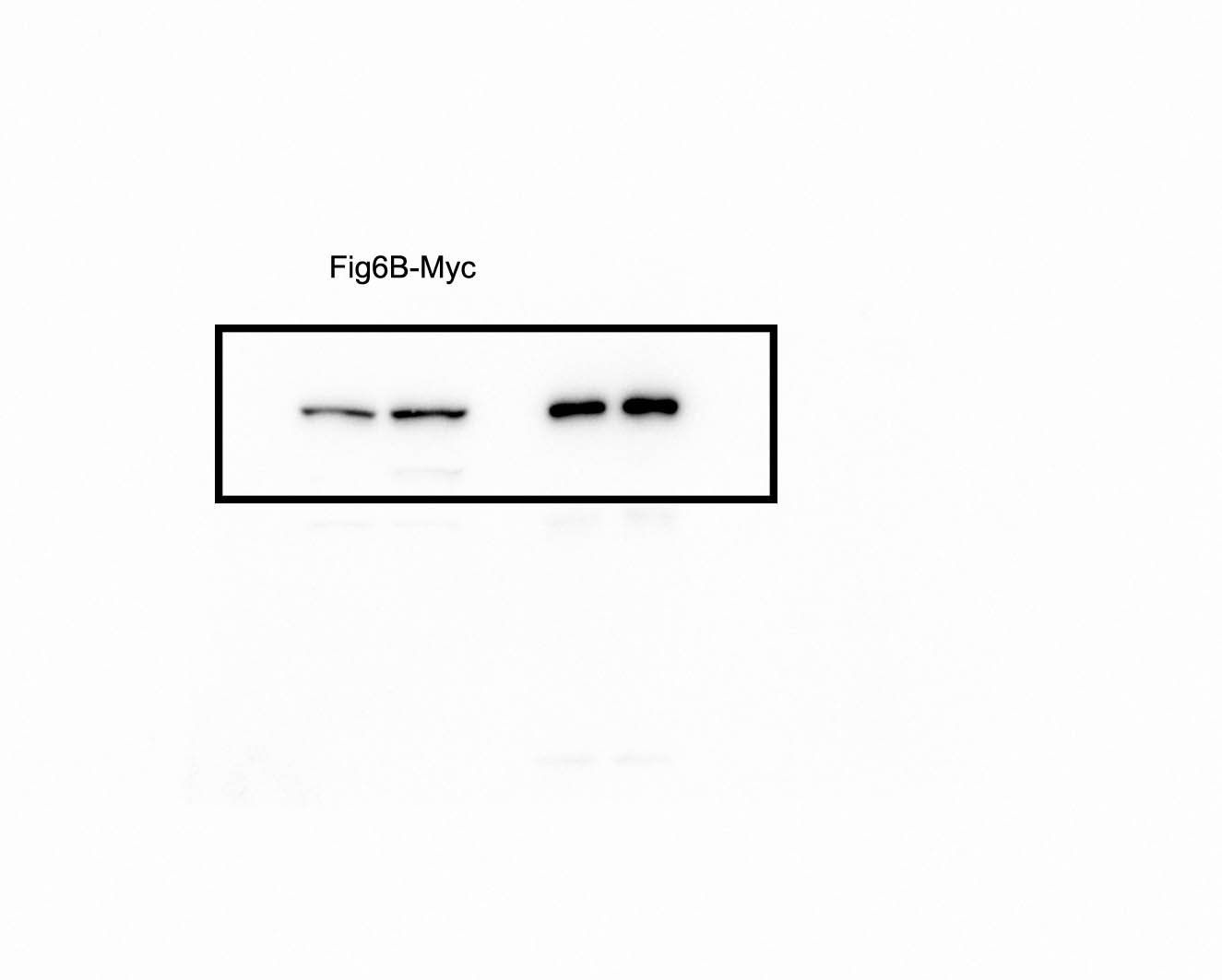

Supplement: Figure 6—source data 3. [file elife-94898-fig6-data3.zip › Fig6B-Myc.jpg]

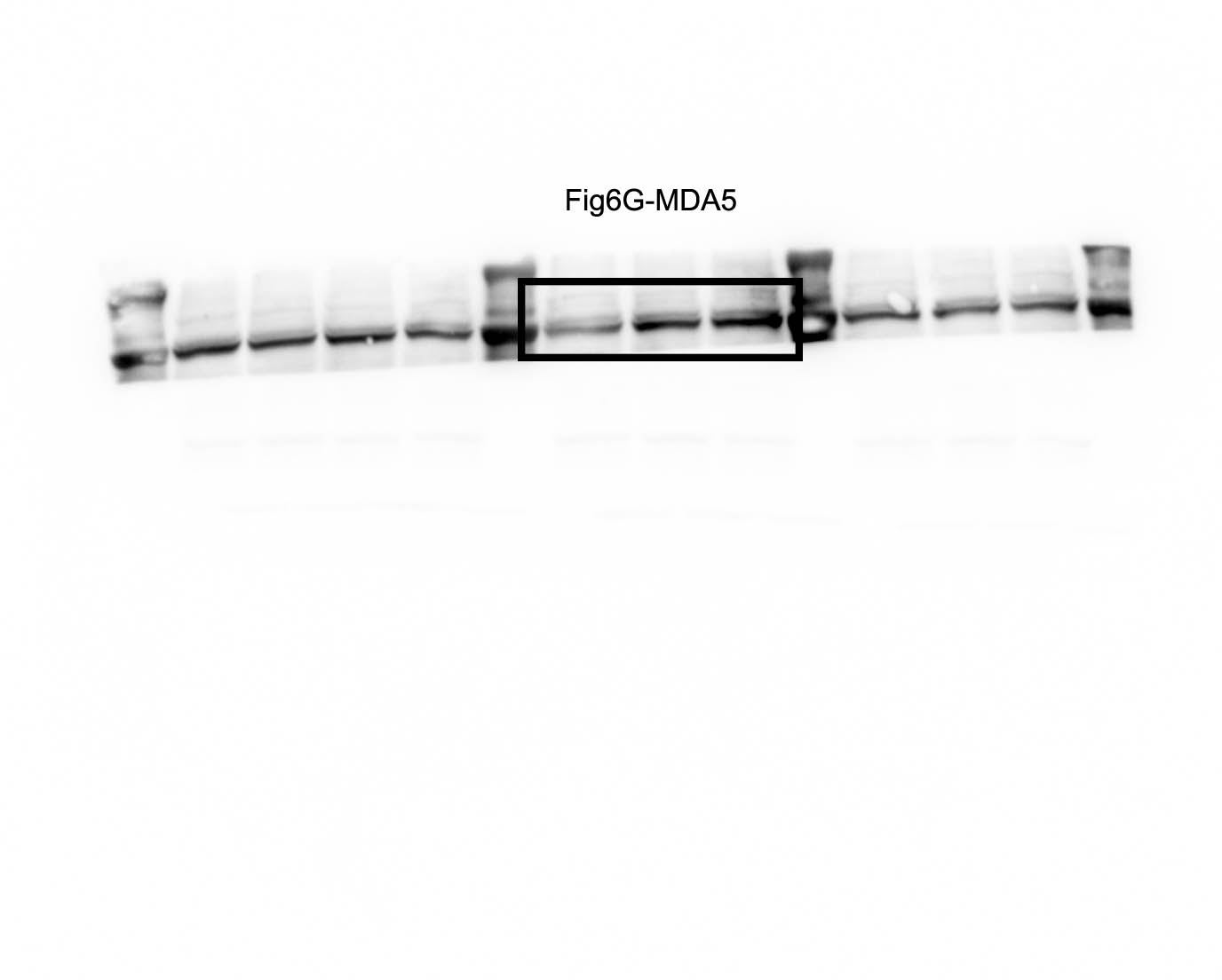

Supplement: Figure 6—source data 3. [file elife-94898-fig6-data3.zip › Fig6G-MDA5.jpg]

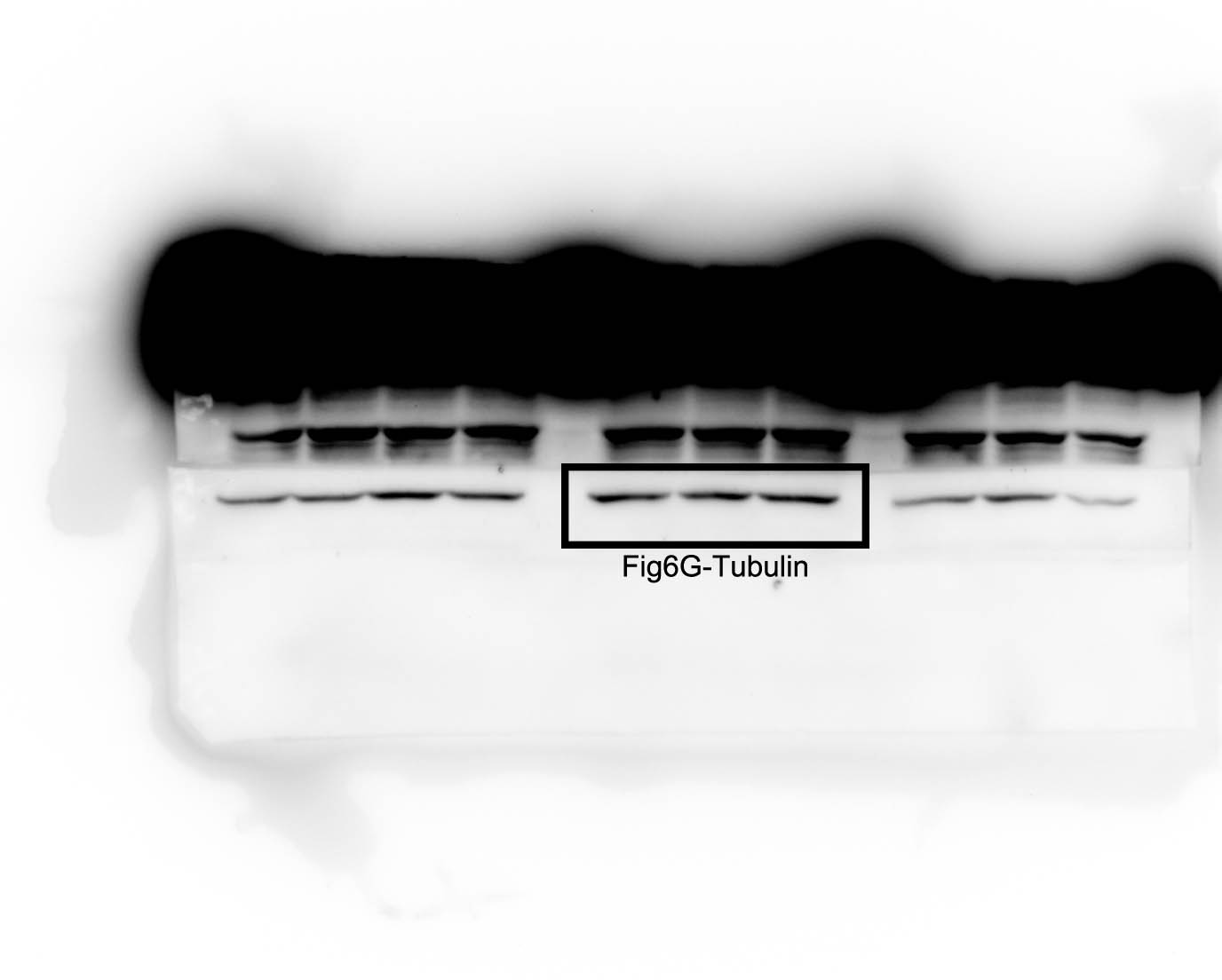

Supplement: Figure 6—source data 3. [file elife-94898-fig6-data3.zip › Fig6G-Tubulin.jpg]

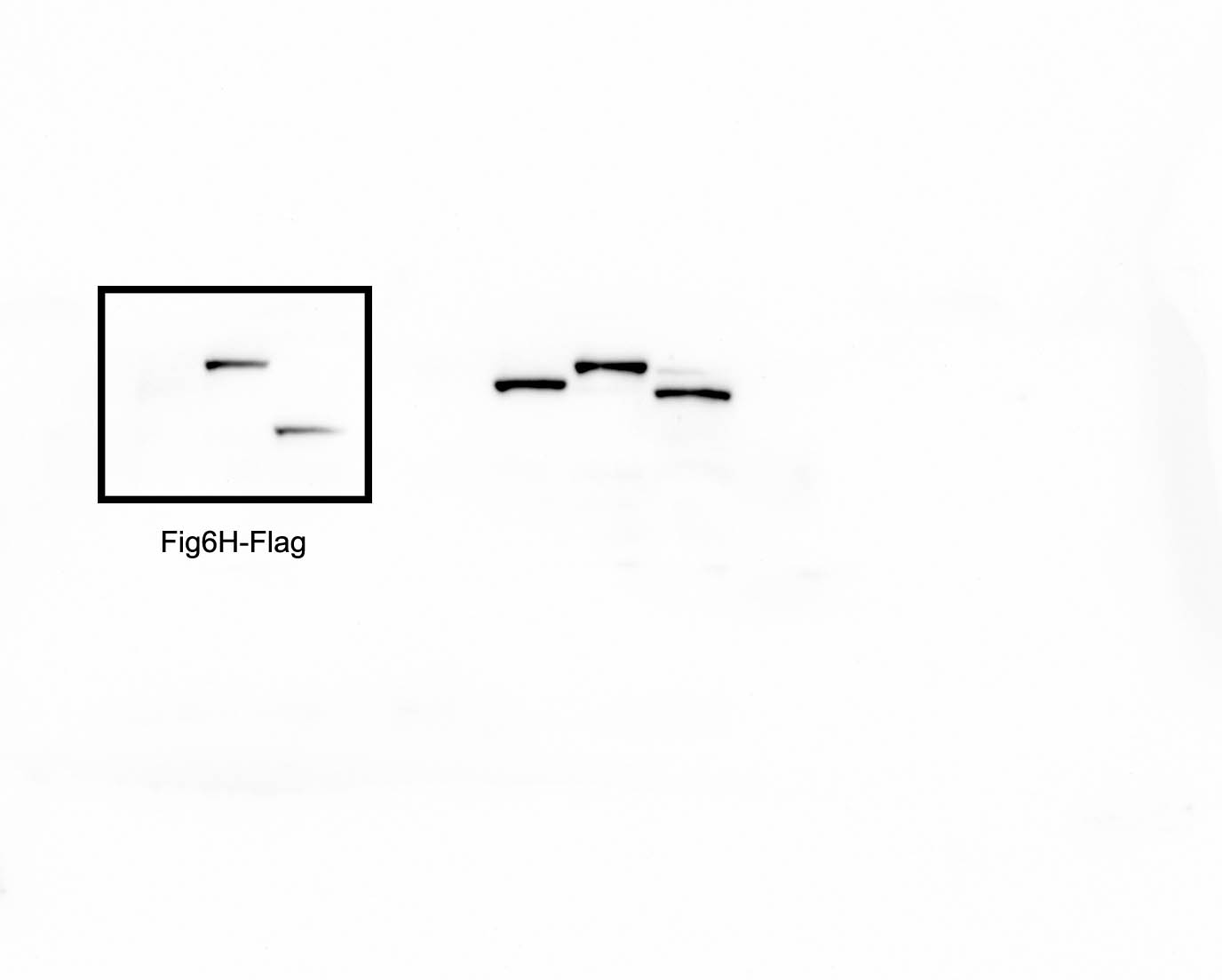

Supplement: Figure 6—source data 3. [file elife-94898-fig6-data3.zip › Fig6H-Flag.jpg]

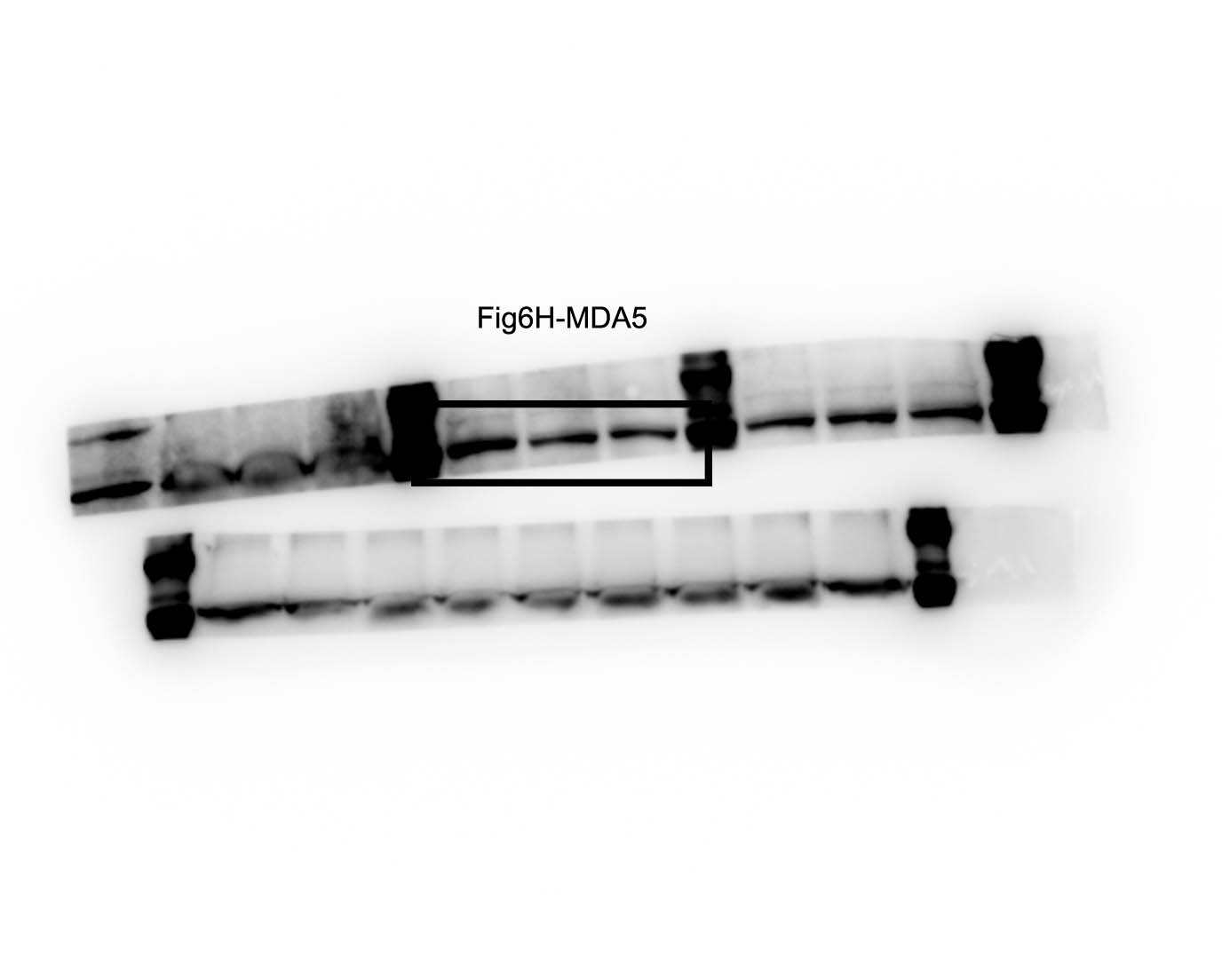

Supplement: Figure 6—source data 3. [file elife-94898-fig6-data3.zip › Fig6H-MDA5.jpg]

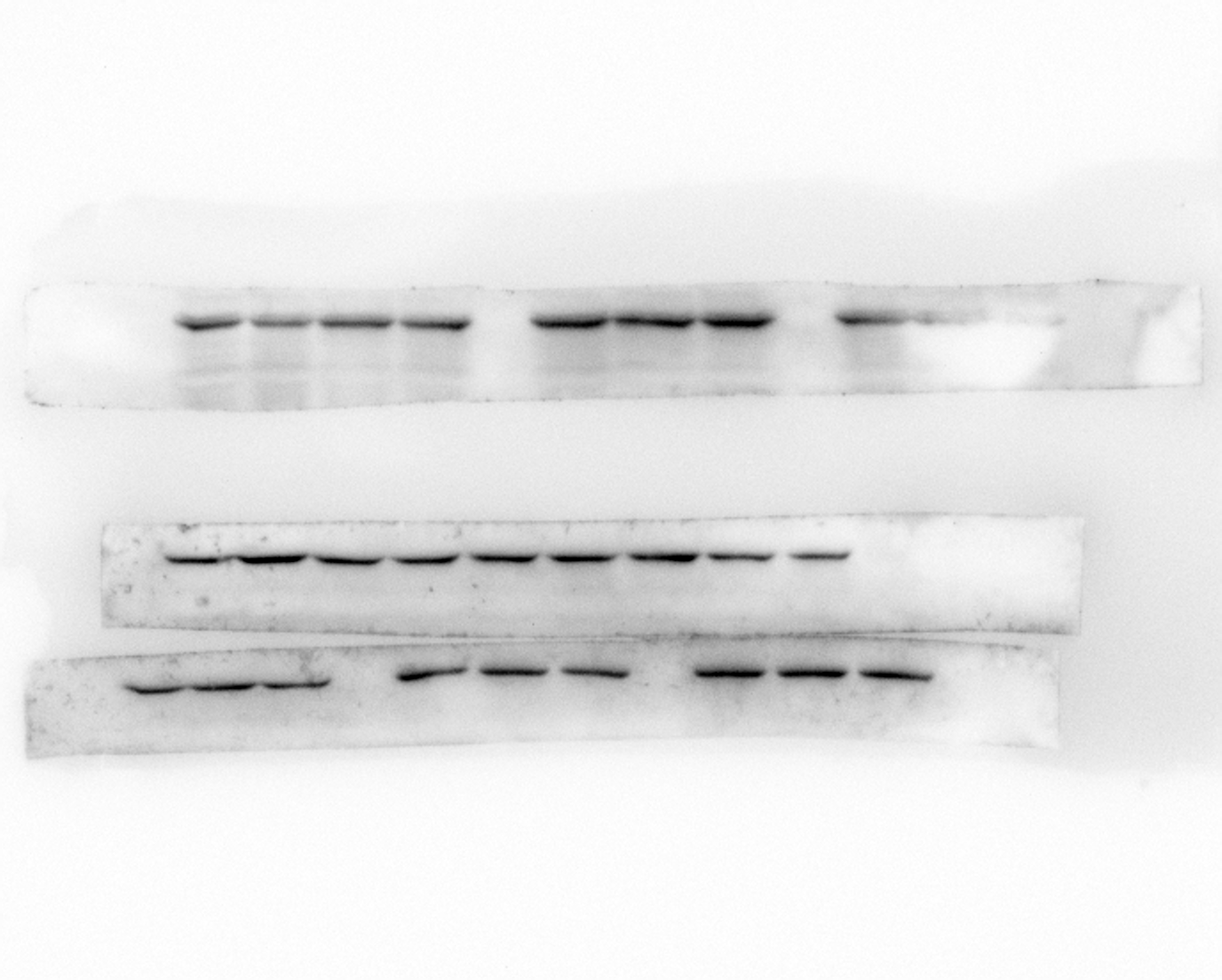

Supplement: Figure 6—figure supplement 1—source data 2. [file elife-94898-fig6-figsupp1-data2.zip › Figure 6-figure supplement 1D-Tubulin.Tif]

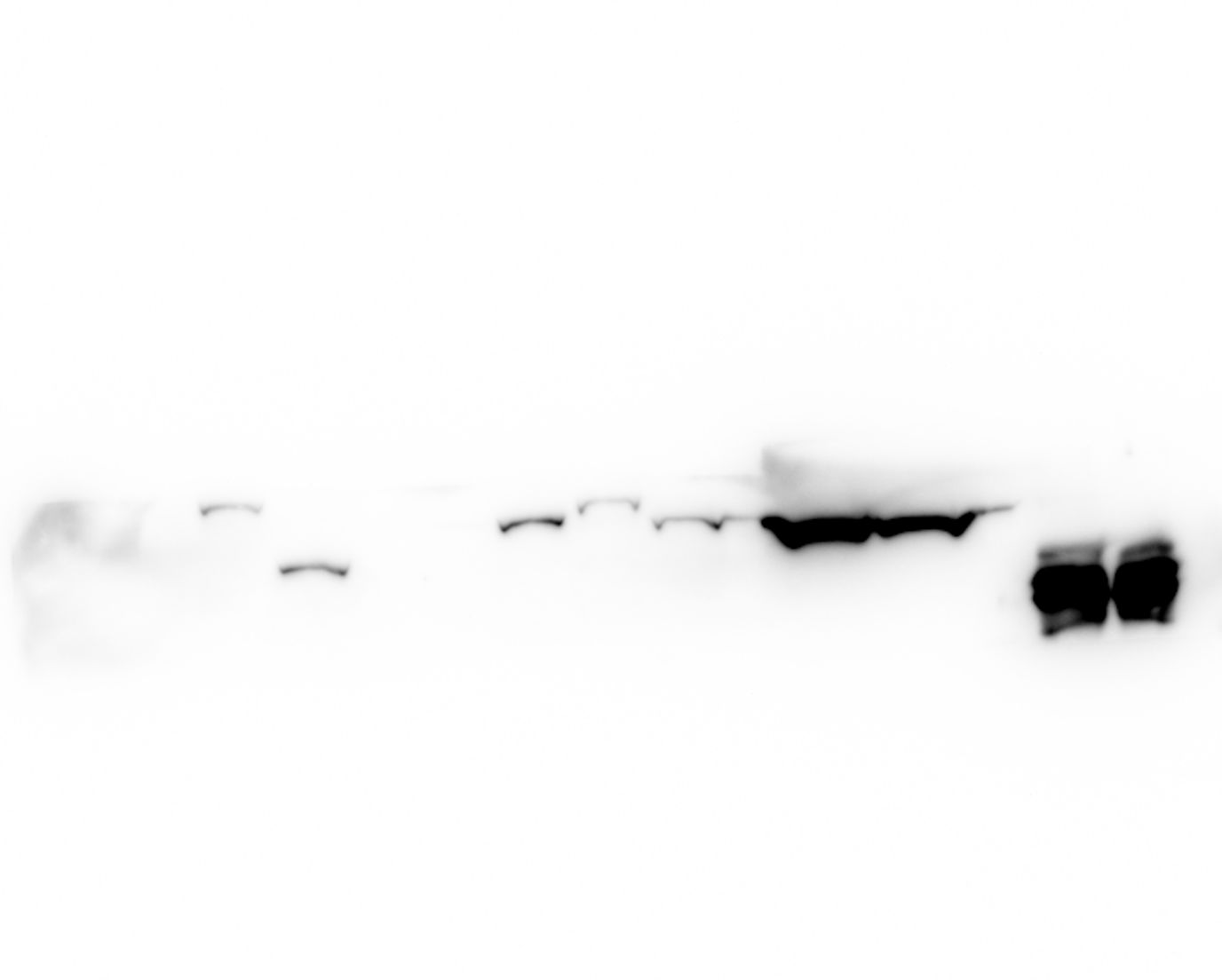

Supplement: Figure 6—figure supplement 1—source data 2. [file elife-94898-fig6-figsupp1-data2.zip › Figure 6-figure supplement 1D-Flag.Tif]

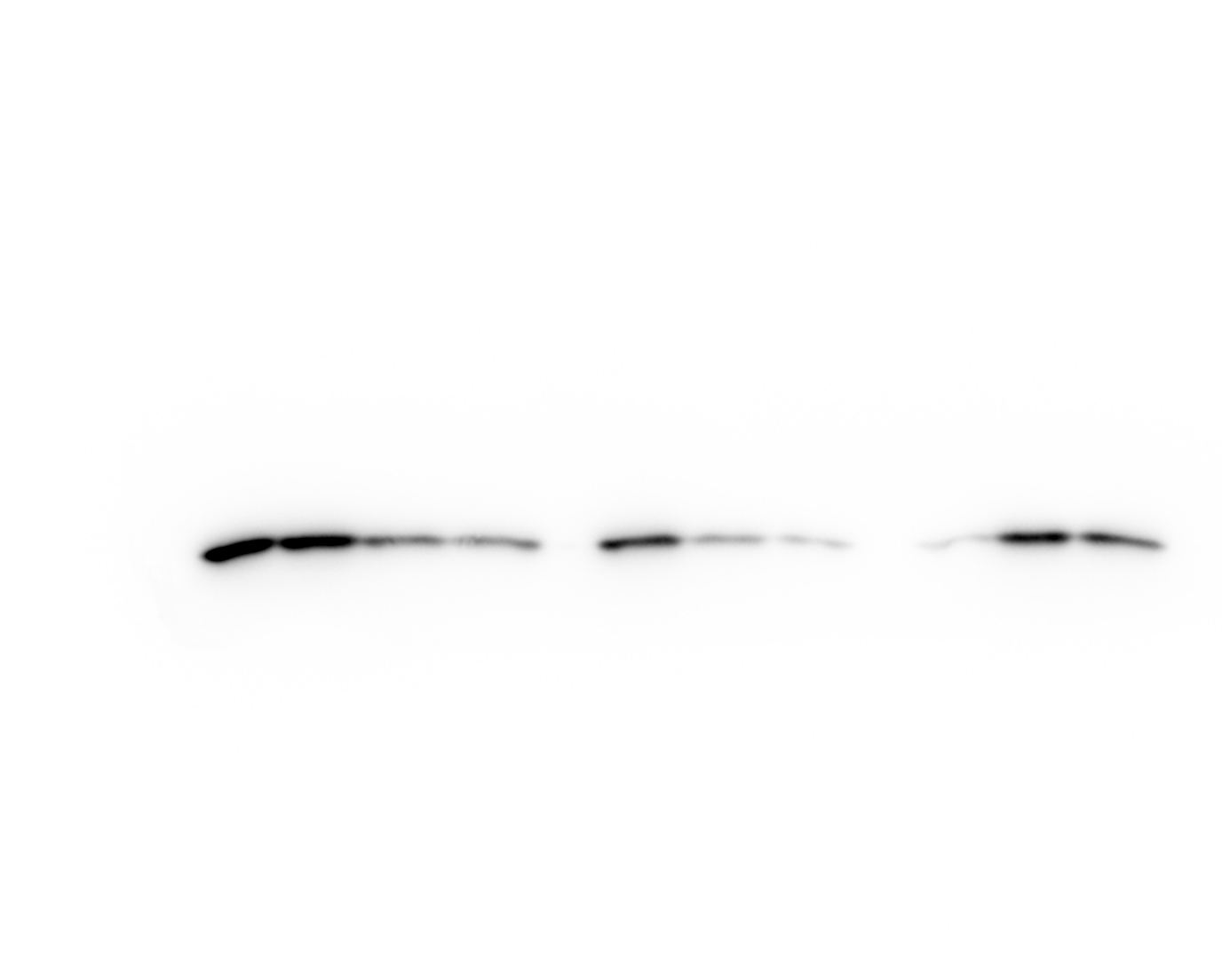

Supplement: Figure 6—figure supplement 1—source data 2. [file elife-94898-fig6-figsupp1-data2.zip › Figure 6-figure supplement 1D-GFP.Tif]

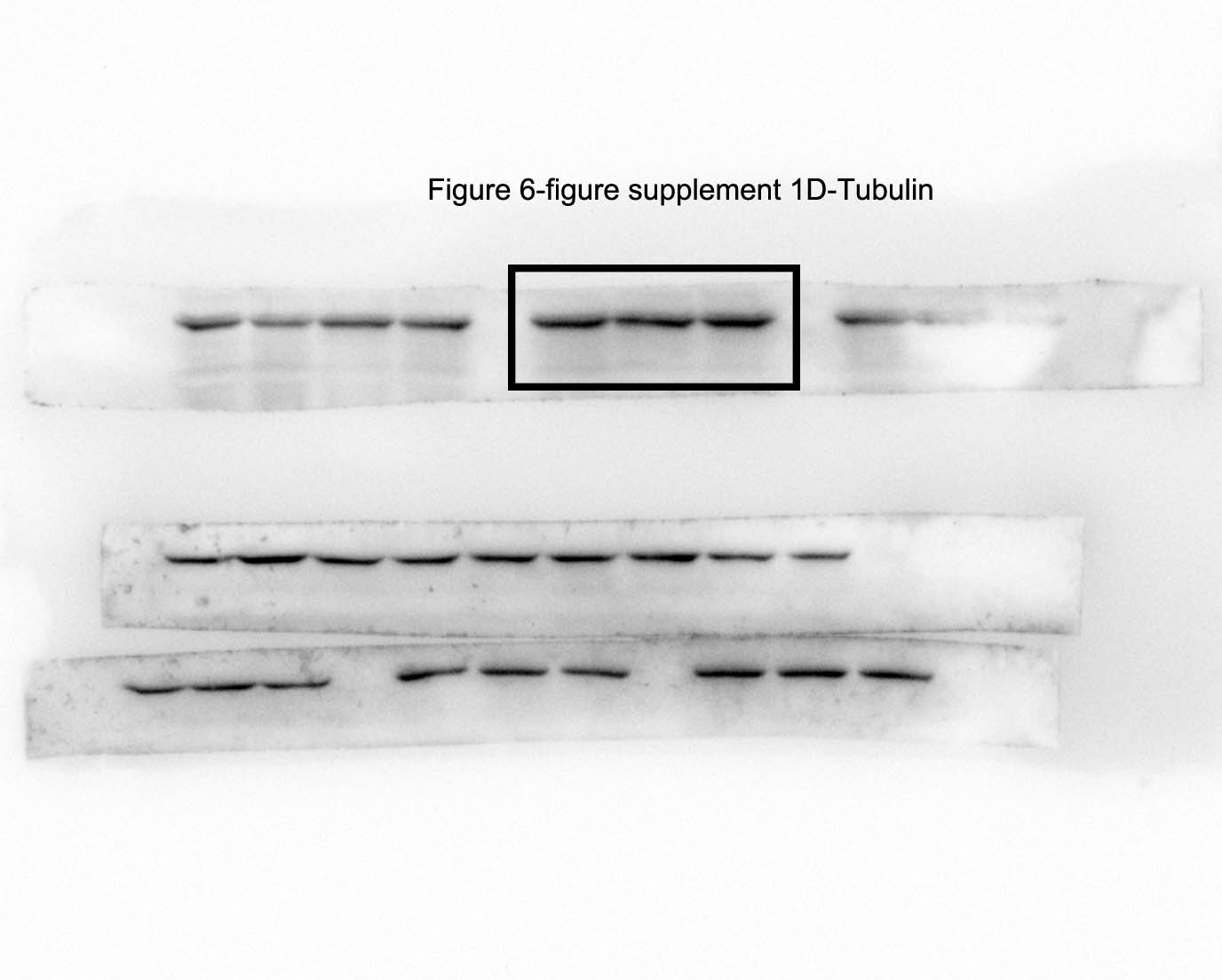

Supplement: Figure 6—figure supplement 1—source data 3. [file elife-94898-fig6-figsupp1-data3.zip › Figure 6-figure supplement 1D-Tubulin.jpg]

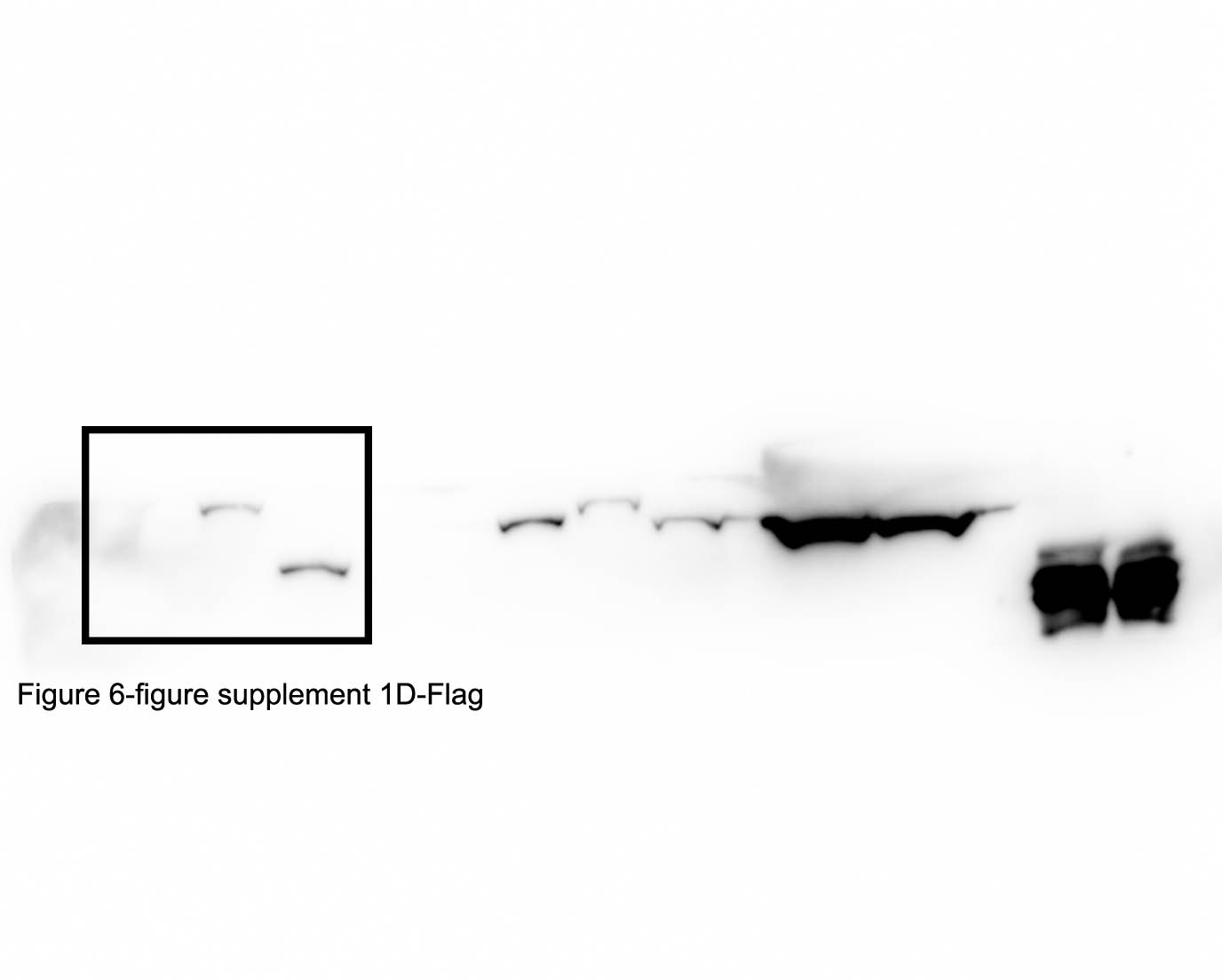

Supplement: Figure 6—figure supplement 1—source data 3. [file elife-94898-fig6-figsupp1-data3.zip › Figure 6-figure supplement 1D-Flag.jpg]

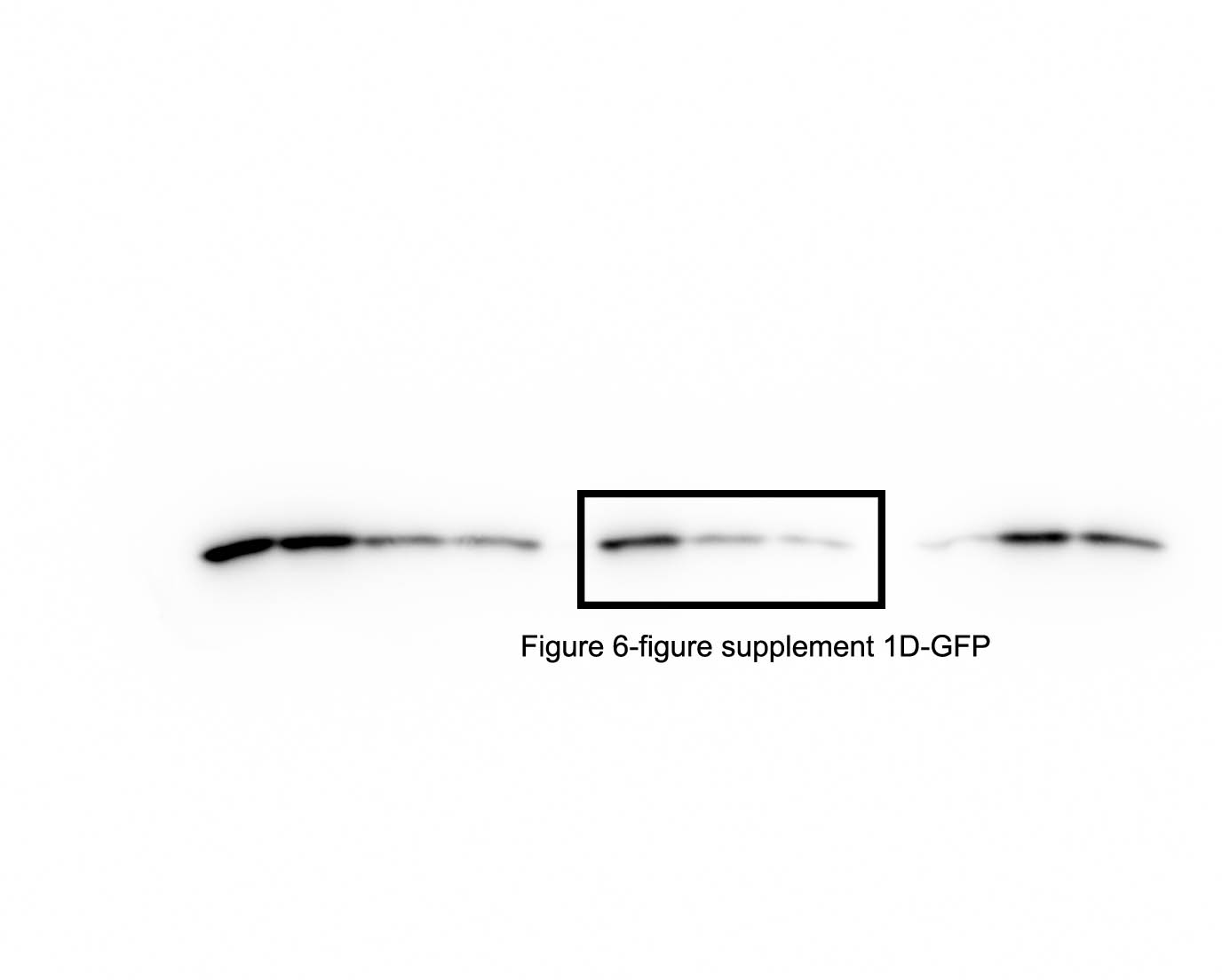

Supplement: Figure 6—figure supplement 1—source data 3. [file elife-94898-fig6-figsupp1-data3.zip › Figure 6-figure supplement 1D-GFP.jpg]

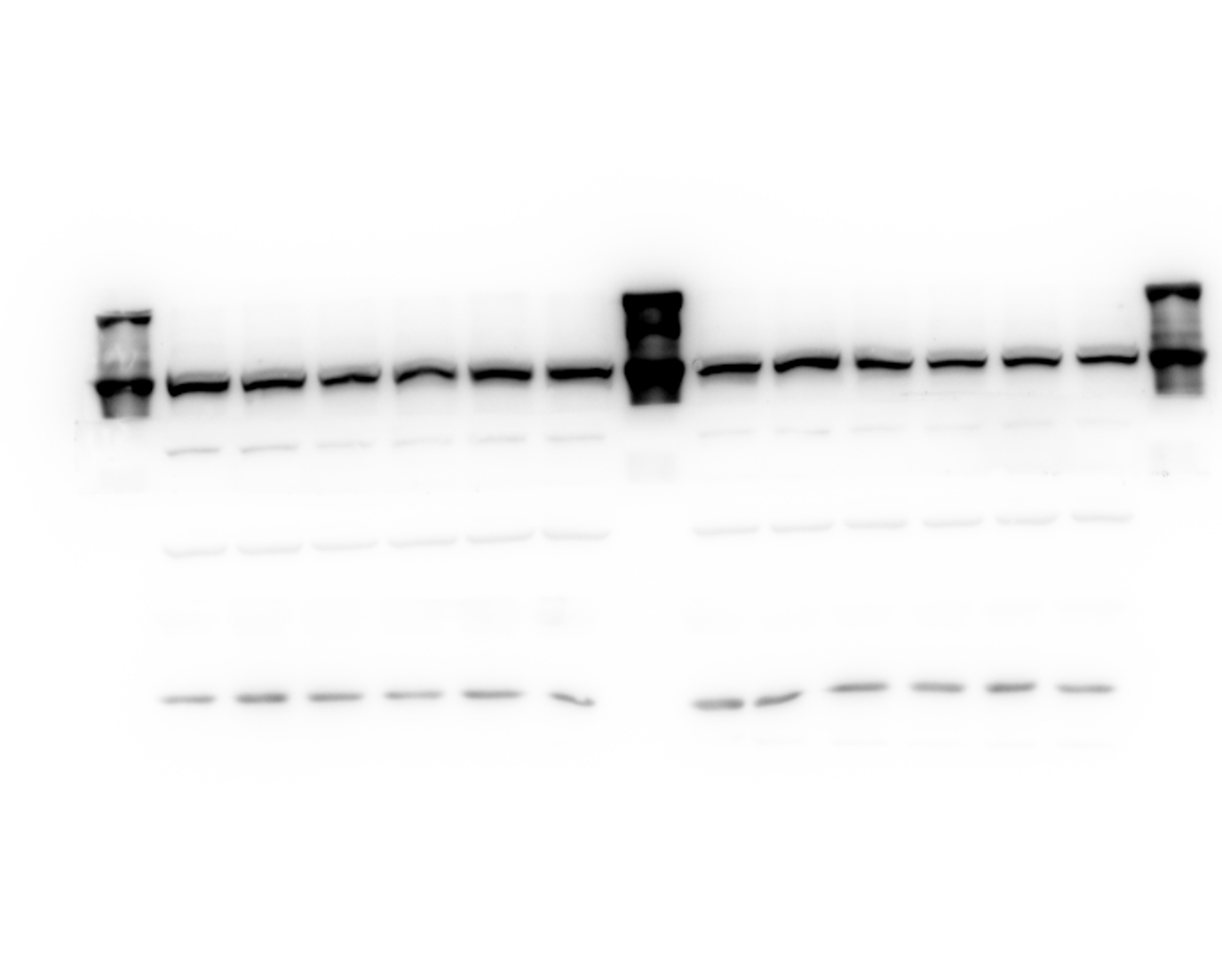

Supplement: Figure 7—source data 2. [file elife-94898-fig7-data2.zip › Fig7D-MDA5.Tif]

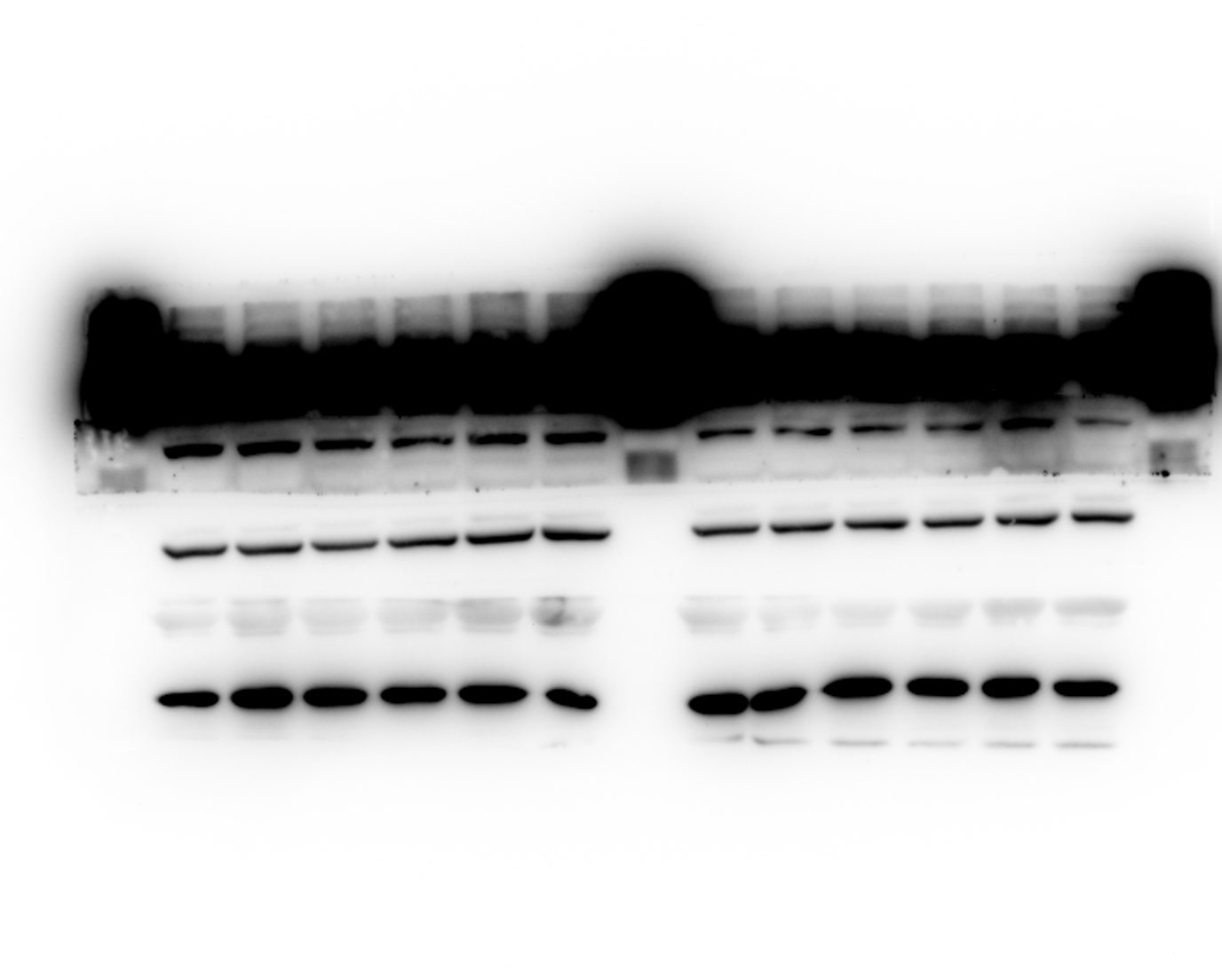

Supplement: Figure 7—source data 2. [file elife-94898-fig7-data2.zip › Fig7D-Tubulin.Tif]

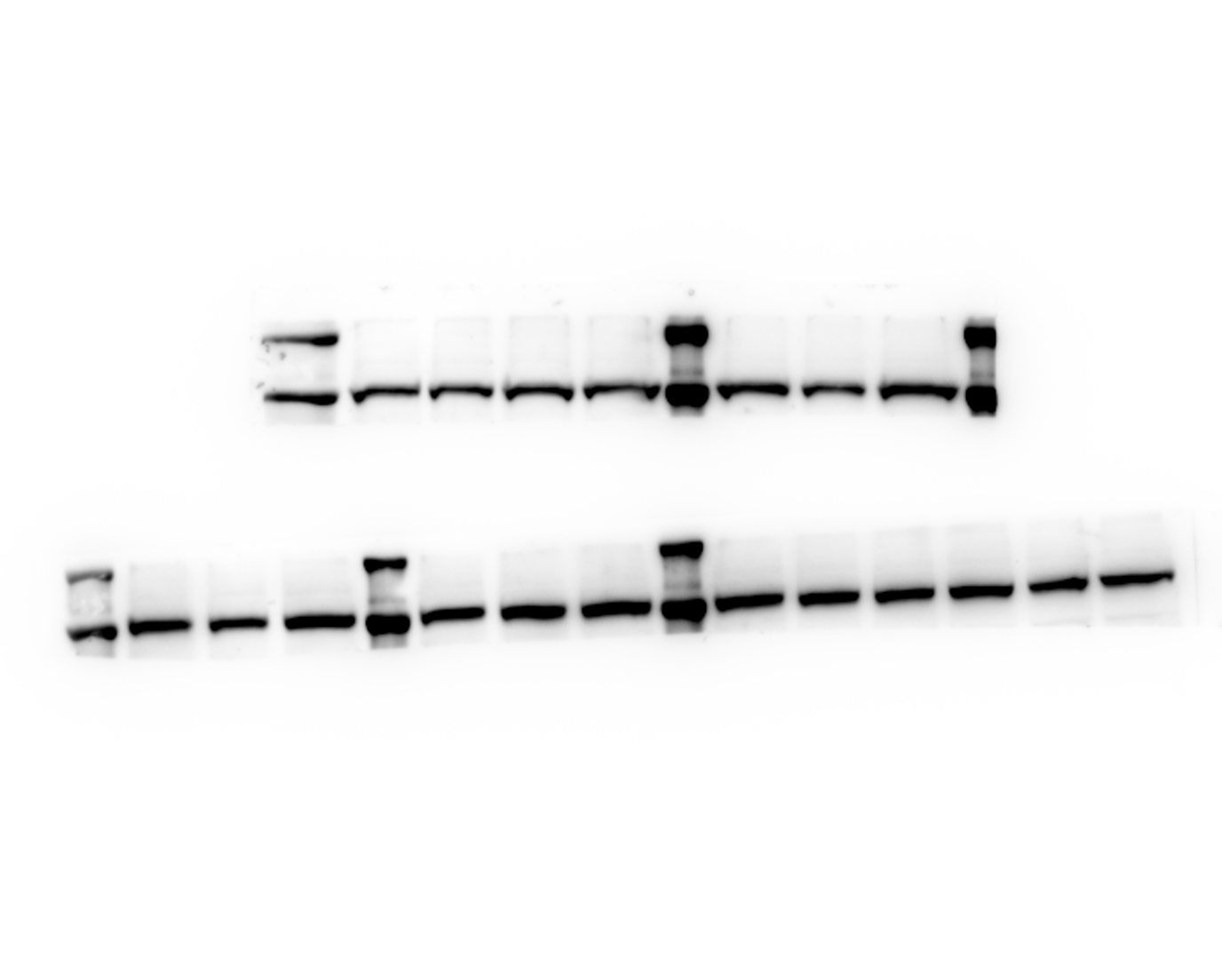

Supplement: Figure 7—source data 2. [file elife-94898-fig7-data2.zip › Fig7E-MDA5.Tif]

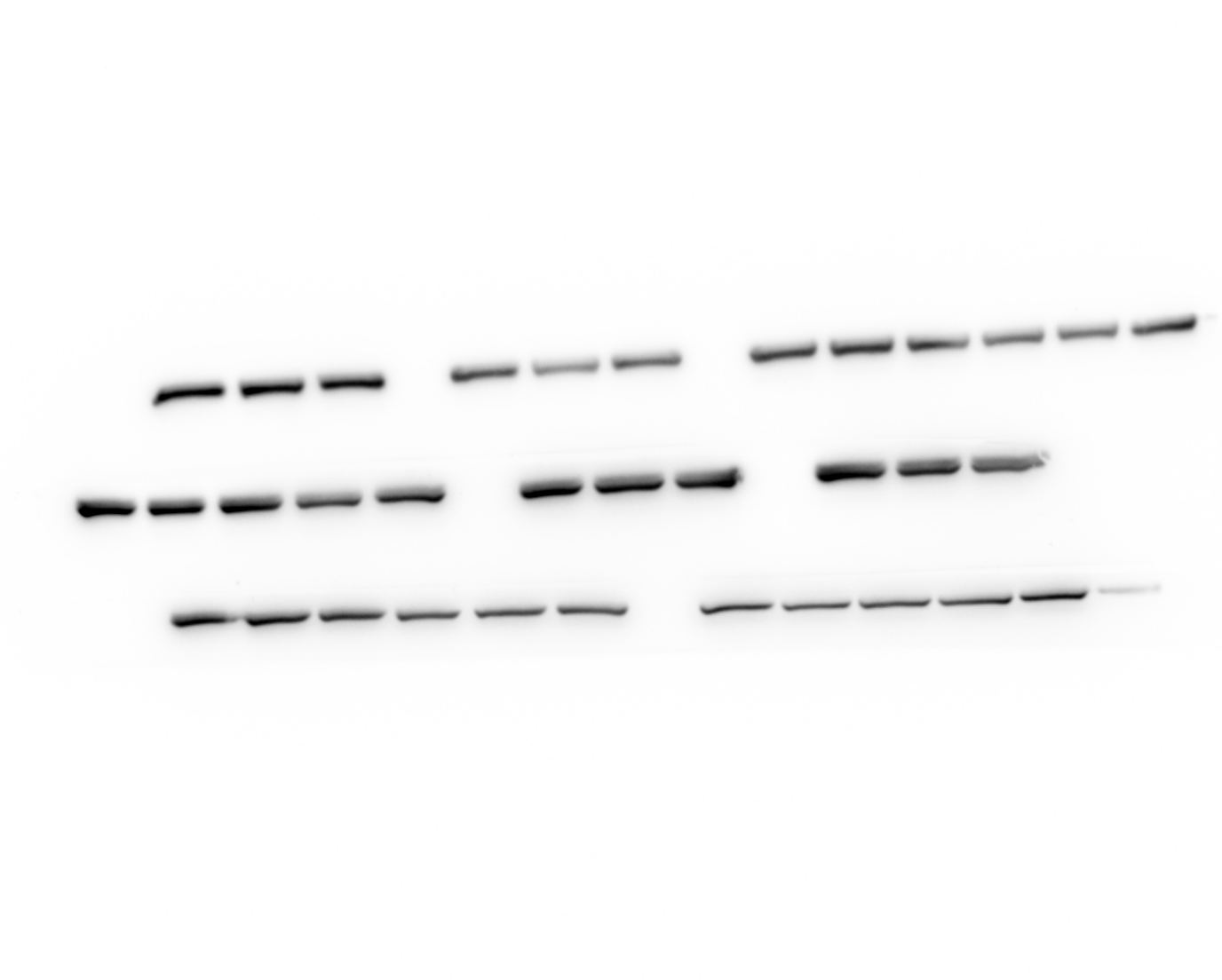

Supplement: Figure 7—source data 2. [file elife-94898-fig7-data2.zip › Fig7E-Tubulin.Tif]

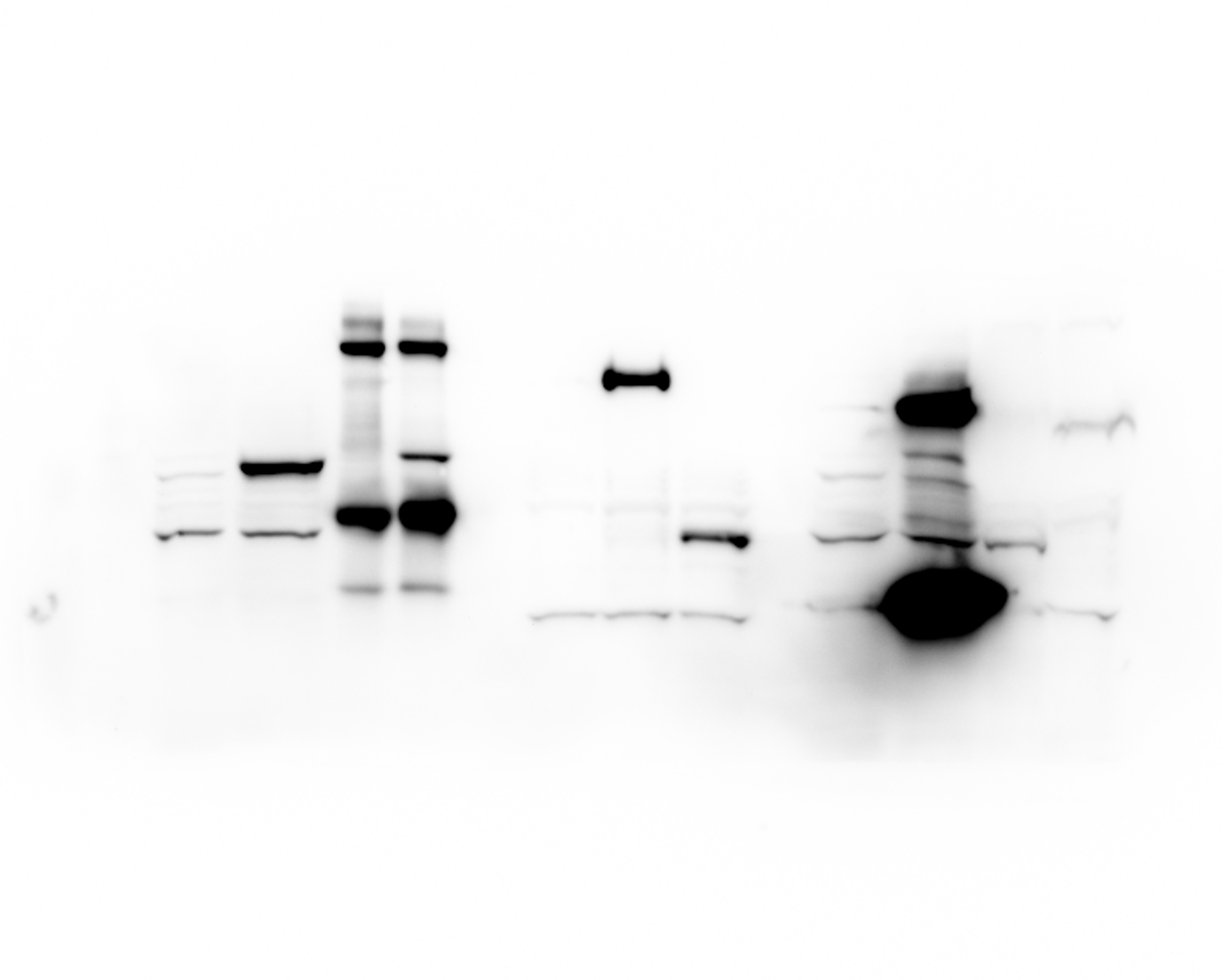

Supplement: Figure 7—source data 2. [file elife-94898-fig7-data2.zip › Fig7G-Flag.Tif]

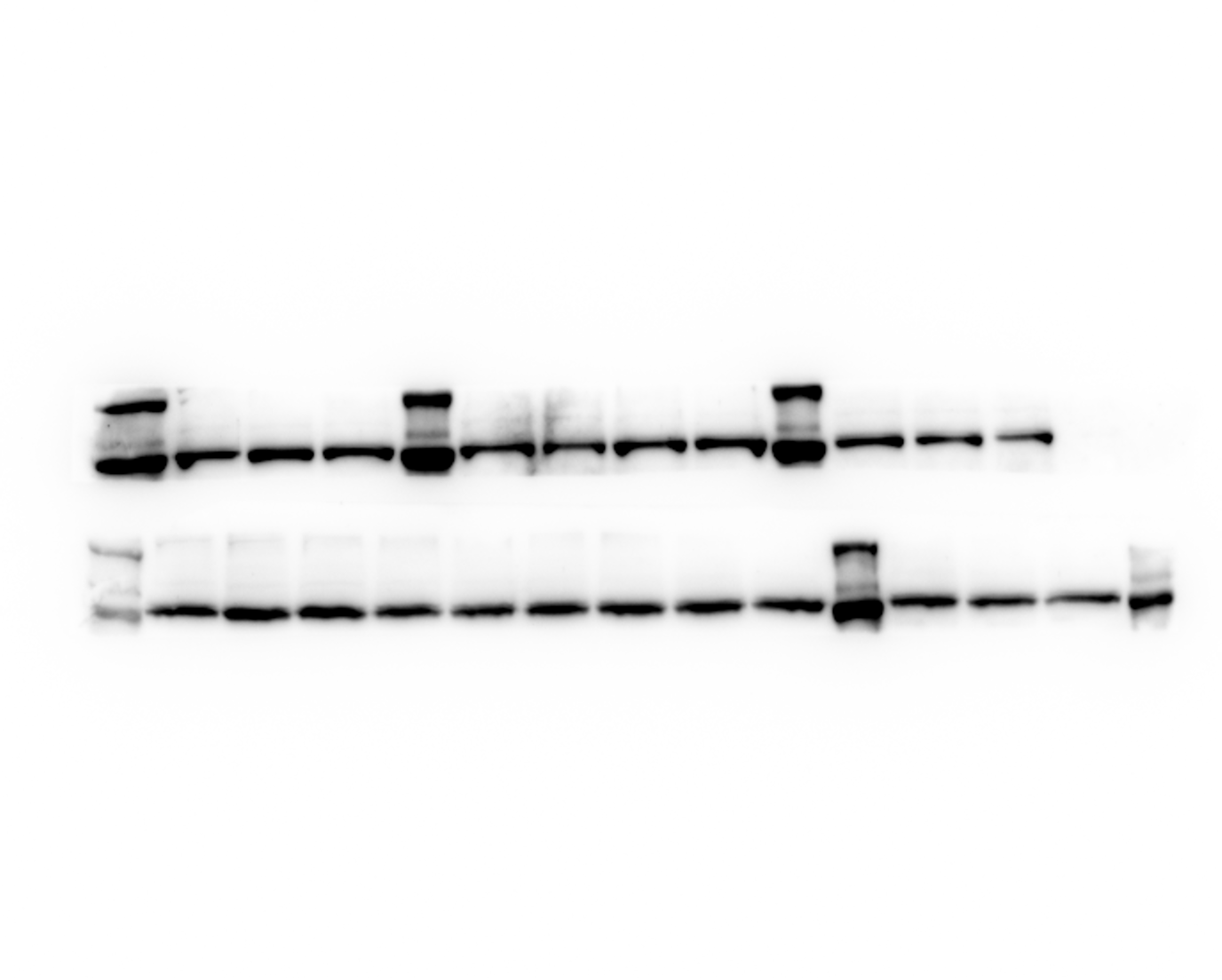

Supplement: Figure 7—source data 2. [file elife-94898-fig7-data2.zip › Fig7G-MDA5.Tif]

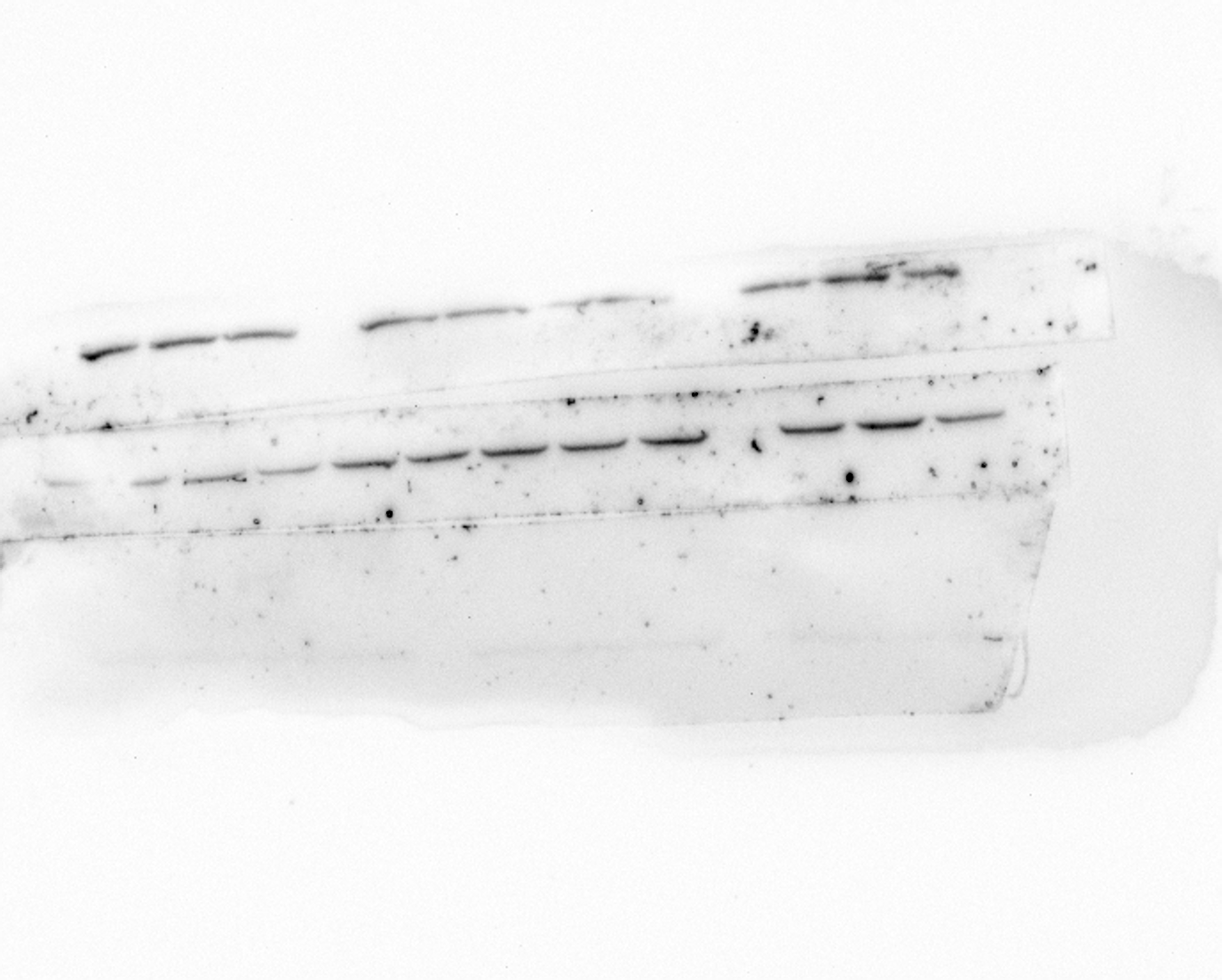

Supplement: Figure 7—source data 2. [file elife-94898-fig7-data2.zip › Fig7G-Tubulin.Tif]

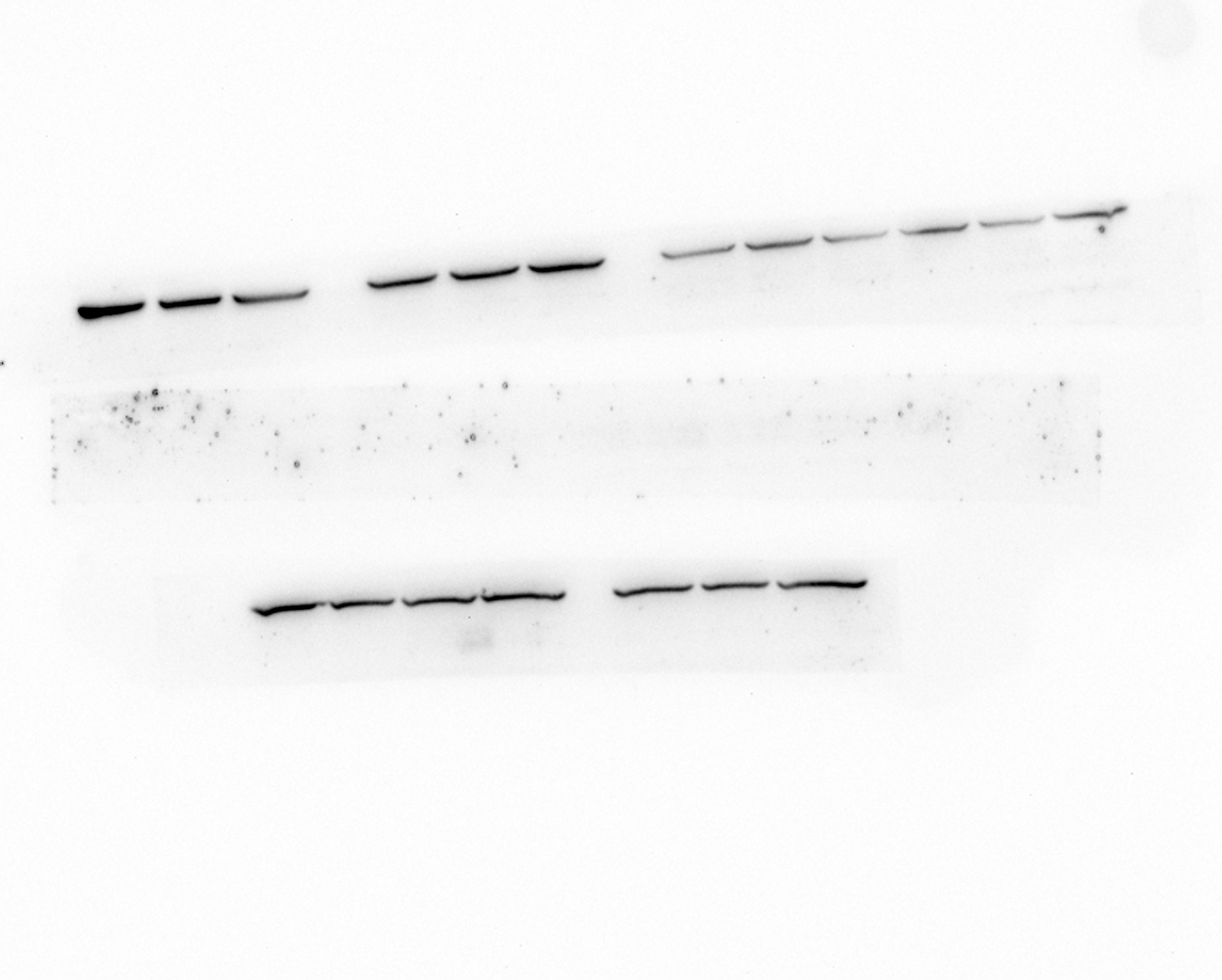

Supplement: Figure 7—source data 2. [file elife-94898-fig7-data2.zip › Fig7H-Tubulin.Tif]

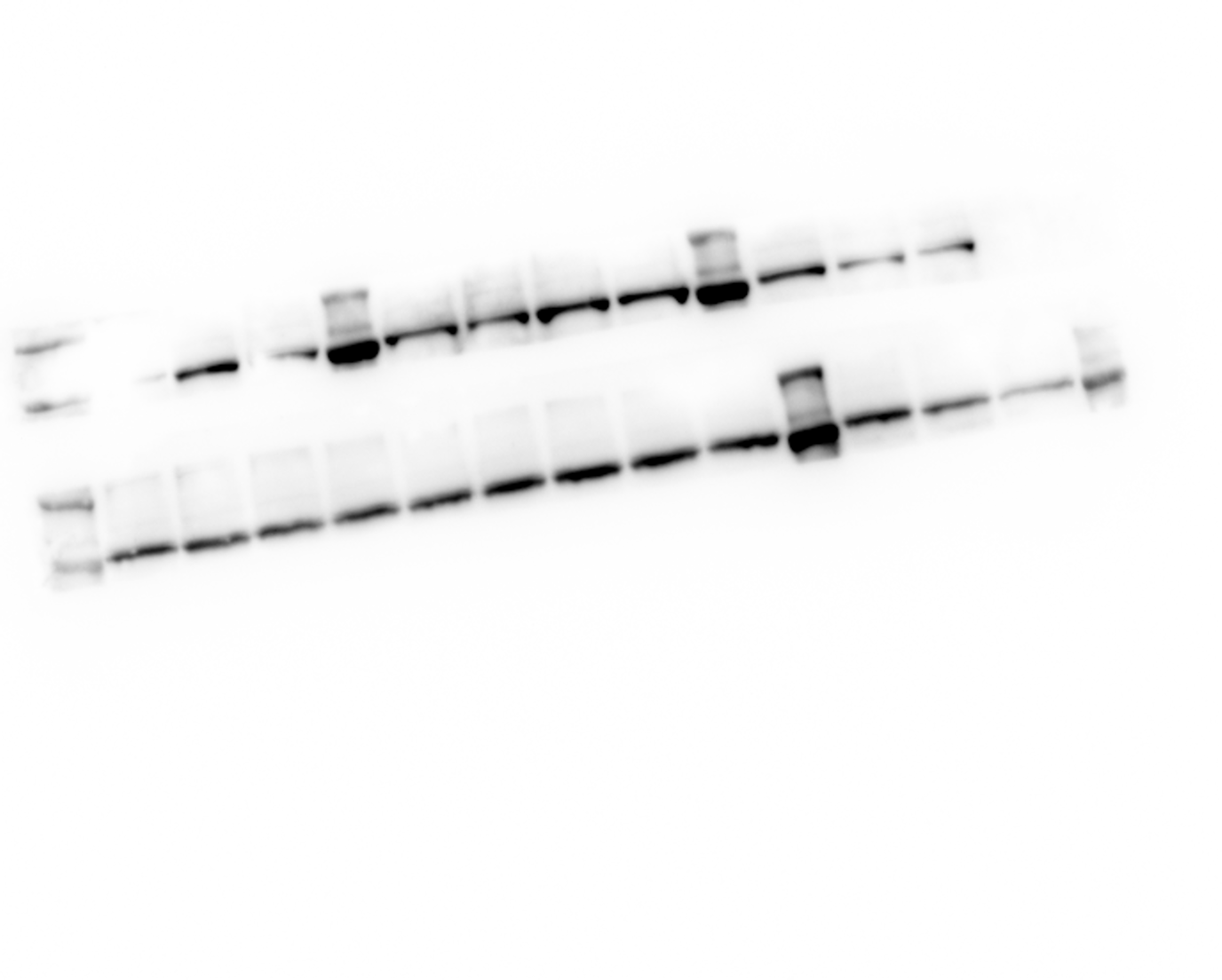

Supplement: Figure 7—source data 2. [file elife-94898-fig7-data2.zip › Fig7C-MDA5.Tif]

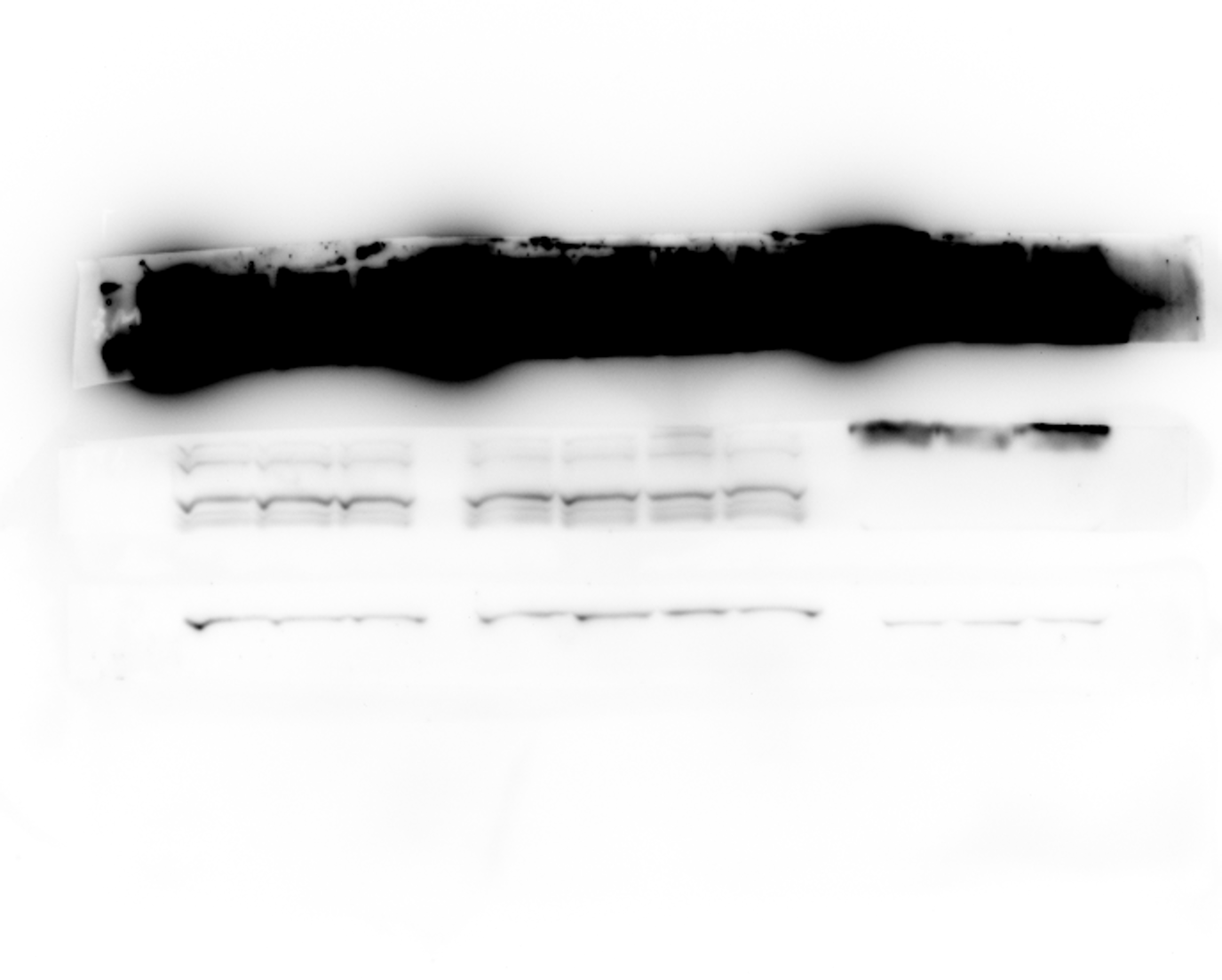

Supplement: Figure 7—source data 2. [file elife-94898-fig7-data2.zip › Fig7C-Tubulin.Tif]
