# Supplementary figures and images for: An arms race between 5’ppp-RNA virus and its alternative recognition receptor MDA5 in RIG-I-lost teleost fish (part 2 of 2)
Source: eLife. 2024 Sep 30;13:RP94898. doi: 10.7554/eLife.94898 (PMC11441976; doi:10.7554/eLife.94898)

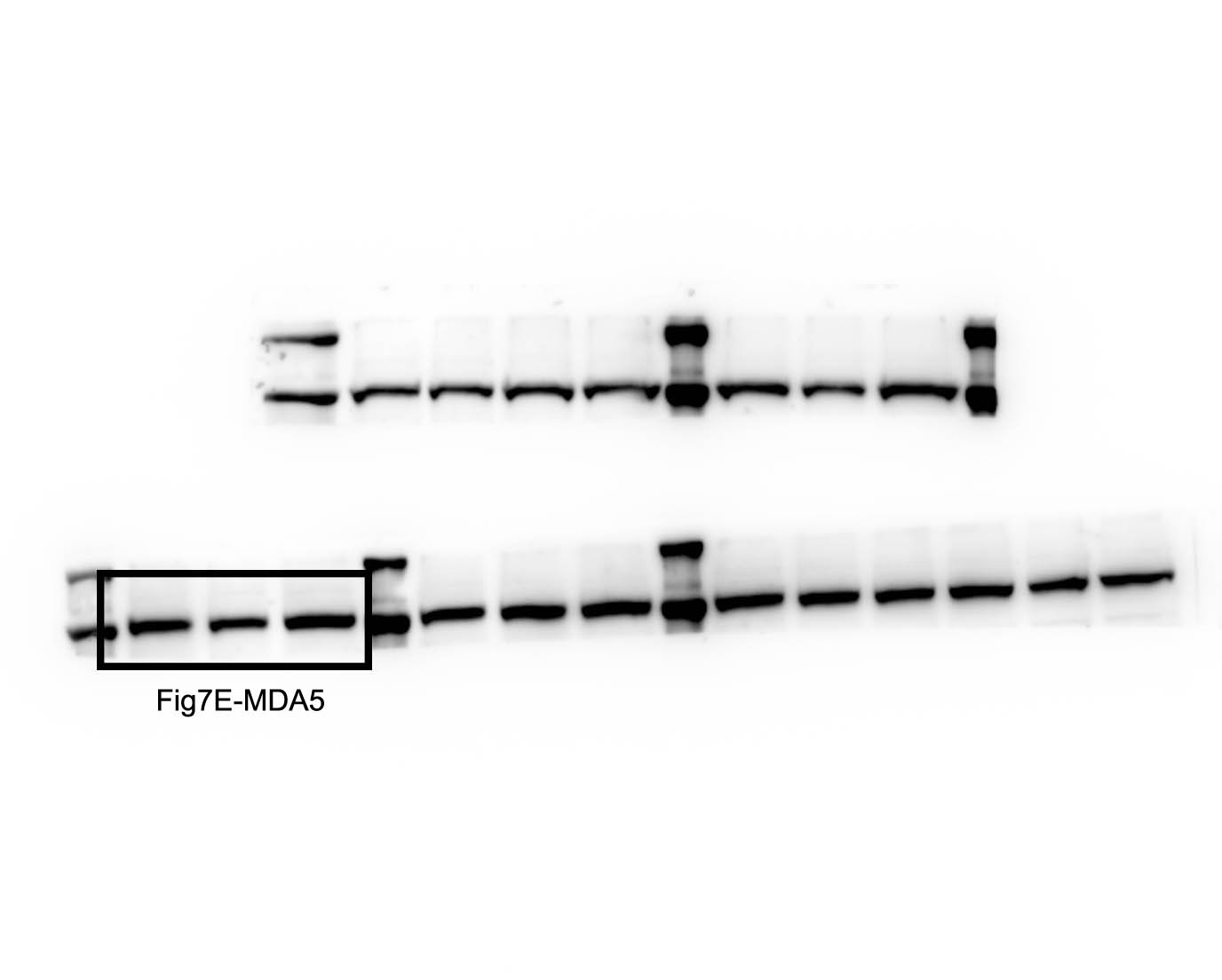

Supplement: Figure 7—source data 3. [file elife-94898-fig7-data3.zip › Fig7E-MDA5.jpg]

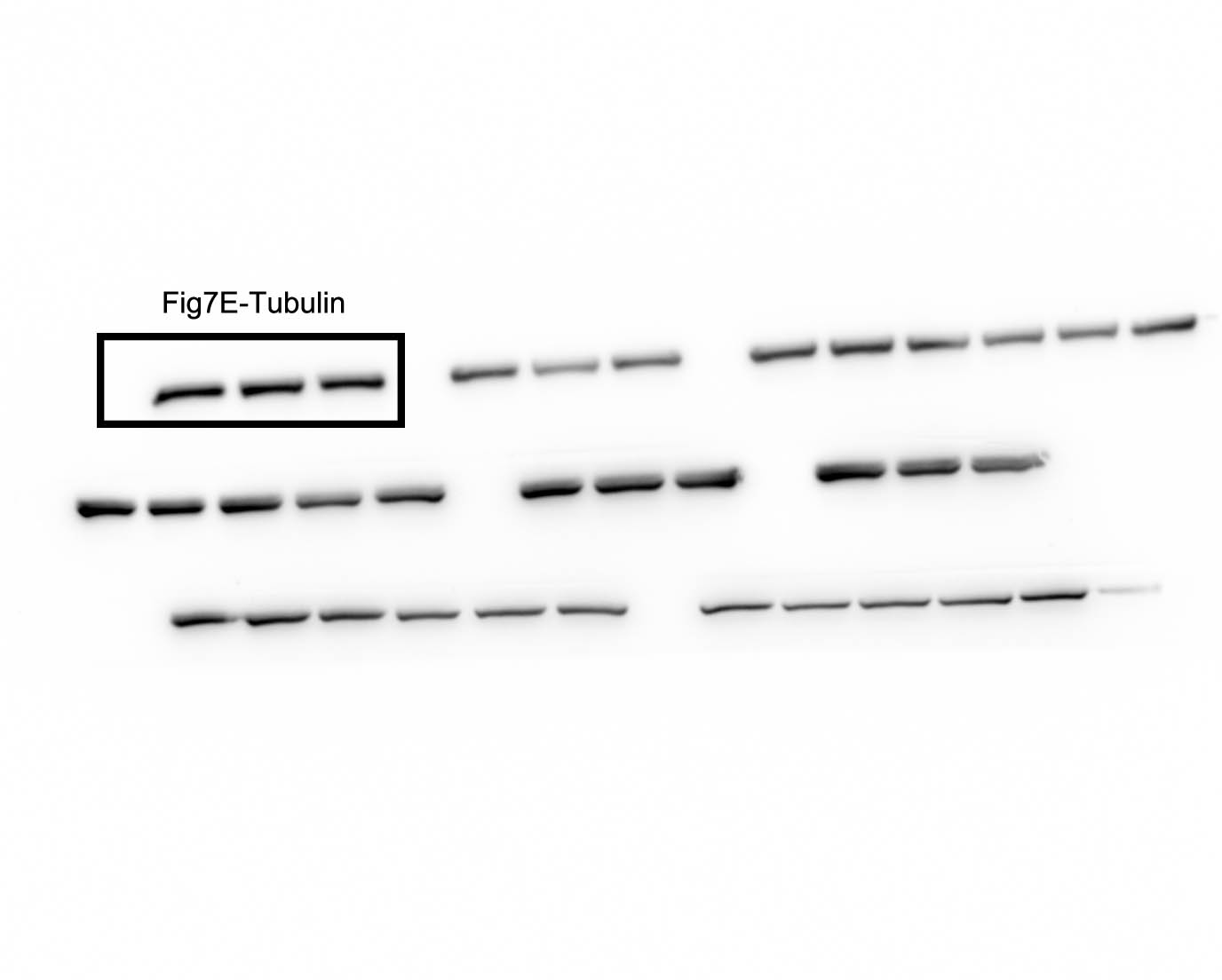

Supplement: Figure 7—source data 3. [file elife-94898-fig7-data3.zip › Fig7E-Tubulin.jpg]

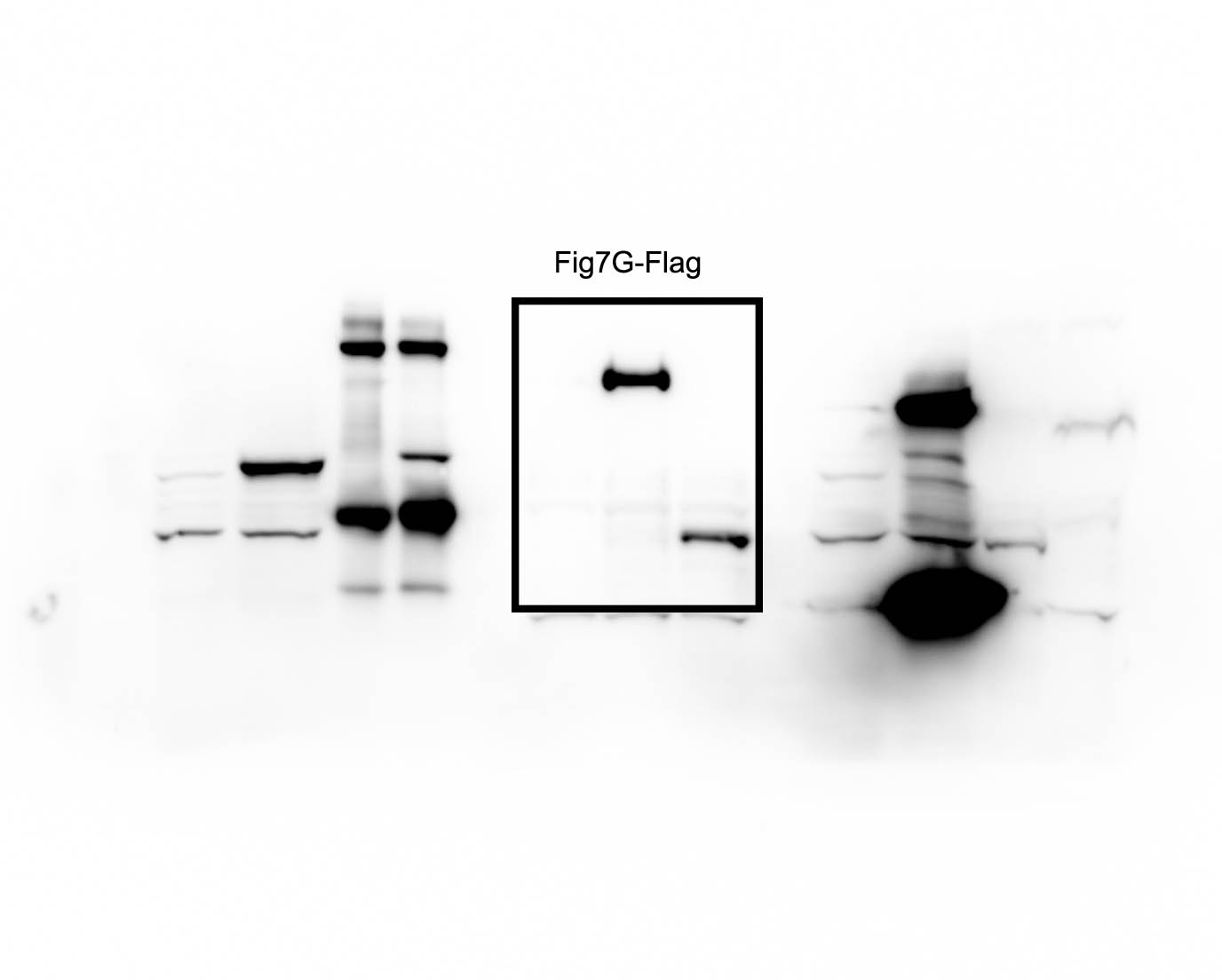

Supplement: Figure 7—source data 3. [file elife-94898-fig7-data3.zip › Fig7G-Flag.jpg]

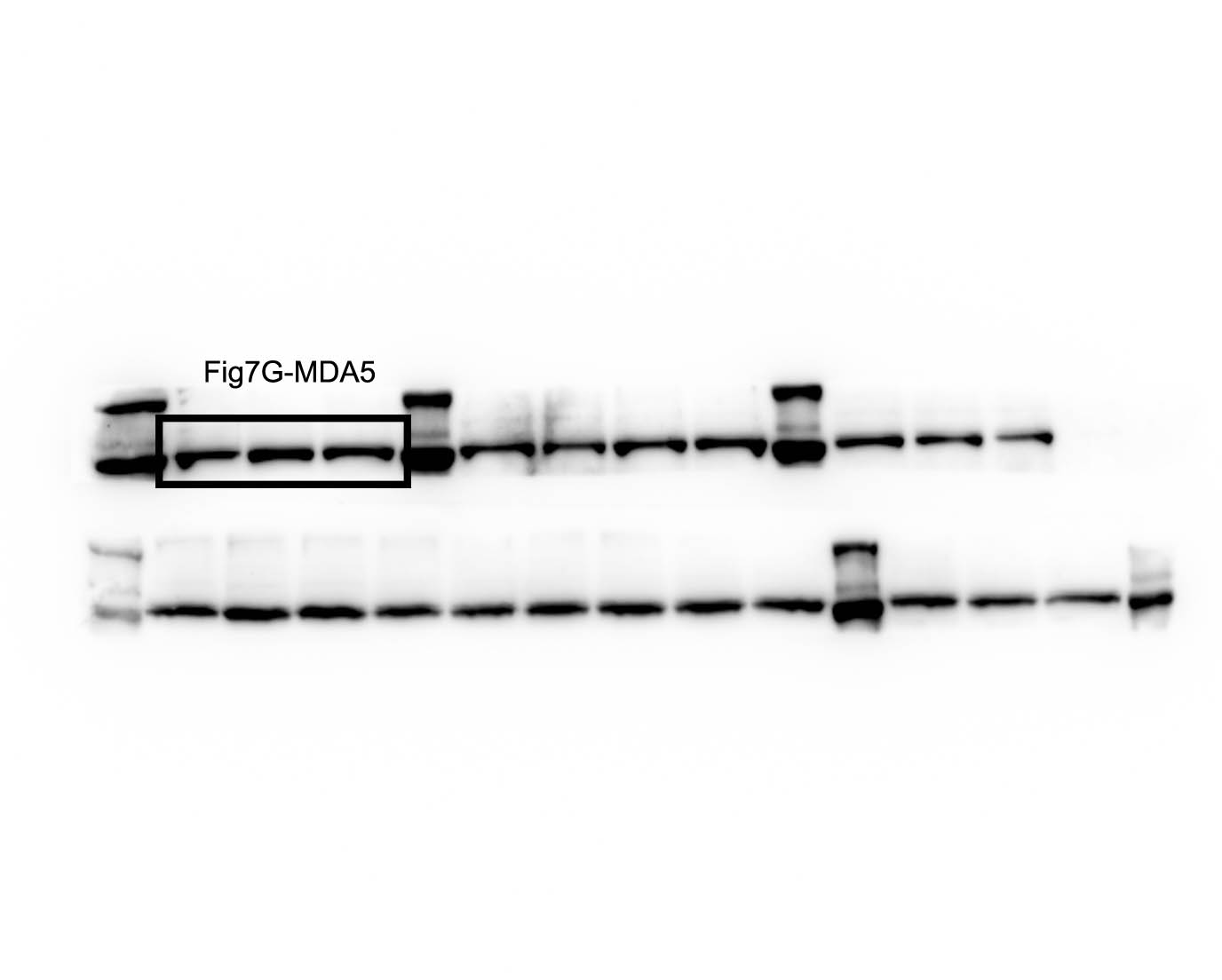

Supplement: Figure 7—source data 3. [file elife-94898-fig7-data3.zip › Fig7G-MDA5.jpg]

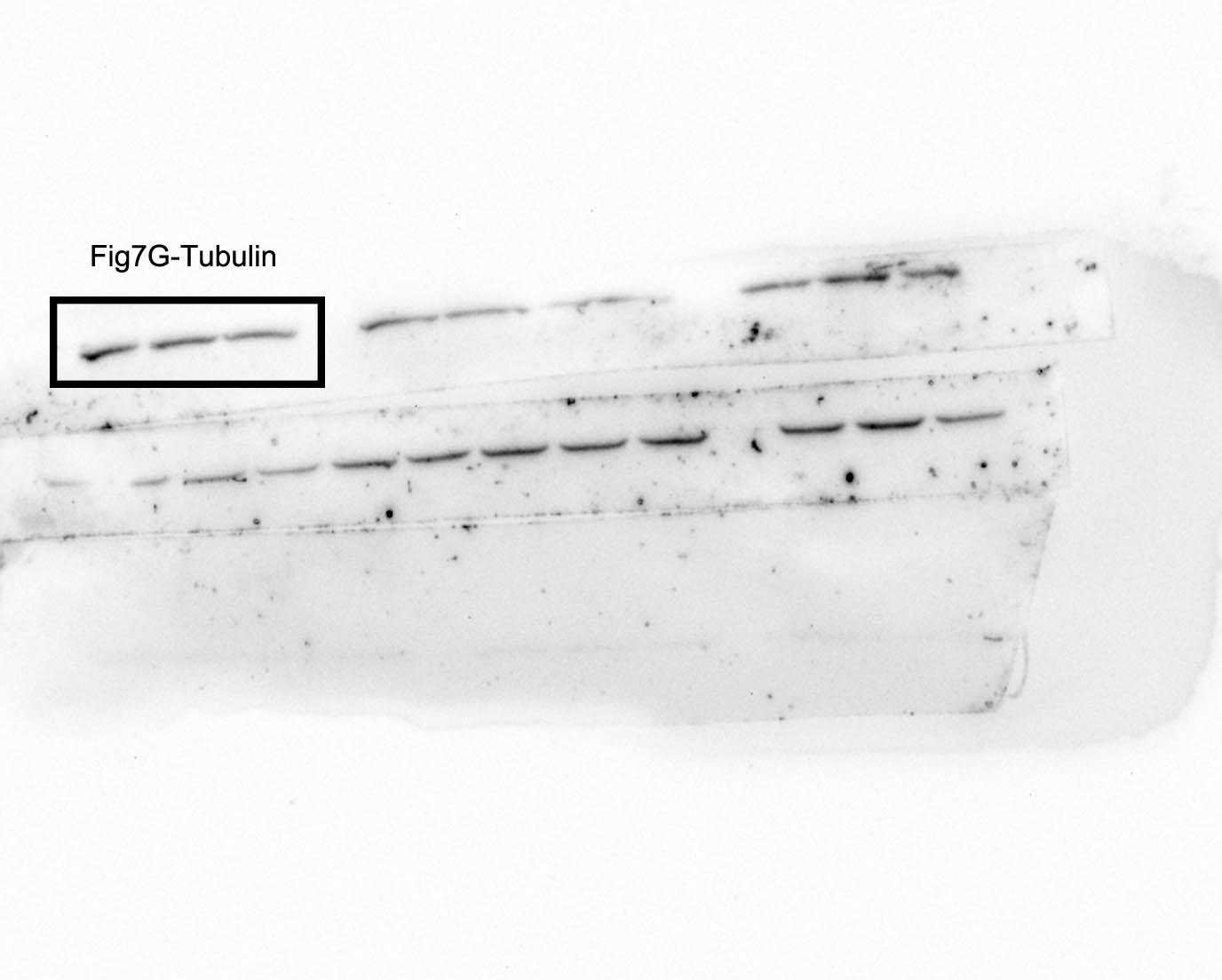

Supplement: Figure 7—source data 3. [file elife-94898-fig7-data3.zip › Fig7G-Tubulin.jpg]

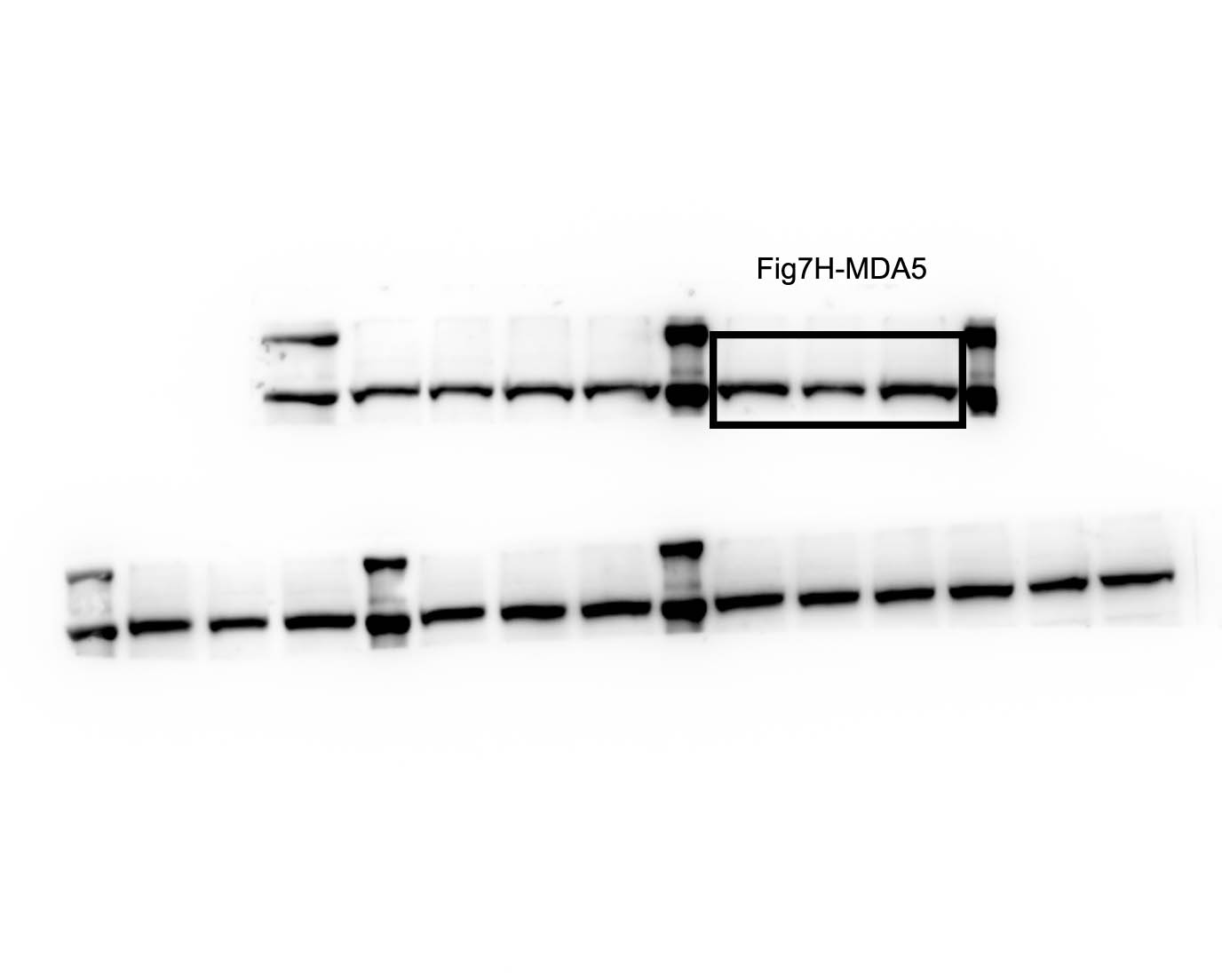

Supplement: Figure 7—source data 3. [file elife-94898-fig7-data3.zip › Fig7H-MDA5.jpg]

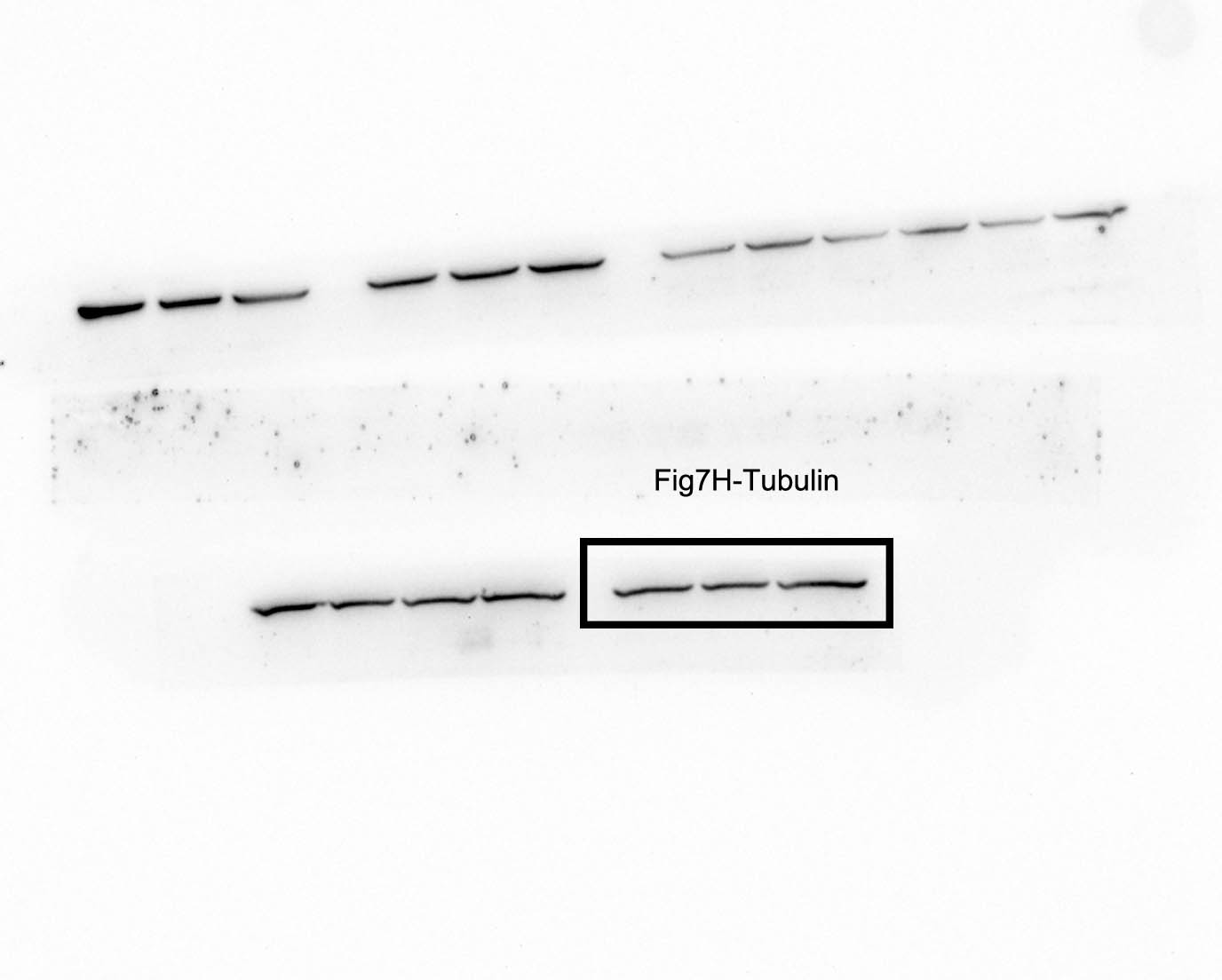

Supplement: Figure 7—source data 3. [file elife-94898-fig7-data3.zip › Fig7H-Tubulin.jpg]

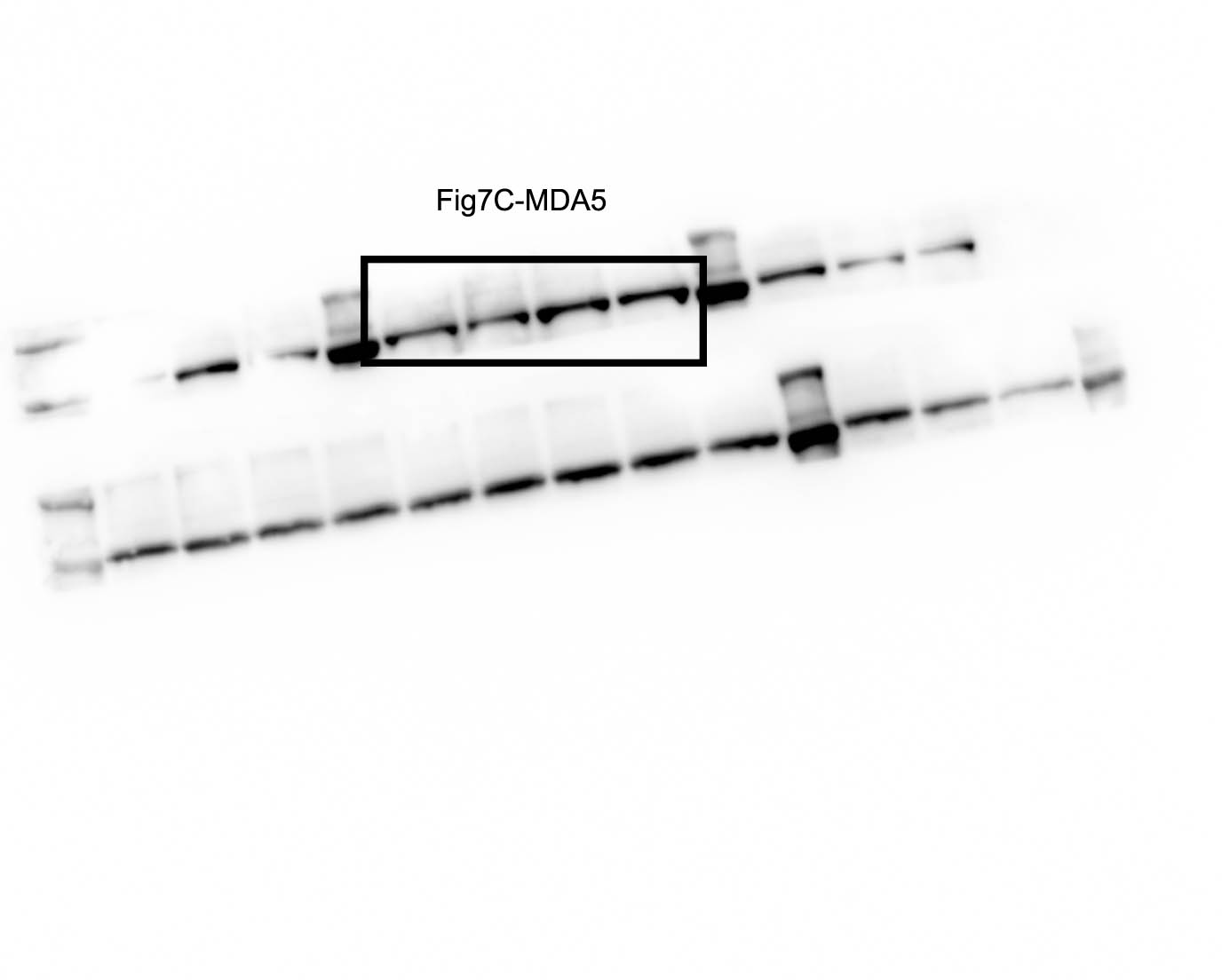

Supplement: Figure 7—source data 3. [file elife-94898-fig7-data3.zip › Fig7C-MDA5.jpg]

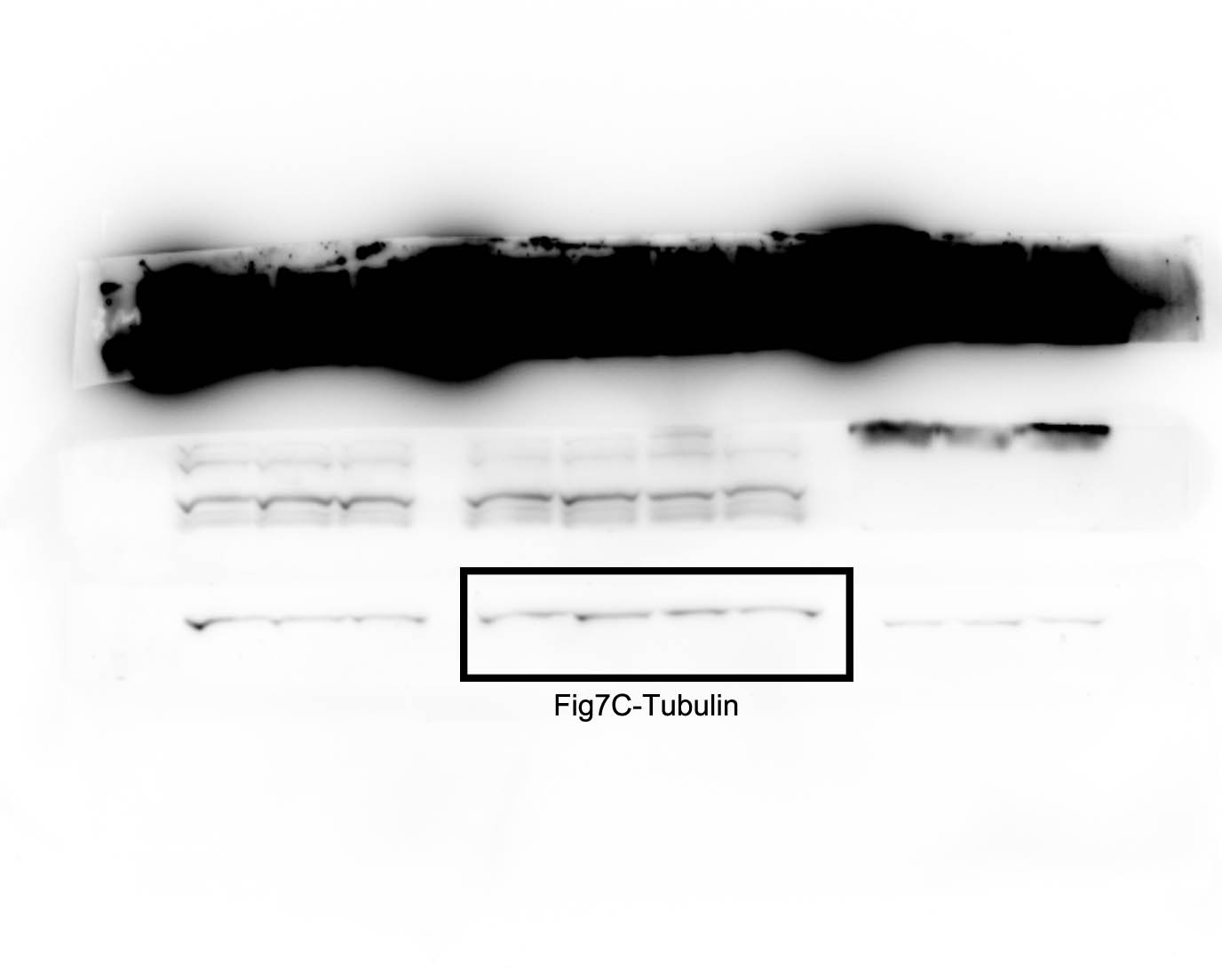

Supplement: Figure 7—source data 3. [file elife-94898-fig7-data3.zip › Fig7C-Tubulin.jpg]

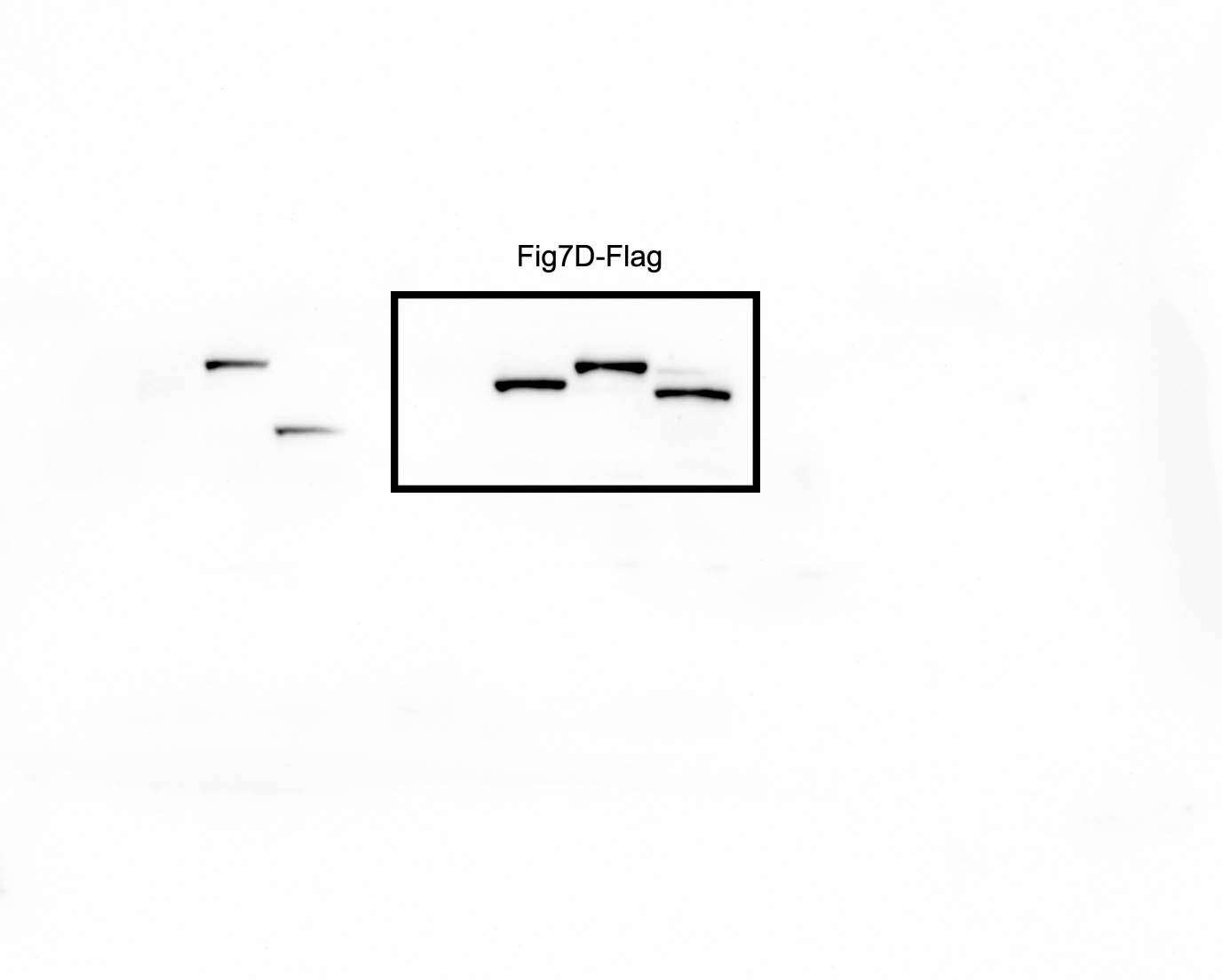

Supplement: Figure 7—source data 3. [file elife-94898-fig7-data3.zip › Fig7D-Flag.jpg]

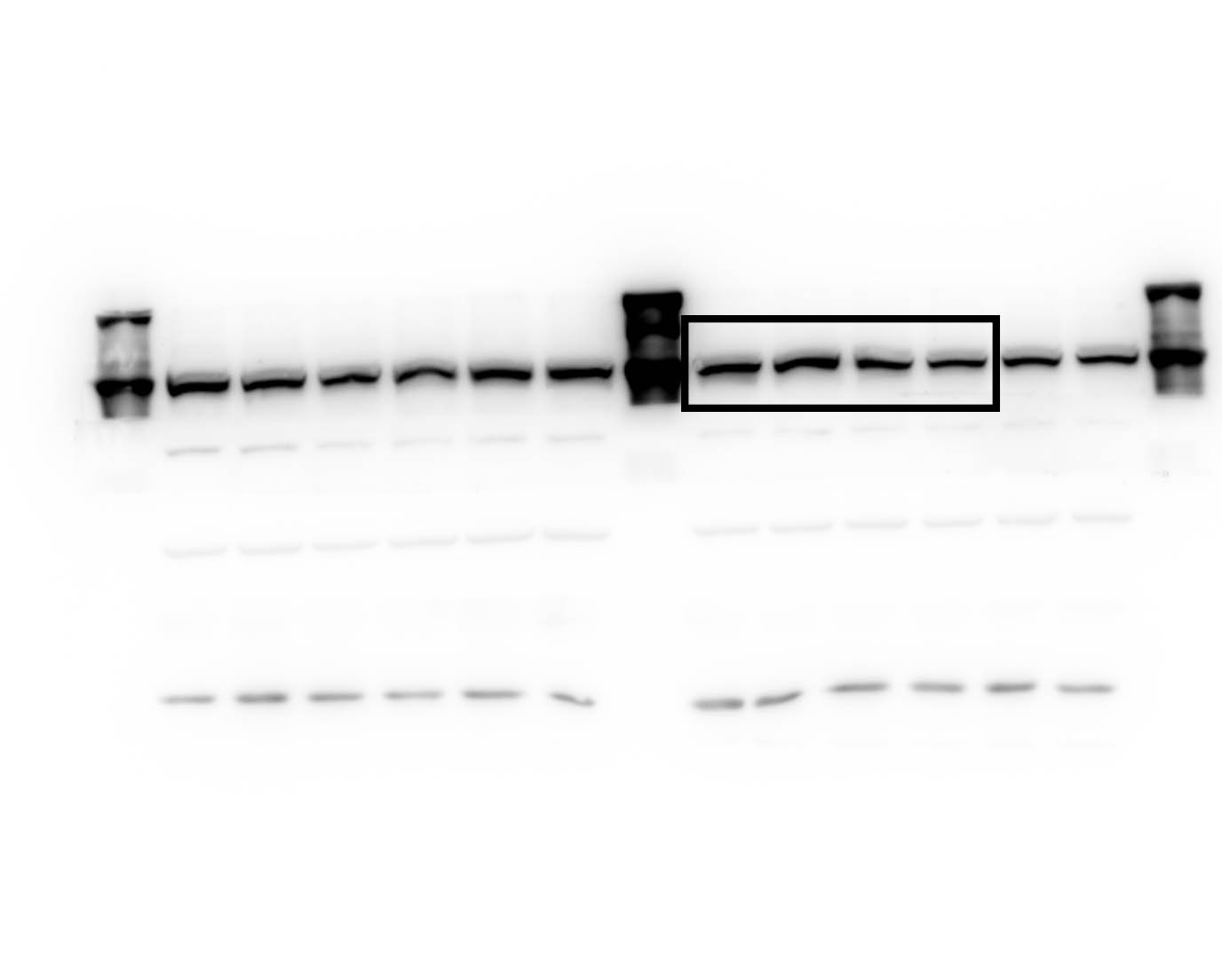

Supplement: Figure 7—source data 3. [file elife-94898-fig7-data3.zip › Fig7D-MDA5.jpg]

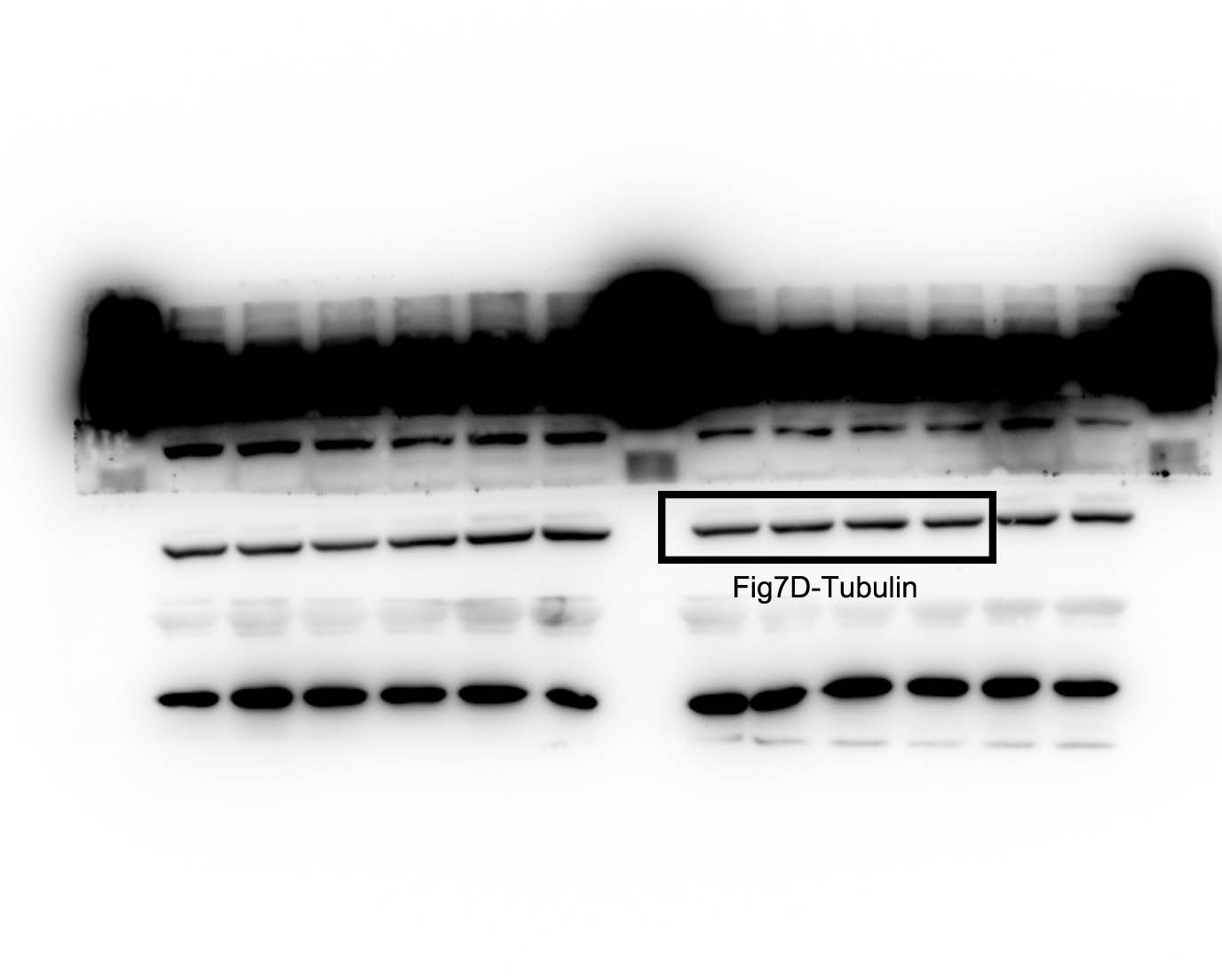

Supplement: Figure 7—source data 3. [file elife-94898-fig7-data3.zip › Fig7D-Tubulin.jpg]

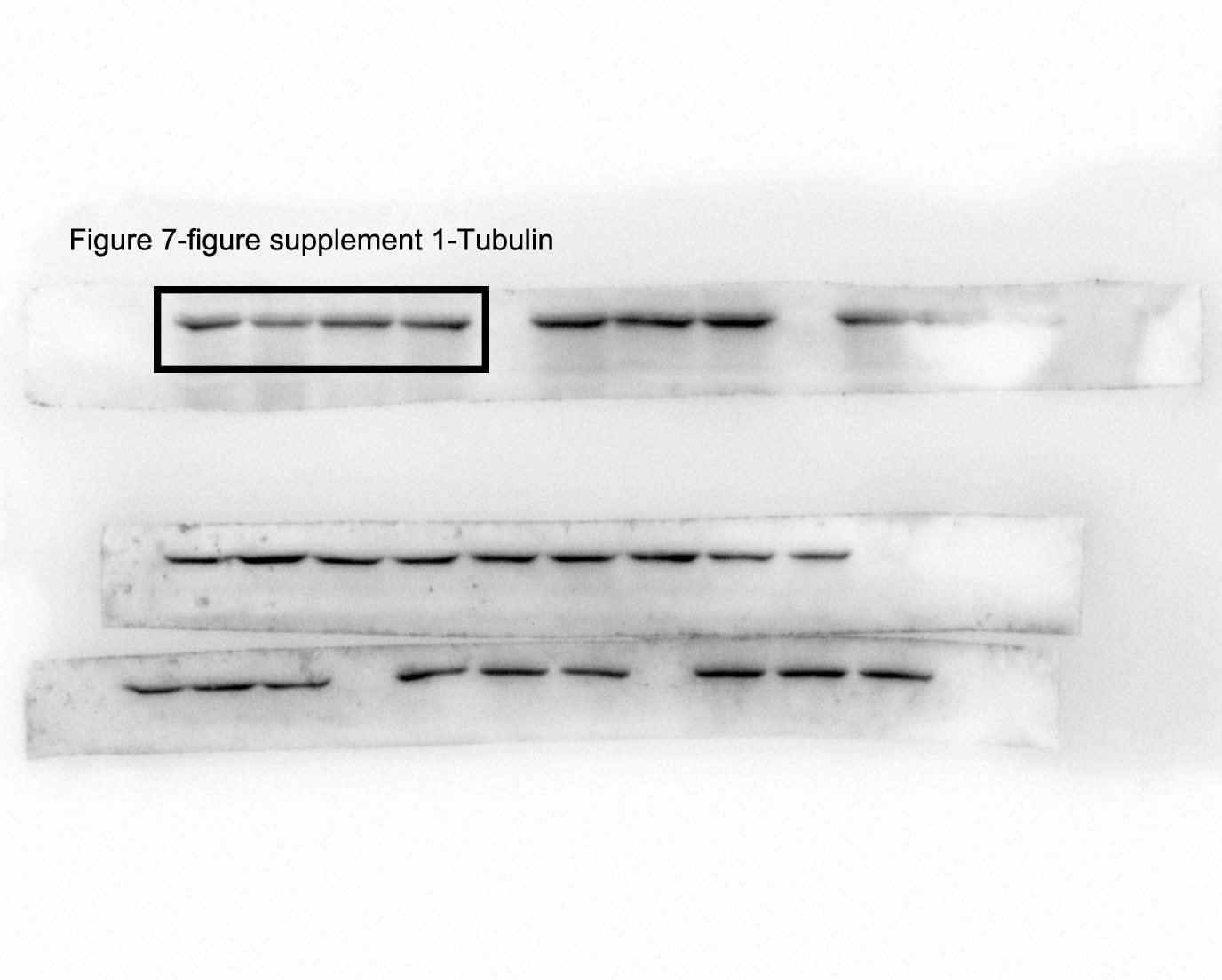

Supplement: Figure 7—figure supplement 1—source data 3. [file elife-94898-fig7-figsupp1-data3.zip › Figure 7-figure supplement 1-Tubulin.jpg]

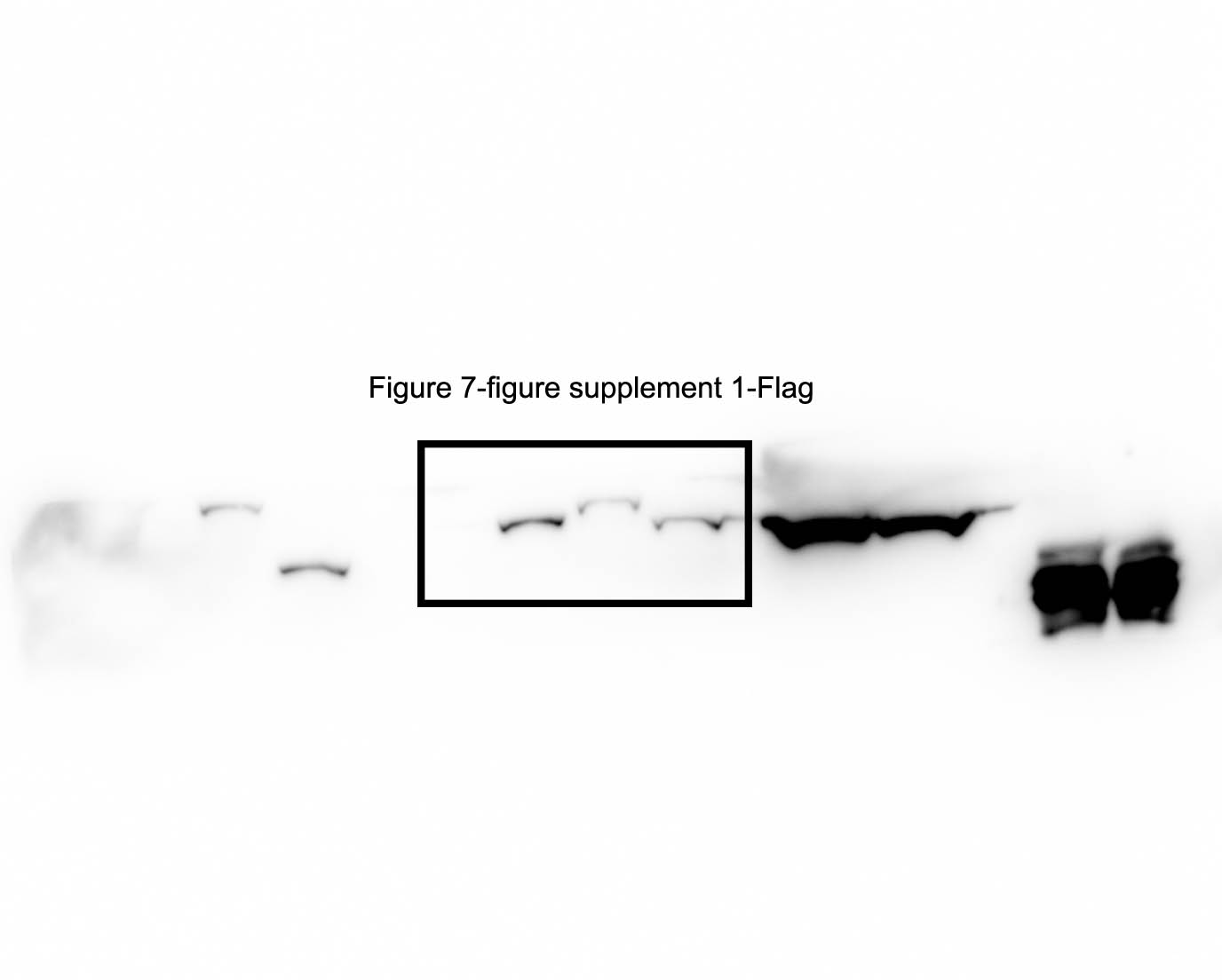

Supplement: Figure 7—figure supplement 1—source data 3. [file elife-94898-fig7-figsupp1-data3.zip › Figure 7-figure supplement 1-Flag.jpg]

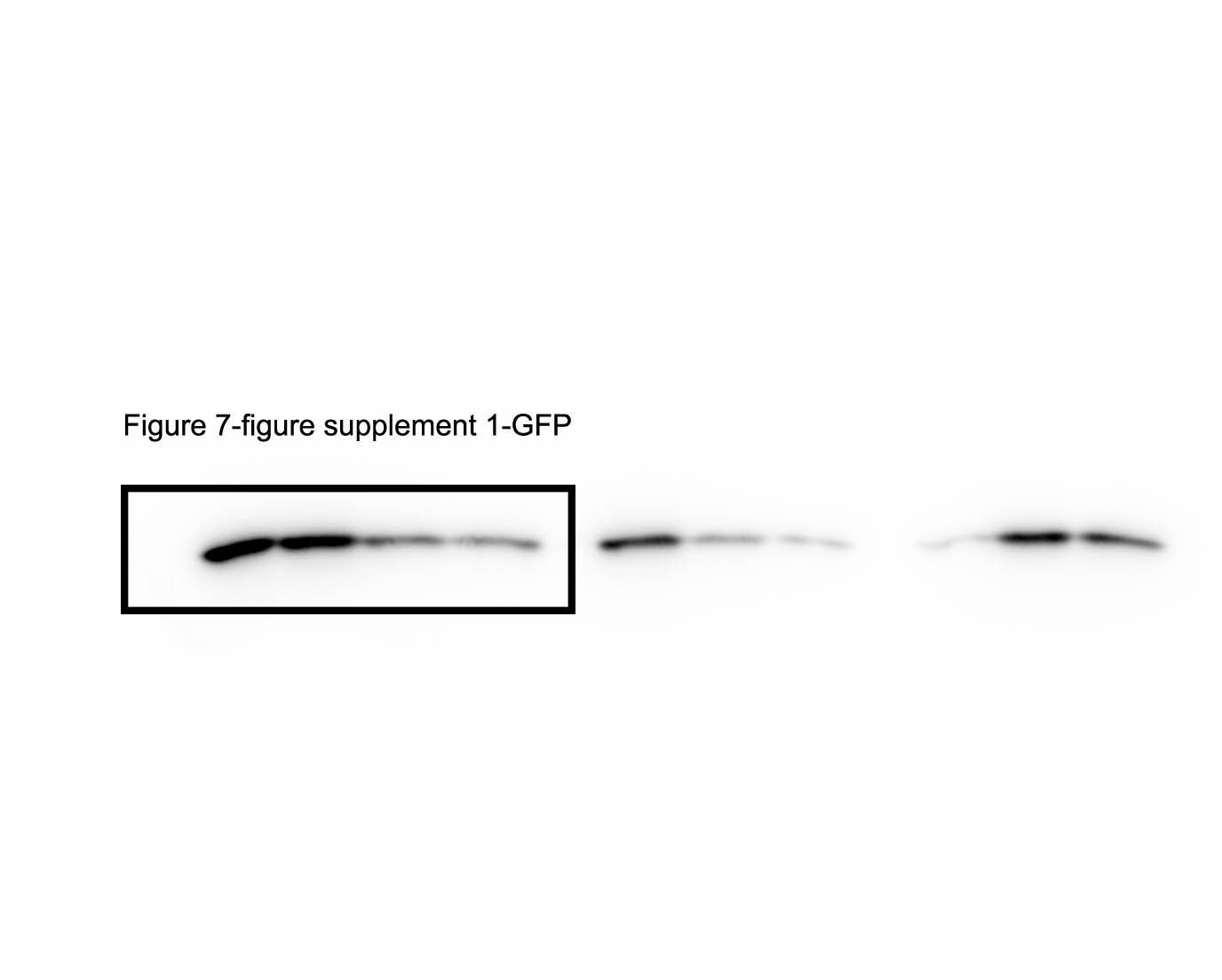

Supplement: Figure 7—figure supplement 1—source data 3. [file elife-94898-fig7-figsupp1-data3.zip › Figure 7-figure supplement 1-GFP.jpg]
